# Systematic partisan content skews in TikTok during the 2024 US elections

**DOI:** 10.1038/s41586-026-10447-1

**Published:** 2026-05-06

**Authors:** Hazem Ibrahim, HyunSeok Daniel Jang, Nouar Aldahoul, Aaron R. Kaufman, Talal Rahwan, Yasir Zaki

**Affiliations:** 1 Department of Computer Science, New York University Abu Dhabi, Abu Dhabi, United Arab Emirates; 2 Department of Political Science, New York University Abu Dhabi, Abu Dhabi, United Arab Emirates

**Keywords:** Social sciences, Computational science

## Abstract

Social media platforms increasingly mediate political information exposure, yet the role of algorithmic curation in shaping political exposure remains contested^1,2^. This question is difficult to resolve on platforms in which users retain substantial control over their feeds^3,4^. The ‘For You’ feed of TikTok, which delivers content almost entirely through algorithmic recommendation, offers a setting in which user agency is sharply constrained. Here we show, through 323 audit experiments with controlled ‘sock puppet’ accounts seeded with Democratic or Republican content across three US states, that accounts seeded with partisan content exhibited systematic, asymmetric differences in partisan exposure. Across more than 280,000 recommendations collected over 27 weeks during the 2024 US presidential election campaign, Republican-seeded accounts received about 11.5% more co-partisan content than Democratic-seeded accounts, whereas Democratic-seeded accounts were exposed to about 7.5% more cross-partisan content—largely anti-Democratic material—even after adjusting for engagement metrics. These asymmetries are concentrated among high-reach Republican channels and in specific policy domains, including immigration, crime and foreign policy for Democrats, and abortion for Republicans. Our findings show partisan imbalances in political information exposure on a platform dominated by algorithmic recommendations, with implications for platform governance and democratic discourse.

## Abstract (web summary)

Audit experiments on TikTok show asymmetric partisan exposure that is not explained by observable engagement metrics, with Republican-leaning accounts receiving more aligned content and Democratic-leaning accounts more cross-partisan recommendations.

## Main

Digital platforms now structure much of political communication at a global scale, making the dynamics of algorithmic recommendation systems central to questions of public policy, election integrity and democratic governance^5–8^. A large body of work examines whether recommendation algorithms amplify partisan preferences or steer users towards ideologically congruent material, reinforcing echo chambers and filter bubbles^9–13^. On platforms such as YouTube, Twitter, Facebook and Instagram, studies have documented ideological segregation in viewing patterns and have shown that feed-ranking systems shift the balance of content exposure^2,14–17^.

A contrasting line of research emphasizes user agency: people self-select into ideologically congenial spaces, and algorithmic diversification may have limited impact as users return to preferred viewpoints^1,3,18^. Some evidence suggests online news consumption is more diverse than offline^19–21^, and experimental deactivation of Facebook and Instagram before the 2020 US elections had no measurable effect on polarization^4^. Both perspectives must contend with evidence of asymmetric content polarization: conservative users are more likely to inhabit homogeneous networks and encounter misinformation^16,17^, raising the question of whether this reflects self-selection consistent with personality-level differences^22,23^ or asymmetric algorithmic amplification.

This tension is particularly difficult to disentangle on platforms in which users retain considerable agency over what they see. We therefore study TikTok, where the ‘For You’ feed delivers content almost entirely through algorithmic curation with limited user control, making it a valuable setting for measuring partisan exposure patterns in an environment in which content delivery is algorithmically mediated. Despite the rapid growth of TikTok, it remains relatively underexplored compared with Facebook^1,15^, YouTube^24,25^ and Twitter^26^. Previous work on TikTok has examined user demographics^27^, party communication strategies^28^ and polarization dynamics^29^, but systematic algorithmic audits remain lacking.

Here, we conduct a large-scale audit of the political content recommendations of TikTok in the lead-up to the 2024 US presidential elections. Using 323 controlled ‘sock puppet’ accounts across New York, Texas and Georgia, we collected more than 280,000 recommended videos across 27 weeks (30 April–11 November 2024). These accounts were seeded with partisan content, and recommended videos were classified through a human-validated pipeline supported by an ensemble of large language models (LLMs).

To explore the possibility of a political skew in the content recommended on TikTok, we operationalize skew as the difference in partisan recommendation rates for accounts seeded with partisan content for both major US political parties. Our findings show a significant Republican-leaning skew in the recommendations of TikTok, which persists across states even after accounting for channel- and video-level engagement metrics. By systematically ruling out plausible user-centred explanations, to the extent possible with the available data, we provide evidence that the asymmetries we observe are not explained by observable engagement metrics, although the specific mechanism—whether algorithmic rules, content supply or other factors—cannot be determined from our data. Specifically, we find that Republican-conditioned accounts received 11.5% more party-aligned content compared with Democratic-conditioned accounts across all states. Likewise, Democratic-conditioned accounts are exposed to 7.5% more cross-partisan content than their Republican counterparts, particularly content from the top-ticket political candidates of each party.

## Experimental setup

**Extended Data Fig. 1:**
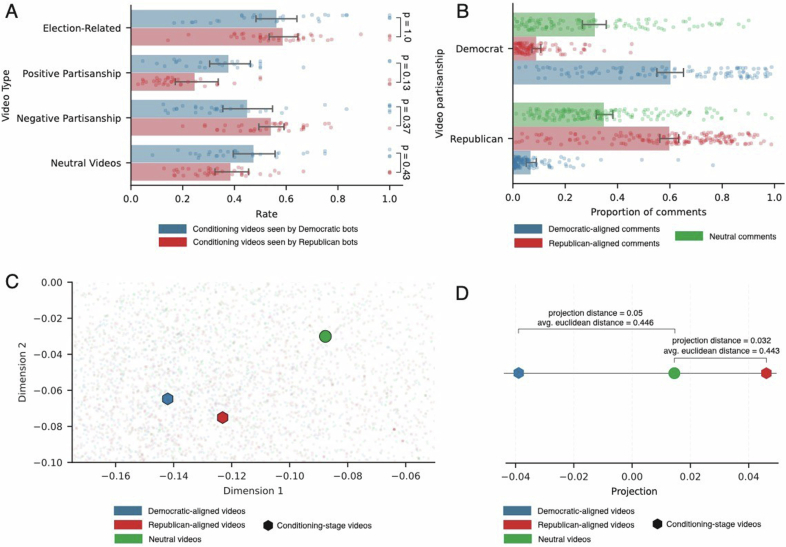
Conditioning stage validation. (**A**) The proportion of videos relating to the election (N = 73), in support of a party (N = 66), in opposition of the opposite party (N = 72), or politically neutral in tone (N = 71) seen by Democratic-aligned and Republican-aligned bots. Two-sided independent ttests are conducted. (**B**) The proportion of comments that are neutrally-aligned, democratic-aligned, and republican-aligned in videos Democratic- (N = 329) and Republican-aligned (N = 448) videos. In both **A** and **B**, Error bars denote +1/−1 standard deviations from the mean. (**C**) Two dimensional PCA mapping of the embeddings of the transcripts of Democratic-aligned, Republican-aligned during the conditioning-stage, and neutral videos during recommendation stage. (**D**) The projection of cluster centroids along the axis of maximal variation between Democratic-aligned and Republican-aligned videos.

We conduct 323 audit experiments on TikTok using sock puppets (that is, bots) that mimic user activity. Each experiment proceeds in two stages. In the conditioning stage, a bot watches a controlled sequence of politically seeded videos, teaching the recommendation algorithm of TikTok the political preferences of the bot (see Methods and Extended Data Fig. 1). In the subsequent recommendation stage, the bot watches videos on the ‘For You’ page of TikTok, whose partisan and topical content constitutes our outcome data. Each experimental run lasted 1 week: 1 day for conditioning and 6 days for recommendations (Supplementary Fig. 4).

Each bot is assigned two treatment attributes: political leaning (Democratic, Republican or Neutral) and geography (New York, Texas or Georgia, selected as prototypically Democratic, Republican and swing states, respectively). Democratic and Republican bots watched up to 400 party-aligned videos during conditioning; Neutral bots bypassed conditioning entirely to mimic apolitical users^30,31^. Each bot was connected to a virtual private network (VPN) server and Global Positioning System (GPS)-mocked to its assigned state (see Methods).

**Fig. 1:**
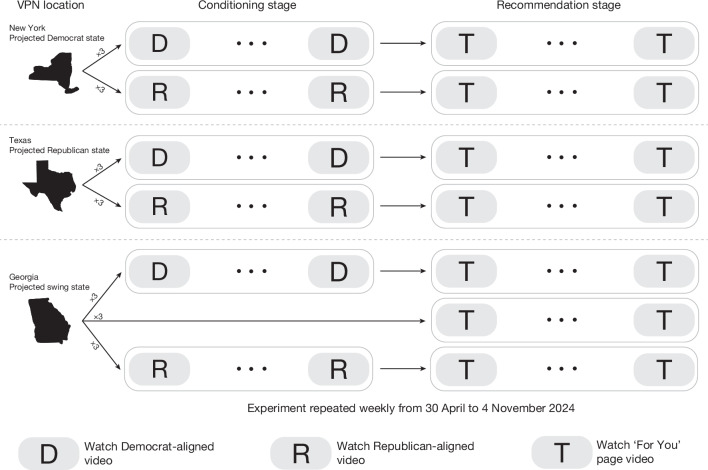
Experimental setup. Twenty-one experiment runs were performed each week between 30 April 2024 and 4 November 2024. Bots were assigned an experimental condition based on two attributes, namely, their VPN and GPS location (New York, Texas or Georgia), as well as the political leaning (Democratic, Republican or Neutral) of the videos they watched during the conditioning stage. Each bot initially passed through a conditioning stage in which it viewed up to 400 videos of a given political leaning, and then transitioned to the recommendation stage, in which it viewed up to 1,200 videos on the TikTok ‘For You’ page. US state icons were adapted from Flaticon (https://www.flaticon.com/packs/us-states-maps-14093473).

Each week from 30 April 2024 to 4 November 2024, we created 21 new TikTok accounts and assigned them to one of seven experimental conditions: Democratic, Republican or Neutral conditioning crossed with location in New York, Texas or Georgia, with three accounts per condition per week. Neutral accounts were deployed only in Georgia. Through 323 successful runs over 27 weeks, our bots watched 91,934 total (3,108 unique) conditioning videos and 284,093 (176,252 unique) recommended videos. Figure 1 shows the design; further details are provided in the Methods. The study was preregistered before data analysis (https://aspredicted.org/mjj2-vxrb.pdf).

## Measuring political content

**Extended Data Fig. 2:**
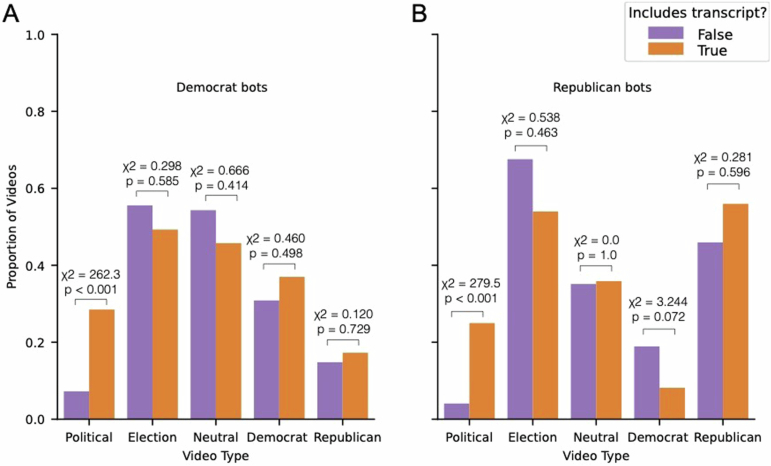
A comparison between videos with and without transcripts seen by Democrat (**A**) and Republican (**B**) bots, respectively. Two-sided *χ*^2^-tests are conducted across groups.

To categorize the partisan content of recommended videos, we downloaded English transcripts from subtitle uniform resource locators (URLs) in video metadata, available for 40,264 videos (22.8% of unique videos, 26.2% of all recommendations; see Extended Data Fig. 2 for validation on the representativeness of the sample of videos with a transcript). We then used a human-validated ensemble of three LLMs—GPT-4o (ref. ^32^), Gemini-Pro^33^ and GPT-4 (ref. ^34^)—to classify each video along three questions: (Q1) Is the video political? (Q2) Is it related to the 2024 US elections or major political figures? and (Q3) What is its ideological stance (Pro-Democratic, Anti- Democratic, Pro-Republican, Anti-Republican or Neutral)? For each question, our outcome is the majority vote across the three models; 89.4% of videos reached a majority on Q3. Details on measurement, validation and prompting are provided in the Methods; descriptive statistics are given in Supplementary Table 11.

We use these classifications to define four analysis constructs, as in ref. ^35^:

1. Positive partisanship: content that supports a party (pro-Democratic or pro-Republican), irrespective of the viewer’s partisanship.
2. Negative partisanship: content that opposes a party (anti-Democratic or anti-Republican), irrespective of the viewer’s partisanship.
3. Co-partisan recommendations: alignment of the videos matches the conditioning of the bot (for example, pro-Democratic and anti-Republican videos for Democratic bots).
4. Cross-partisan recommendations: videos whose alignment opposes the conditioning of the bot.

## Political recommendation rates

The question arises whether the partisan content recommended on TikTok is skewed towards one party over the other. Arguably, we expect the behaviour of the algorithm to fall into one of the following broad categories: stasis, symmetric polarization, asymmetric polarization and partisan bias. Under stasis, partisan-seeded accounts would receive content mirroring their conditioning, with no drift over time. Under symmetric polarization, both Democratic- and Republican-seeded accounts would receive increasing co-partisan content at roughly equal rates. Under asymmetric polarization, one group would receive a notably larger increase in co-partisan material. Under partisan bias, all accounts would shift towards a single party regardless of their conditioning. We need to assess which of these dynamics the recommendations of TikTok most closely resemble. Although our design allows us to compare co-partisan and cross-partisan recommendation rates, our findings cannot definitively determine whether asymmetries arise from user behaviour training the system or from internal algorithmic rules. What we can show, however, is that the skews we document cannot be explained by observable user engagement metrics (see Supplementary Note 3 for more details on our robustness checks). Recall that our experimental design conditions some bots to Democratic-aligned content, some to Republican-aligned content and others to no partisan content at all. By comparing the partisan content of the recommended videos of TikTok to the partisan content of the conditioning videos, we can begin to answer the question of which of these dynamics the recommendations of TikTok most closely resemble.

**Fig. 2:**
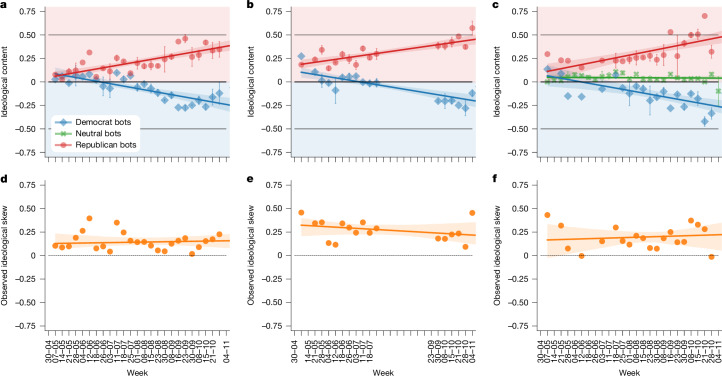
Differences in recommendation rates over time. **a**–**c**, The mean ideological alignment for bots of a given conditioning leaning in New York (**a**), Texas (**b**) and Georgia (**c**), measured as the difference between the proportion of recommended videos which are Republican-aligned minus the proportion of Democratic-aligned videos. OLS regression lines are plotted with 95% confidence intervals. Data points correspond to *n* = 108 experimental runs over 26 weeks, *n* = 84 experimental runs in 18 weeks and *n* = 131 experimental runs in 27 weeks in **a**, **b** and **c**, respectively. **d**–**f**, The observed ideological skew for each state over time; positive values indicate a Republican skew. OLS regression lines are plotted with 95% confidence intervals.

We measure the ‘ideological content’ of the recommendations of a given bot as the proportion of political recommended videos that are Republican-aligned (pro-Republican or anti-Democratic) minus the proportion of recommendations that are Democratic-aligned (pro-Democratic or anti-Republican). This allows us to score the video recommendations of each bot from −1 (all videos are Democratic-aligned) to +1 (all videos are Republican-aligned). We plot the recommendations of the bots across experimental conditions and over time in Fig. 2a–c for the states of New York, Texas and Georgia, respectively. Each point represents the mean ideological content of the recommendations received by the bots in a given week and experimental condition. Here, bars indicate 95% confidence intervals, whereas colour indicates the conditioned partisanship of the bots. Missing points represent weeks without any successful runs for bots located in a given state (see Supplementary Table 3 for the number of successful runs per condition over time). We also plot ordinary least squares (OLS) regression lines of ideological content by experimental condition over time, showing an increase in polarization in all states: partisan-seeded accounts received more co-partisan content over time. In other words, the ideological content scores for Republican-conditioned bots become increasingly positive, whereas the scores for Democratic-conditioned bots become increasingly negative. However, although both Democratic- and Republican-conditioned bots received more ideologically aligned content over time, the magnitude is significantly greater for Republicans (two-sided *t*-test: *P* < 0.001 for all three states). See Supplementary Table 12 for details on the mean monthly ideological content of Republican and Democratic bots in each state, as well as Neutral bots in Georgia. Considering that the creator accounts used in the conditioning stage did not change over the duration of our experiments, there are several possible explanations for these findings. For instance, it is possible that political debates in Congress surrounding TikTok coincided with shifts in content exposure patterns, although we cannot determine whether any platform-side changes occurred. Another possibility is that, as the election neared, users increasingly began to polarize in their content preferences as more and more politically uninterested or uninformed voters shaped their voting preferences.

Figure 2d–f collapses these party-specific trends into a single measure of ideological skew for each state and week. Here, the *y* axis shows the difference in ideological content between Republican- and Democratic-conditioned bots (the mean ideological content score for Republican bots minus the mean score for Democratic bots), in which the negative values indicate a Democratic skew and positive values indicate a Republican skew. Because both Democratic and Republican bots are moving away from zero in opposite directions at roughly similar rates in Fig. 2a–c, their difference in Fig. 2d–f remains relatively stable over time, even as the gap between the two lines in Fig. 2a–c widens. On average, Republican bots saw approximately 11.8% more co-partisan recommendations than their Democratic bot counterparts while simultaneously seeing approximately 7.5% fewer cross-partisan recommendations. Supplementary Table 13 shows results from independent *t*-tests comparing the partisan content of bots of each type of conditioning, showing that Republican bots received significantly more co-partisan content across all three states (New York: *t* = 5.83, *P* < 0.001; Texas: *t* = 9.21, *P* < 0.001; Georgia: *t* = 5.83, *P* < 0.001; all *t*-tests are two-sided), and both Republican and Democratic bots received more co-partisan content than Neutral bots (Republican compared with Neutral: *t* = 13.22, *P* < 0.001; Democratic compared with Neutral: *t* = 4.70, *P* < 0.001; all *t*-tests are two-sided). Comparing political recommendation rates broadly across states, we find that political videos were most frequently recommended in Georgia, followed by New York and Texas, although the differences in these rates across states were not statistically significant (New York compared with Texas: *P* = 1.0; New York compared with Georgia: *P* = 0.18; Georgia compared with Texas: *P* = 0.12); all *t*-tests are two-sided. Supplementary Figs. 6–9 show rolling averages of the amount of political content, election-related content, co-partisan and cross-partisan videos viewed by different bots during a given experimental run. As shown in these figures, for both Democratic- and Republican-conditioned bots, the conditioning stage results in an immediate spike in political content recommended to bots, decaying as bots are recommended more videos but never approaching the control group.

To assess whether the observed partisan asymmetries could be explained by observable engagement metrics, we conduct a series of robustness checks, which are provided in Supplementary Note 3. Specifically, we construct 48 engagement-based counterfactual models in which recommendations are driven solely by observable video- and channel-level metrics (for example, likes, comments, plays and follower counts), examine whether asymmetric cross-party engagement in comment networks could generate the skew, estimate bot-video-level regression models controlling for state, time, conditioning intensity, transcript availability and engagement covariates, and perform a sensitivity analysis quantifying how strong any unobserved engagement signal would need to be to eliminate the observed gap. Across these tests, the Republican-leaning skew remains substantially larger than predicted by engagement-based counterfactuals and robust to observed covariates.

## Negative partisanship

Recall that we previously grouped pro-Democratic and anti-Republican videos as Democratic-aligned and pro-Republican and anti-Democratic videos as Republican-aligned. Next, we unpack the observed partisan skew into videos in positive partisanship (that is, those in favour of a party) and negative partisanship (that is, those in criticism of a party).

**Fig. 3:**
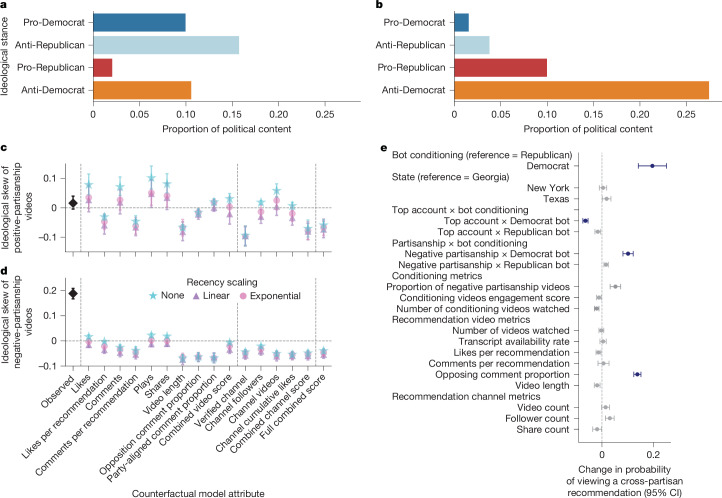
Recommendation rates based on ideological stance. **a**,**b**, The mean proportion of videos of a given partisan (non-neutral) ideological stance recommended to Democratic bots (**a**) and Republican bots (**b**). **c**,**d**, Observed ideological skew and the expected ideological skew based on counterfactual models when considering only pro-party videos or anti-opposition videos, respectively. *n* = 1,002 simulations across 17 counterfactual models and three scaling factors. Error bars denote 95% confidence intervals from the mean expected ideological skew. **e**, Linear probability model estimates on the percentage-point change in the probability of viewing a cross-partisan recommendation, when accounting for the type of video, as well as interaction effects between the video type and the conditioning of the bot. Statistical significance is measured using *t*-tests. Statistically significant coefficients after Benjamini–Hochberg corrections are highlighted in navy blue. Unadjusted *P* values and *P* values after Benjamini–Hochberg multiple comparison corrections can be found in Supplementary Table 26. Standard errors are clustered by bot (that is, the unique account–device pair used in each experimental run) and the week in which the experiment was conducted. CI, confidence interval.

We plot the distribution of individual partisan (non-neutral) stances recommended to Democratic-conditioned bots and Republican-conditioned bots in Fig. 3a,b, respectively. As shown in Fig. 3a, Democratic-conditioned bots were recommended anti-Republican videos (light blue) more frequently than pro-Democratic videos (dark blue). Similarly, Fig. 3b shows that Republican-conditioned bots were recommended more anti-Democratic videos (orange) than pro-Republican ones (red), indicating a general negative partisanship skew^36^. However, this skew was greater for Republican bots than for Democratic ones (Republican bots: two-sided *χ*^2^ = 507.3,* P* < 0.001; Democratic bots: two-sided *χ*^2^ = 85.6, *P* < 0.001). Negative partisanship videos generally demonstrated higher absolute engagement (views and likes) on average compared with positive partisanship videos. However, when controlling for viewership through per-view analysis, engagement rates were largely comparable between the two categories, with comments per view being higher for positive partisanship content (see Supplementary Table 28 for the average engagement received by videos of each ideological stance and grouped by partisanship).

Looking at cross-partisan recommendations specifically, we find that Democratic-conditioned and Republican-conditioned bots were almost equally as likely to see positive partisanship videos of the opposite party (pro-Republican 2.9% and pro-Democratic 2.3%, respectively; two-sided *χ*^2^ = 4.94,* P* = 0.03). However, Republican bots were significantly less likely to see anti-Republican content than Democratic bots were to see anti-Democratic content (5.8% and 14.2%, respectively; two-sided *χ*^2^ = 234.25, *P* < 0.001), suggesting that the cross-partisan recommendations were driven specifically by anti-Democratic videos.

Considering this gap, we recompute the ideological skew measured in Supplementary Fig. 1a, once while considering only positive-partisanship (pro-Democratic and pro-Republican) videos, and again while considering only negative-partisanship (anti-Democratic and anti-Republican) videos. For each of these sets of videos, we also recompute the expected ideological skew based on the different engagement metrics these videos receive (Supplementary Tables 15 and 16 detail the ideological skews as measured by each of these models, respectively). The results of these analyses are presented in Fig. 3c,d for positive- and negative-partisanship videos, respectively. Broadly speaking, the Republican skew observed in positive-partisan videos is markedly smaller than that in negative-partisan videos. We further tested whether partisan asymmetries are driven specifically by the interaction between party and negativity. Figure 3e presents regression estimates in which we stratify videos by both party and partisanship type. We find that anti-Democratic videos stand out: they are significantly more likely to be cross-partisan recommendations than any other video type, even after controlling for engagement metrics and fixed effects (coef = −0.0641; *P* < 0.0001). This confirms that the partisan skew we document is not simply Republican compared with Democratic, or positive compared with negative, but specifically concentrated in anti-Democratic content. See Supplementary Table 26 for the regression table containing these results with both adjusted and unadjusted *P* values.

We also examine whether the observed asymmetries are concentrated in particular policy domains rather than spread uniformly across channels and across types of political content (Supplementary Notes 4 and 5, respectively). Using a LLM-assisted topic classification framework, we identify the substantive issues discussed in partisan videos and compare their distribution across party-aligned content and cross-partisan recommendations. The results indicate that recommendation asymmetries are topic-specific: Republican-aligned content is disproportionately concentrated in areas such as immigration and foreign policy, whereas Democratic-aligned content is more prevalent in domains such as climate change and abortion. Moreover, cross-partisan exposure is not evenly distributed, but instead clusters in a narrow set of highly salient and contentious issues. These patterns suggest that partisan exposure asymmetries are concentrated within specific policy domains rather than distributed uniformly across political content.

## Survey on perceived changes to TikTok political content

The experimental results above reveal systematic partisan skews in the political content recommended by TikTok, but an important question remains as to whether these imbalances in exposure are perceptible to real users and whether they shape how users interpret their feeds. To assess whether the asymmetries documented in our audit correspond to users’ lived experiences of the platform, we conducted a preregistered survey of 1,008 US-based TikTok users. The survey gauges how users assess the partisan slant of their ‘For You’ feeds and whether they report patterns consistent with the co-partisan and cross-partisan imbalances observed in the audit. Participants (*n* = 1,008) were recruited by Prolific from US-based active TikTok users; the sample was not nationally representative and skewed Republican (54.8% Republican, 31.4% Democratic and 13.8% Independent), with 54.3% female, 44.5% male and 1.2% non-binary respondents; 64.1% identified as White and 27.4% as Black; 82.2% held at least a bachelor’s degree; and the largest age group was 25–34 years old (37.0%). Full demographic details are given in Supplementary Table 35.

Participants were first asked a series of three open-ended, free-response questions about perceived changes to their TikTok feeds. The first question is ‘Over the past 12 months, from March 2024 to today, how has the overall content of your TikTok feed changed?’ to gain insight into whether participants would voluntarily mention changes to political content broadly, or whether content has shifted towards a specific party. The second question focuses on political content in particular: ‘Over the past 12 months, from March 2024 to today, how was the political content of your TikTok feed changed?’ The third question is ‘Over the past 12 months, from March 2024 to today, has the political content of your TikTok feed become more positive or more negative?’ Following these open-ended questions, participants responded to six structured items measuring their perception of whether their feed had shifted towards more political or less political content, more Democratic or more Republican content, whether it had become more positive or negative, more optimistic or pessimistic, more pro- or anti-Trump, and whether they were seeing more content they agreed or disagreed with politically (for more details, see Methods).

**Fig. 4:**
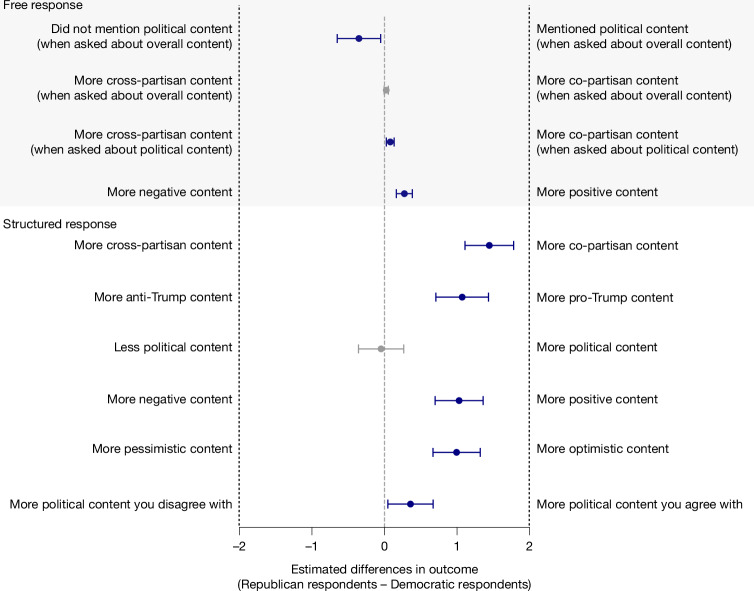
Survey responses. Regression coefficients of Republican respondents (*n* = 552) relative to Democratic respondents (*n* = 317) for different survey questions regarding content on their TikTok feeds. Positive values indicate higher values of the outcome among Republican respondents relative to Democratic respondents (controlling for age, gender, race and education). The top four rows are for responses to text-entry questions, whereas the bottom six rows are for responses to structured scale-based questions. Statistical significance is measured using *t*-tests. Statistically significant coefficients are highlighted in navy blue. Error bars denote 95% confidence intervals.

Figure 4 presents the regression coefficients of Republican respondents relative to Democratic respondents across all questions. Specifically, each of the coefficients presented is taken from a separate regression, each of which controls for participant demographics (see Supplementary Tables 33 and 34 for the full set of regression results and Supplementary Table 35 for a breakdown of participant demographics). In the free-response portion, Republican respondents were less likely than Democrats to mention political content when asked about overall feed changes (coef = −0.35; *P* = 0.02). When asked about political content specifically, Republicans were significantly more likely to mention increases in co-partisan content (coef = 0.08; *P* = 0.003) and more positive political content (coef = 0.274; *P* < 0.0001).

In the structured response section, there were clear partisan differences as well. Republicans were significantly more likely to report seeing an increase in Republican-aligned content (co-partisan content) than Democrats were to report seeing Democratic-aligned content (coef = 1.44; *P* < 0.0001). Republican respondents were also more likely to characterize the political content on their feed as positive (coef = 1.03; *P* < 0.0001), optimistic (coef = 0.99; *P* < 0.0001), agreeable (coef = 0.36; *P* = 0.02) and pro-Trump (coef = 1.07; *P* < 0.0001).

Taken together, these patterns indicate that the partisan asymmetries identified in our audit are reflected in users’ lived experiences. Republicans systematically report seeing more co-partisan and positively valenced political content than Democrats report seeing their own. This alignment between experimental and self-reported exposure suggests that the partisan skew in content exposure documented in our audit is consistent with users’ self-reported experiences on the platform.

## Discussion

Our longitudinal audit of the recommendation system of TikTok during the 2024 US presidential elections shows clear partisan asymmetries in content exposure. Across three states, Republican-conditioned accounts received about 11.5% more ideologically aligned content than Democratic-conditioned accounts, whereas Democratic-conditioned accounts were exposed to 7.5% more cross-partisan material. These findings align with previous work showing that conservative users often encounter more partisan-reinforcing content online^16,17^. However, this pattern can be interpreted in two ways. On the one hand, it may reflect user-driven behaviour, in which conservative users gravitate towards more ideologically aligned content, a pattern that recommendation algorithms adapt to. On the other hand, the asymmetry may arise, in part, from how the recommendation environment of the platform interacts with the available content ecosystem, disproportionately reinforcing co-partisan exposure for right-leaning accounts. Our experimental design does not allow us to fully disentangle these mechanisms. Nonetheless, because all accounts in our study followed comparable conditioning protocols and observable engagement metrics cannot account for the asymmetry, the imbalance in partisan content exposure is unlikely to be solely attributable to user choice.

Partisan asymmetries also extend to the tone of recommended content. Videos attacking political opponents appeared more frequently in recommendations than videos supporting one’s own side, with anti-Democratic content appearing more frequently than anti-Republican content. This pattern aligns with previous research on the psychological appeal of oppositional messaging^36^, suggesting that the observed exposure skew is compounded by the style and tone of political communication. Survey evidence further indicates that users perceived these imbalances, in which Republican respondents reported seeing more ideologically agreeable content than Democratic respondents.

Our results add to a mixed body of evidence on ideological bias across platforms. Previous work has shown that conservatives on Facebook are more likely to operate within insular networks and encounter misinformation^16^, whereas studies on Twitter and YouTube point to both partisan amplification and user migration towards ideologically congenial content^12,37^. At the same time, experimental studies on Facebook, Instagram and YouTube suggest that self-selection often outweighs algorithmic influence, with limited downstream effects on attitudes or behaviour^2–4^. We reconcile these mixed findings by focusing on TikTok, a platform in which self-selection is sharply constrained. Because users have little control over their feeds, the asymmetries we observe are less likely to be driven by user self-selection and more likely to reflect factors inherent to the content delivery environment of the platform.

Our findings carry broad implications for both partisan politics and platform governance. TikTok has become an important news source for young voters, a demographic that shifted 10 percentage points towards Trump between 2020 and 2024 (ref. ^38^). Because recommendations dominate the TikTok experience, asymmetries in exposure may shape the information environment of a politically consequential audience. These patterns also raise regulatory questions. Under the EU Digital Services Act, large platforms are required to assess and mitigate systemic risks to electoral processes, whereas in the USA, First Amendment protections grant platforms far greater editorial discretion^39^. The contrast highlights both the urgency and difficulty of developing governance frameworks for partisan content exposure asymmetries on platforms, particularly when those platforms achieve monopolistic scale in highly polarized contexts.

### Limitations

Although we made every effort to scrutinize our results, several empirical and technical limitations remain. First, our experiments cannot fully capture the complexity of real user behaviour or the full range of factors influencing recommendations, including non-public engagement metrics accessible only to TikTok. Second, our classification of political and topical content relies on LLM-based stance detection. Although validated in previous work^40,41^, this approach is not perfectly accurate; we mitigated potential biases by using an ensemble of three models and majority voting. Third, because our annotations relied on video transcripts, we did not capture visual or audio cues that may also convey political messaging. Fourth, our analysis focuses exclusively on English-language content, as our transcript-based pipeline relies on English subtitles. We, therefore, do not capture political videos in Spanish or other languages that may circulate within linguistic minority communities in the USA. Recommendation dynamics and partisan exposure patterns in these information environments may differ, limiting the generalizability of our findings beyond English-language political content. Fifth, our survey captures a sample of 1,008 US-based TikTok users, and this sample is not representative of the broader US population. Finally, after 11 November, TikTok increased its bot-detection efforts, preventing additional data collection. This limited our ability to extend the analysis into the post-election period or to assess whether partisan skew changed after the election.

A further limitation concerns possible asymmetries in the conditioning stage of our audits. Although seed videos were comparable across partisan groups in semantics, partisanship and engagement features, the source accounts differed: 8 of the 12 Democratic seeds came from official office holders, whereas the Republican set skewed towards independent creators. If TikTok clusters users partly on latent account profiles or community structures, our bots could inherit these differences despite conditioning on otherwise comparable videos. This possibility should be kept in mind when interpreting our results. Furthermore, our standardized conditioning protocol also abstracts from real-world differences in how Democrats and Republicans engage with political content online. Previous research suggests that partisan groups differ in information-seeking behaviour and interaction patterns. By holding engagement constant across bots, we isolate the content exposure patterns that arise in response to partisan signals but do not capture how naturally occurring behavioural differences may shape recommendation dynamics.

More fundamentally, our study examines content polarization, or disparities in what partisan-aligned content users are exposed to, rather than affective polarization or downstream changes in political attitudes, beliefs or behaviours. Our audit design allows us to identify systematic differences in the partisan content delivered to users, but it does not assess whether these differences meaningfully shape how users feel about opposing partisans, their policy preferences or their broader civic engagement. Understanding these downstream consequences requires linking exposure patterns to psychological and behavioural responses, which lies outside the scope of the present study but is essential for assessing the societal impact of recommendation systems^42,43^.

Future research could address these limitations in several ways. First, combining sock puppet audits with real user data, as in ref. ^3^, could provide a more detailed picture of how the algorithm of TikTok responds to genuine user behaviour. Second, developing methods to analyse visual and audio features alongside transcripts would capture political messaging beyond text. Third, longitudinal studies extending beyond the election period could distinguish election-specific effects from more persistent patterns. Fourth, comparative audits across platforms would help isolate TikTok-specific dynamics from broader trends in political communication. Finally, larger datasets on misinformation, particularly around the 2024 US elections, are needed to assess its prevalence and partisan asymmetries on TikTok and other platforms, especially as Meta phases out its third-party fact-checking programme^44^.

Our experimental design also required concessions because of practical constraints. First, we were unable to include a Neutral seeding condition in New York and Texas, which would have provided more accurate baselines, as expanding the audits further increased the risk of detection and deactivation. Second, our design also captures only content exposure patterns during the earliest stages of user engagement on the platform. Each bot account in our study interacted with a relatively small set of partisan videos before entering the recommendation phase. As such, our findings reflect only ideological skew and partisan dynamics in content exposure during the initial stages of account activity, rather than long-term platform behaviour. This choice is ultimately a trade-off, allowing us to study changes in this initial content curation stage of a user’s life cycle over the course of 6 months, at the expense of not tracking asymmetries that may occur with longer-tenured users. Understanding how ideological patterns evolve over a longer duration, and how initial skew compounds or attenuates with prolonged engagement, remains an important direction for future work. Finally, all bot accounts were standardized to an age range of 22–24 years to minimize profile variability and align with typical young adult users. Although intentional, this limits the generalizability of our findings to other age groups, particularly adolescents and older adults, whose algorithmic experiences may differ.

## Methods

### Ethics statement

Throughout this study, we took care to follow relevant ethical standards. The use of sock puppet accounts is an established research technique for investigating personalization and bias on internet platforms^12,24,45^, provided it is strictly for noncommercial, public-interest purposes and does not compromise user privacy. Previous work and legal precedent have indicated that the Terms of Service of social media platforms, which may prohibit automated access to content, do not necessarily conflict with collecting publicly available data for research aims^46,47^. Given our focus on potential impacts on democratic processes and political discourse, this project falls under well-recognized academic exceptions for studying online information ecosystems. Moreover, we minimized the risk of privacy breaches or commercial harm by restricting our data collection to publicly available content. Although the experiment itself did not include any human participants, we obtained informed consent from the human annotators for our LLM classification validation tasks.

For the survey component of this study, the survey was deemed exempt by the authors’ institutional review board (IRB protocol no. HRPP-2025-69).

### Experimental setup

In this experiment, we aimed to measure the rate at which videos of a certain political leaning appear in the recommendations of TikTok, in the context of US politics. To do so, we use bots that simulate TikTok users by watching both predefined sequences of videos of a given political leaning (the ‘conditioning’ stage), and then subsequently watching recommended videos on ‘For You’ page (the ‘recommendation’ stage) of a given bot. Each experimental run, comprising these two stages, lasts for a 1-week period.

Over the duration of 27 weeks, 567 experiments were conducted. Specifically, each week, 21 new TikTok accounts are created by randomly combining the most common American first and last names from ref. ^48^ and assigning an age between 22 years and 24 years. This allowed each account to impersonate a potential voter for the US presidential election likely to be active on TikTok. This age range was also an intentional decision made to standardize the age of the user across experimental conditions. Moreover, our decision to select this age range was guided by data suggesting that the 18–24 years age bracket had the largest share of users on TikTok in the USA as of 2022 (35%), although the older 25–34 years age bracket now occupies the largest share as of late 2024 (ref. ^49^). The 21 accounts created each week are split into one of nine experimental conditions, which are defined by two attributes. The first attribute is the state to which the bot is manually geo-located, which is either New York, Texas or Georgia (Georgia was largely regarded as a key swing state for 2024 US presidential elections, with a narrow 0.23% margin in favour of Joe Biden in the previous 2020 elections). We chose New York, Texas and Georgia because, on the basis of the 2020 presidential results, they serve as clear prototypes of a reliably Democratic state, a reliably Republican state and a competitive swing state, respectively, in the 2024 electoral landscape. Practical constraints also shaped this choice, as only these three locations were simultaneously available and stable across our VPN infrastructure. Among the feasible options, they maximized geographic and partisan diversity, but we caution that our findings should not be presumed to generalize beyond these states. The second variable is the political leaning of the videos the bot watches in the conditioning stage. The videos watched in the conditioning stage are published by either known Democrat-supporting channels or Republican-supporting channels. Finally, each week, three bots geo-located to Georgia bypass the conditioning stage of the experiment and move directly to the recommendation stage. This is done to collect recommendations made to users who do not have a particular interest in politics. A summary of the experimental conditions is provided in Supplementary Table 2.

Supplementary Fig. 4 shows a more detailed timeline of a bot during a given experimental run. Although our original design contemplated a fully crossed 3 (State: New York, Texas, Georgia) × 3 (Partisan seed: Democrat, Republican, Neutral) structure, we ultimately implemented a reduced version with seven conditions. In particular, neutral-seeded accounts were deployed only in Georgia. This decision was driven by practical constraints: expanding to nine concurrent experimental conditions required more devices to run simultaneously, which increased the risk of detection and deactivation by the platform safeguards of TikTok. We prioritized the inclusion of a neutral baseline in Georgia, a prototypical swing state, given its strategic value for interpreting partisan effects in a politically heterogeneous context. Although this design does not allow for full within-state comparisons between neutral and partisan accounts in New York and Texas, we note this as a limitation and interpret state–partisanship interactions with appropriate caution.

#### Pre- and post-experiment protocols

TikTok infers user location through the GPS or network (IP) geo-location of the device^50^. This required us to dedicate an Android smartphone, namely, Samsung Galaxy A34 5G, to each of the 21 accounts created every week. Before each experiment, we controlled device geo-location across three target states using a combined approach of GPS mocking and VPN tunnelling. Specifically, we used AnyTo^51^ for GPS coordinate spoofing, setting New York bots to ⟨40.7308, −73.9976⟩ in Manhattan, New York City; Texas bots to ⟨33.148, −96.638⟩ in Collin County; and Georgia bots to ⟨33.961, −84.537⟩ in Cobb County. These specific locations were chosen as counties that voted strongly Democrat, Republican or were a close call in the 2020 US presidential elections, respectively. Furthermore, to align the network identity of each bot with the geo-location of the intended state, we tunnelled the public IP address of each phone to one of three custom VPN servers we hosted on third-party cloud providers. We avoided commercial VPN services to minimize the risk of TikTok identifying the IPs as virtual. We installed TikTok from Google Play Store only after the GPS and IP address of each phone had been appropriately modified. At the conclusion of a weekly experiment, we factory-reset every phone before beginning the next round of the experiment. This step ensures that any TikTok-related cache is cleared and does not influence the subsequent experiments conducted on the same phones. Finally, all phones operated on Android 13, which re-randomizes the MAC (medium access control) address every 24 h (ref. ^52^), precluding the possibility of device-level tracking or bot detection of TikTok throughout a weekly experiment.

#### Conditioning stage

In the conditioning stage of the experiment, bots watch a sequence of videos published by TikTok channels aligned with either Democratic or Republican political leanings. To collect the channels that the bots would watch in this stage, channels were compiled iteratively by searching for politically charged keywords, such as ‘Trump’, ‘Biden’ or ‘Kamala’ on the search bar of TikTok, apart from the terms ‘Democrat’ and ‘Republican’. From there, the channels published videos supporting each candidate were compiled and verified by the authors to fit the following criteria: (1) most of the videos published by the channel concern political content; and (2) all the political videos published by the channel are aligned with either the Democratic or Republican political parties.

In total, 54 candidate channels were compiled across both political party affiliations. Each channel associated with a given political party was then matched with a channel from the opposite political party based on its number of followers and cumulative number of likes using Euclidean distance matching. This was done to account for the possibility that the strength of the political conditioning of a given bot may be partially attributed to the popularity of the channels it watches in the conditioning stage. After matching, the 10 pairs of channels with the smallest Euclidean distance were selected. Supplementary Table 36 details the channels used in the conditioning stage, showing 12 pairs in total, as two channels (donaldtrumpwasright and kayetriots) became no longer available (deleted or made private) during our experiments. As such, those two pairs were replaced with the new pairs that had the smallest Euclidean distance. The accounts of the main political candidates (kamalaharris and realdonaldtrump) did not exist when the experiment began in May, and hence, were not included as conditioning channels. Kamala Harris officially joined TikTok in July, whereas Donald Trump joined the platform in June.

In each experiment, a bot is conditioned to lean either Democratic or Republican by watching up to 50 most recent videos from eight randomly selected channels aligned with the respective political party. The target of each bot watching 400 conditioning videos in total was not always achieved, as shown in Supplementary Table 37. On average, Republican bots viewed fewer conditioning videos because of several of the Republican accounts having fewer than 50 total published videos, particularly during the first few months of the experiment. To account for this, the number of videos watched during the conditioning stage was controlled for during all analyses that compared recommendation rates across experimental conditions. Every video during the conditioning stage was watched for 1 min, ensuring consistent exposure to the content from the selected channels. After completing the predefined set of videos, the bot would then ‘sleep’ for 24 h, during which it would not open or interact with TikTok. This pause was implemented to simulate realistic human viewing patterns, minimizing the risk of bot detection that could result from consuming an excessive number of videos in rapid succession.

#### Recommendation stage

Following the conditioning stage, each bot in the experiment transitions to the recommendation stage, in which they watch videos that appear on their For You page. The For You page is the default interface on TikTok, in which users can watch videos recommended to them based on their interests as implicitly determined by the algorithm of TikTok. In this stage, each video is watched for up to 10 s, after which the URL of the video is retrieved. To circumvent the bot detection mechanism of TikTok, only the first 10 recommended videos were watched per hour, followed by a 60-min sleep. Moreover, the TikTok app was reloaded before every hourly session to avoid watching videos preloaded during the previous iteration.

All URLs collected in a given experimental run are then used to retrieve the metadata of the video, including the author of the video, the description of the video and its embedded transcript, if available. Of the 176,252 unique videos watched, 40,264 had a transcript available, and it is this set of 40,264 videos that we analyse in the remainder of this study. In the ‘Data Representativeness’ section, we show that this sample is representative of the entire dataset of recommended videos.

#### Experimental run validation

Naturally, with audit experiments such as this one, experimental failures are inevitable because of issues such TikTok classifying the account as a bot and subsequently suspending the account, or internet outages. As such, to ensure the same amount of recommendation exposure for Democrat- and Republican-conditioned bots, we match bots of opposite conditioning in each state during a given experiment week. Consequently, for each pair of bots, we only consider the first *n* recommendations made to each bot, where *n* is the lesser of the total number of recommendations made to either bot. Furthermore, we only consider pairs of bots that watched at least 150 videos each. This threshold is a deviation from our preregistration, which set the threshold at 500, because of our initial overestimation of the number of videos bots would watch during a given experimental run.

There were a handful of weeks in which the bots failed to meet our inclusion threshold. First, at the end of July and the beginning of August, bots with a Texas geo-location lost internet connectivity while the authors were not available to tend to the bots. As such, we were unable to collect data from Texas during this time. In another week, some accounts were recognized as bots by the bot detection algorithm of TikTok and subsequently suspended before reaching the threshold of 150 videos. Of the 567 experiments conducted, 323 met our inclusion criteria. Supplementary Table 3 lists the total number of bots that met the inclusion criteria on a weekly basis across the different bot conditioning and geo-location classes.

### Ideological stance classification

To analyse the ideological stances present in the video content, we implemented a three-step classification approach using an ensemble of LLMs comprising GPT-4o, Gemini Pro and GPT-4. First, each video transcript was evaluated for political content using the prompt: ‘Given the following video transcript, do you think the topic is political?’

For transcripts identified as political, we conducted two additional classification steps. The first assessed election relevance through the prompt ‘Given the following video transcript, do you think the topic is related to the 2024 US election or related to Donald Trump, Kamala Harris, Joe Biden, JD Vance, or Tim Walz? Answer with only Yes or No’.

The second step determined the partisan stance using the prompt ‘Given the following video transcript, classify the transcript into one of the following categories:

Anti Democrat

Anti Republican

Pro Democrat

Pro Republican

Neutral’.

The distribution of classifications across these categories is presented in Supplementary Table 11.

Throughout our study, we often group the categories ‘Anti-Democrat’ and ‘Pro-Republican’ under the broad category of ‘Republican-aligned’, and similarly group the categories ‘Anti-Republican’ and ‘Pro-Democrat’ under the category of ‘Democrat-aligned’.

To ensure classification reliability, we used a consensus-based approach in which GPT-4 served as the tiebreaker in cases of disagreement between GPT-4o and Gemini Pro. The inter-model agreement rates for each classification task are detailed in Supplementary Table 7. This ensemble method was chosen to mitigate individual model biases and enhance the robustness of our ideological stance classifications.

Finally, after all videos had been classified, we excluded the 10 advertisement videos encountered by the bots during the entire duration of the experiment, which were labelled as political. Of these, only one had a non-neutral ideological stance (‘Anti-Democrat’). Furthermore, none of the 10 advertisements was published by an official political candidate channel.

#### Ideological stance validation

To validate our automated classification approach, we conducted a human annotation study on a subset of 500 randomly selected transcripts. Three independent annotators, all political science undergraduate students, were recruited to perform the classification tasks. The annotators were permitted to use web searches to research unfamiliar topics or references within the transcripts, ensuring informed labelling decisions. Each transcript received independent classifications from all three annotators following the same categorical framework used in the LLM classification. The results of this validation study, including inter-rater reliability metrics, human majority–LLM majority classification accuracy, Cohen’s κ coefficient comparing human-majority and LLM-majority decisions, and the F1 scores of the LLM ensemble, are presented in Supplementary Table 8, whereas Supplementary Table 9 details these scores for each model separately.

#### Channel classification

To identify channels potentially leaning towards either the Democratic or Republican ideologies, we focused on channels that had published videos previously labelled with a specific non-neutral ideological stance (for example, ‘Anti-Democrat’, ‘Pro-Republican’, ‘Anti-Republican’ or ‘Pro-Democrat’). From this process, we identified 170 unique channels for analysis. For each channel, we calculated the proportion of videos aligned with the ideology of a particular party as a fraction of the total videos collected for that channel. To ensure robustness in our classification, channels were labelled as Democrat-aligned or Republican-aligned if more than 75% of their analysed videos were ideologically consistent with one party. For 85 channels with fewer than 10 unique videos collected during the experiment phase, we used TikAPI (https://tikapi.io/) to retrieve up to 30 additional videos published before the election. These additional videos were processed through the same transcript classification pipeline to determine their ideological stance. This step was necessary to avoid potential misclassification caused by the unique videos watched by the bots of a limited number of channels. Supplementary Table 38 presents a summary of the classified channels, including the total number of channels in each category (Republican-aligned, Democrat-aligned and Neutral) as well as the average proportion of partisan content for each group. Finally, we validated our 75% threshold by examining 13 channels falling between 60% and 75% alignment, which predominantly comprised traditional news outlets (*Daily Mail*, *USA TODAY* and Channel 4), talk shows (*The Daily Show*, *Don Lemon* and *The Problem With Jon Stewart*), and journalistic content that often covers political topics while maintaining some editorial balance. This distribution confirms that the 75% threshold effectively distinguished consistently partisan channels from those with occasional political lean (see Supplementary Table 5 for a list of channels that did not meet the 75% threshold).

#### Comment classification and asymmetric homophily

To identify the ideological stance of the comments on a given TikTok video, we follow a similar methodology to that of classifying a video. Specifically, we pass both the transcript of the video and the comment in question in a prompt to GPT-4o, which can be seen below: ‘Given the following TikTok video transcript and comment, classify the comment into one of the following categories.

Transcript of the video: TRANSCRIPT

Comment: COMMENT

Categories:

Anti Democrat

Anti Republican

Pro Democrat

Pro Republican

Neutral’.

The outputs of the model were validated by three independent annotators, again, all political science undergraduate students. The results of this validation process are shown in Supplementary Table 10.

To examine whether cross-party engagement patterns (‘asymmetric homophily’) could explain the observed skew seen in Fig. 2d–f, we analyse the partisan composition of comments on political videos. The key hypothesis is that, if Democrats systematically engage more with Republican-aligned videos than Republicans engage with Democratic-aligned videos, Republican-aligned videos that attract more Democratic-engaged comments should in turn be recommended more often to Democratic bots.

Specifically, we collect a random sample of 500 comments from all political videos examined in our study and classify the content of each comment in the context of that video. From this, we compute the proportion of comments ideologically aligned with the video. We find meaningful differences between Republican-aligned and Democratic-aligned videos in the composition of comments, but not in the expected direction. We find that Democratic-aligned videos have a nominally higher proportion of Republican-aligned comments than vice versa, although this difference is not statistically significant (12.9% compared with 8.5%; two-sided *χ*^2^ = 2.32, *P* = 0.13). Democratic-aligned videos also have a nominally higher proportion of co-partisan comments than Republican-aligned videos, again without reaching statistical significance (63.1% compared with 51.6%; two-sided *χ*^2^ = 1.97, *P* = 0.16). However, Republican-aligned videos have a significantly higher proportion of ‘neutral’ comments that are not explicitly partisan (44.6% compared with 26.3%; two-sided *χ*^2^ = 7.53, *P* = 0.006). We also include this metric in our weighted sampling robustness check (Fig. 2g), finding similar results to our other observed engagement metrics.

### Conditioning stage validation

We take several steps to validate that the seeding delivered to bots of opposite party alignments was equivalent. A summary of this analysis is presented in Extended Data Fig. 1. First, we evaluate the proportion of videos directly related to the US election or major political figures (as determined by the second question of the transcript classification pipeline described above). As shown in Extended Data Fig. 1a, we find that Democratic-aligned and Republican-aligned bots were exposed to a statistically similar proportion of election-related videos (two-sided independent *t*-tests; *t* = −0.478, *P* = 1.0).

Regarding positive and negative partisanship, although we find no statistically significant differences between the two bot groups (two-sided independent *t*-tests; positive partisanship: *t* = 2.19, *P* = 0.129; negative partisanship: *t* = −1.7, *P* = 0.373; and neutral: *t* = 1.63, *P* = 0.426), we do nonetheless see a modest asymmetry, in which the Republican seed set contained more anti-Democratic than pro-Republican content, whereas the Democratic seed set was comparatively balanced. Although this difference was not statistically significant, it is nonetheless important to acknowledge. This asymmetry does not reflect an intentional experimental design choice. Rather, it reflects the supply of content from the accounts selected for seeding, in which anti-Democratic videos were marginally more prevalent among the chosen Republican-aligned creators than anti-Republican videos were among the chosen Democratic-aligned creators.

To assess whether this difference had downstream effects on the proportion of negative partisanship videos seen during the recommendation stage, we model the likelihood of a recommended video being of negative partisanship as a function of the proportion of these videos seen during the conditioning stage of a bot. We find that it is neither the conditioning of the bot nor the proportion of negative partisanship videos seen in the conditioning stage that significantly predicts a recommended video being of negative partisanship. Rather, it is the characteristics of the author of the video (their follower count and total share count), as well as the normalized number of comments the video receives that are significant, suggesting that the gap in negative partisanship recommendations is due to supply-side factors rather than factors in our control in the conditioning stage (Supplementary Fig. 10). Nonetheless, we recognize that this imbalance could contribute, at least partially, to the asymmetric outcomes we observe, and we highlight this as a limitation of our design.

Second, we classify a random sample of up to 500 comments posted under each of the partisan videos seen during the conditioning stage, using the comment classification procedure described in the previous section. As shown in Extended Data Fig. 1b, videos shown to Democratic- and Republican-aligned bots received similar rates of both party-aligned and opposite-party-aligned comments (party-aligned comments: 60.2% compared with 59.9%, two-sided *χ*^2^ = 0.008, *P* = 0.925; opposite party-aligned comments: 8.9% compared with 6.8%, two-sided *χ*^2^ = 0.18, *P* = 0.67).

Third, we compute the embedding of the transcripts of all videos within our dataset using the embedding model proposed in ref. ^53^, chosen as an open-source embedding model with performance on par with state-of-the-art models. We then compute the centroids of the sets of videos seen by Democratic-aligned and Republican-aligned bots during the conditioning stage of their respective audits. The projection of these cluster centroids into two dimensions using principal component analysis (PCA) is shown in Extended Data Fig. 1c. To better understand the extent to which the sets of videos diverge semantically across partisan conditions, we compute the axis of maximal variation between Democratic and Republican videos by taking the unit vector connecting their respective centroids in the original embedding space. We then project the corresponding centroids onto this axis, allowing us to quantify and visualize partisan separation in semantic space. This approach enables us to assess whether the videos shown to Democratic- and Republican-aligned accounts are meaningfully distinct in content, or whether they largely occupy overlapping regions of the embedding space. As shown in Extended Data Fig. 1d, the cluster centroids of conditioning-stage videos seen by Democratic-aligned and Republican-aligned bots were approximately equidistant from the centroid of neutral videos, both in terms of their projected positions along the axis of maximal variation (0.05 compared with 0.032), as well as the average Euclidean distance between the individual videos belonging to that cluster and the neutral centroid in the original high-dimensional embedding space (0.446 compared with 0.443, *t* = 1.962, *P* = 0.05).

Taken together, these analyses suggest that the seeding videos shown to Democratic- and Republican-aligned bots were largely equivalent in both content and semantic distribution. Across multiple validation checks, including election relevance, partisanship framing, audience partisanship in the form of comments and transcript-level semantic similarity, we find little to no consistent evidence of systematic differences between the two groups. This is apart from the pre-seeding matching steps taken to ensure that videos were selected from channels of a similar overall popularity.

### Cross-partisan recommendation regression model

We further formalize the robustness analyses using a linear probability model (LPM) at the bot-video recommendation level. The dependent variable *Y*_*ib*_ is an indicator that video *i* recommended to bot *b* is a cross-partisan recommendation, defined as a recommendation in which the partisan alignment of the video does not match the conditioned partisanship of the bot. The key independent variable is an indicator for whether bot *b* was conditioned on Democratic compared with Republican content.

The baseline specification is

$${Y}_{ib}=\alpha +\beta {{\rm{D}}{\rm{e}}{\rm{m}}{\rm{B}}{\rm{o}}{\rm{t}}}_{b}+{\gamma }^{{\prime} }{X}_{ib}+{\delta }_{b}+{\lambda }_{w}+{{\varepsilon }}_{ib},$$

where DemBot_*b*_ equals 1 for Democratic-conditioned bots and 0 for Republican-conditioned bots, *X*_*ib*_ is a vector of covariates, *α* is the intercept, *β* is the coefficient on the Democratic conditioning indicator (the parameter of primary interest), *γ*′ is a vector of coefficients on the covariates *X*_*ib*_, *δ*_*b*_ are bot fixed effects, *λ*_*w*_ are week fixed effects and *ε*_*ib*_ is the error term. The covariate vector *X*_*ib*_ includes the number of videos watched by the bot during its experimental run, the proportion of recommended videos with transcripts, and a set of video- and channel-level engagement measures for both conditioning-stage and recommendation-stage videos (for example, likes, comments, plays, share count, follower count and verification status). We estimate the model using OLS with two-way cluster-robust standard errors by bot and week to account for within-bot and within-week dependence in recommendations.

Because many engagement covariates are highly correlated, we assess multicollinearity using variance inflation factors (VIFs). Independent variables with VIF values greater than five are combined into a lower-dimensional index using PCA; in particular, we summarize conditioning-stage video- and channel-level engagement variables into a single combined engagement score used as a control in the regression. Supplementary Tables 20 and 21 report the VIF scores for all covariates.

For ease of interpretation, the main-text regression figures report results from LPMs, whose coefficients can be read directly as percentage-point changes in the outcome (Supplementary Tables 22, 24 and 26). To assess robustness to functional-form assumptions, we also estimate the same specifications using logistic regression and report these results in Supplementary Tables 23, 25 and 27. Across all models, the sign and substantive significance of the key coefficients are largely similar in the logit and LPM specifications.

Given the large number of coefficients tested across related models, we adjust for multiple comparisons using Benjamini–Hochberg (BH) corrections, which control the false discovery rate. We report both unadjusted and BH-adjusted *P* values in Supplementary Tables 22–27, and use BH-adjusted *P* values as the basis for statistical significance markers in Fig. 3e and Supplementary Fig. 1b.

### Sensitivity analysis

Although we have shown evidence that observable engagement metrics and viewership patterns cannot explain the ideological skew in Republican-aligned content on TikTok, we cannot rule out the possibility that there are unobserved or latent engagement metrics or viewership patterns, known to the TikTok algorithm but unknown to us, that would explain the gap we observe. For instance, a key engagement metric such as ‘dwell time’, or how long a user spends watching a particular video, is not a publicly available metric published by TikTok. As such, we conduct a sensitivity analysis to calculate how strong such an unobserved factor would have to be to fully explain the apparent ideological skew of TikTok.

In Supplementary Table 39, we show in the first column the true difference between the Republican and Democratic videos with regard to their normalized values for a given engagement metric. In the remaining four columns, we show the ratio between the difference needed to match the observed skew and that of the true difference in mean between Republican and Democratic videos in a given engagement metric. As can be seen, for most of the engagement metrics, it is Democratic videos that tend to have higher engagement on average, with the exceptions being in the video comment count, such as count, play count, share count and composite score. Although the combination of these metrics produces the largest normalized Republican skew in engagement gap, this gap would need to be 9.4 times as large to fully explain the observed skew in recommendation rates seen in our experiments.

### Data representativeness

Given that our primary analysis throughout this study focuses on videos with transcripts, we must ensure that this sample is representative of videos throughout the entire dataset. To do so, we annotate a random sample of videos recommended to each of the sets of Republicans and Democrats during the experiment. Specifically, for each of these sets, we analyse a random sample of 2,000 videos that do not include transcripts to measure the following features of a given video: (1) whether the video is political in nature; (2) whether the video is concerned with the 2024 US elections or major US political figures; and (3) the ideological stance of the video in question (Pro Democrat, Anti Democrat, Pro Republican, Anti Republican or Neutral). This annotation task was again completed by three political science undergraduates, with high agreement between annotators (Krippendorf’s *α*; Q1: *α* = 0.858, Q2: *α* = 1.0, Q3: *α* = 0.989). We find that the inter-rater agreement when viewing the videos directly, rather than relying on the transcript alone (the results of which are in Supplementary Table 7), yielded higher agreement between the raters.

This process allows us to compare the distribution of political content in videos with transcripts against those without transcripts. The results of this comparison are shown in Extended Data Fig. 2. As can be seen, videos with no transcripts contained significantly fewer political videos on average relative to those that did include transcripts. This result is perhaps unsurprising given that videos that do not include a transcript are also less likely to include dialogue (for example, videos of landscapes or pets). However, of those that were political, we do not find significant differences (computed through *χ*^2^ tests) between transcript and non-transcript videos with regard to the proportion of political videos about the election, nor their distribution of Democrat-aligned, Republican-aligned or Neutral videos.

### Topic analysis

To identify the topics discussed in each political video, we use the methodology used in ref. ^54^. Specifically, the authors used GPT-4 to classify a video in one of a set of topics, and thoroughly validated their approach, finding accuracies greater than 0.9 for all topics.

We slightly modify the prompt used in ref. ^54^ to allow the LLM to select more than one topic option. The prompt used can be seen below:

You are an AI assistant trained to look at social media posts and determine what the post is about.

You will receive the transcript of the video in question.

You will be given a list of topics. Please tell us what this post is about.

Transcript: [TRANSCRIPT]

Some other things to keep in mind:

- There is an election in November, so many posts will be about that. The Democratic candidates were Joe Biden and Kamala Harris, but Joe Biden dropped out and it’s Kamala Harris and Tim Walz. The Republican candidates are Donald Trump and JD Vance. The independent candidates are Robert F Kennedy Jr (RFK Jr), Cornel West, and Jill Stein.
- If there are multiple topics, pick all the relevant topics.
- If a post seems political, first see if it goes into another category. For example a post about politics and race would go into the race category. Fall back on politics if none other fits, as long as there is something political.
- With the exception of immigration, most posts that reference a foreign country will go into the last category.

Categories:

1. Crime
  1.1. Crime generally
2. Environment
  2.1. Climate change
  2.2. Other environmental issues
3. Immigration
  3.1. Immigration generally
4. Social issues
  4.1. Abortion and reproductive health
  4.2. Guns and gun control
  4.3. LGBTQ+ issues, including transgender issues
  4.4. Racial issues, including affirmative action and racial discrimination
  4.5. Education
  4.6. Other social issues, including culture war issues, labor, and other social issues that are not covered above
5. Public health
  5.1. Covid, including covid vaccines
  5.2. Other vaccines
  5.3. Other public health issues
6. Economy
  6.1. Economy generally
7. Technology
  7.1. AI, LLMs
  7.2. Crypto
  7.3. Other technology issues
8. Government, politics and elections
  8.1. Assassination attempt on Donald Trump
  8.2. Republican National Convention (RNC)
  8.3. Democratic National Convention (DNC)
  8.4. Biden dropping out of the presidential race
  8.5. Other political or government related posts that do not fit into other categories
9. International issues
  9.1. Israel, Gaza or Palestine, including anything about Netanyahu or Hamas
  9.2. Ukraine war
  9.3. Anything outside the US or involve US foreign relations except for Israel, Gaza, Ukraine, or immigration
10. No topic
  10.1. None of the above topics

Using the above prompt, the topics discussed in the 7,767 ideologically stanced TikTok videos in our dataset were identified. Supplementary Table 40 describes the number of videos falling under each category, split by the ideological stance of the video.

### Video recommendation counterfactual models

To verify that the Republican skew observed in our experiments is not simply due to differences in the engagement metrics of Republican- and Democratic-aligned videos or channels, we build a series of counterfactual models to predict expected video recommendations based on these engagement metrics. Specifically, we aim to compute the ideological content that would arise if recommendations were driven purely by observable engagement, and then compare these expected skews to the ideological skew we actually observe in the feeds of the bots. As in the main text, we define the ideological content of a set of recommendations as the proportion of Republican-aligned videos minus the proportion of Democratic-aligned videos.

The metrics of interest include several video-level metrics, such as the number of plays or views a video receives, as well as its number of shares, comments and likes. Apart from video-level metrics, we also collect channel-level metrics such as the cumulative number of likes, followers and videos of a channel, as well as whether the channel is verified or not. Given that channel verification status is a binary attribute (True or False), we apply a value of 0.1 to channels that are not verified, and 0.9 to channels that are verified. For robustness, we also test all values between 0.5 and 1.0 in increments of 0.05 for verified channels (and inversely, 0.5 to 0 for unverified channels), and find similar results (Supplementary Table 17).

To retrieve predicted recommendation rates, for each week throughout the duration of the experiment we first isolate the set of videos seen by the bots during and before the week in question. We then compute normalized values of the metric in question using min–max normalization. Next, we compute a bootstrapped measure of expected ideological content by sampling *n* videos (where *n* is equivalent to the number of videos seen by a bot during a given week), weighted by the normalized metric in question. From each such sample, we compute the proportion of Republican-aligned and Democratic-aligned videos and take the difference to obtain the counterfactual ideological content for that draw. This allows us to compare the ideological content of the sampled recommendations to the ideological content actually observed in the feeds of the bots for the same state–week–condition cells. The sampling process is repeated 100 times for robustness to retrieve the mean and standard error of the expected ideological content per week for each set of partisan bots.

Apart from the single-value metrics mentioned above, we compute three additional engagement indices. The first is a linear combination of the normalized play, share, like and comment counts of a video. The second is a linear combination of the cumulative likes, followers and videos of a channel, as well as its verification status. The third is a linear combination of all video- and channel-level metrics. For each of these additional metrics, the weight of each component is derived using PCA, which identifies the linear combination of components that captures the maximum variance in the data. We use the loadings (coefficients) from the first principal component as weights, normalizing them to sum to 1, and repeat the sampling process described above for each of these three combined metrics.

The models that consider the number of likes or comments disregard the fact that these metrics are not independent of the recommendation algorithm. Videos that receive more recommendations end up being viewed more, which in turn increases their likelihood of receiving likes and/or comments. Thus, to develop counterfactual models that capture the intrinsic ‘quality’ of a video while partially isolating the effect of the algorithm, we normalize these metrics as follows. First, we take the number of ‘recommendations’ a video receives as the number of plays minus the number of shares. This subtraction yields the number of views received by the video, specifically by the recommendation algorithm, and not by users sharing the video. With this recommendations metric, we additionally develop counterfactual models that account for the number of likes or comments per recommendation of a video.

Finally, for each of the aforementioned metrics, we repeat the sampling processes while taking into account the potential favourability of recent content. Specifically, we inversely scale each metric in question with the time between when an audit was conducted and the publishing date of a given video, once linearly and once exponentially. In total, this amounts to 39 different counterfactual models with which we compare observed ideological content and expected ideological content. In Supplementary Fig. 1a, the leftmost point represents the observed ideological skew in the recommendations of the bots (the proportion of Republican-aligned videos minus the proportion of Democratic-aligned ones). Each of the remaining points presents the expected ideological skew as computed by the counterfactual models, with point markers indicating alternative recency-weighting schemes. If the observed engagement metrics fully explained the partisan gap, we would expect these engagement-based counterfactual skews to be close to the observed skew of approximately 0.2. Instead, across all 39 counterfactuals, the observed skew towards Republican-aligned content substantially exceeds the counterfactual skews, and many engagement-based models imply a Democratic-leaning skew. Supplementary Table 14 details the ideological skew computed by each model, as well as *t*-test results comparing the observed and expected skews. Supplementary Tables 15 and 16 report analogous results for models using only positive or only negative partisan videos, respectively.

### Survey

We administered a preregistered survey with a sample of 1,008 US-based TikTok users to assess whether individuals had noticed changes to the content of their TikTok feeds, particularly political content, over the past year. The survey was preregistered on OSF (https://osf.io/udywb/) and was deemed exempt by the institutional review board of the authors (IRB protocol no. HRPP-2025-69).

Participants were recruited using the online platform Prolific and screened to ensure they resided in the USA and were active users of TikTok. The survey consisted of two parts: (1) a series of open-ended text-entry questions; and (2) a series of structured, scale-based questions. Open-ended items asked participants whether they had noticed any changes to the content on their TikTok feed in general, any changes to political content specifically and whether the tone of political content had become more positive or negative. Responses to these questions were manually coded by the first author to determine whether participants explicitly referenced changes to political content and, if so, whether they described seeing more Republican-aligned or Democratic-aligned content.

Structured questions asked participants to rate, on a 0–10 scale, the extent to which their feed had shifted towards Democratic or Republican content, become more positive or negative in tone, or featured more political content they agreed or disagreed with.

For each survey item, we conducted separate linear regression analyses that included participant political affiliation and demographic covariates (age, gender, race and education level) as predictors. Full regression results are presented in Supplementary Tables 33 and 34 for the open-ended and structured questions, respectively. Participant demographic characteristics are summarized in Supplementary Table 35, and the complete survey instrument is available in Supplementary Note 6.

### Reporting summary

Further information on research design is available in the Nature Portfolio Reporting Summary linked to this article.

## Online content

Any methods, additional references, Nature Portfolio reporting summaries, source data, extended data, supplementary information, acknowledgements, peer review information; details of author contributions and competing interests; and statements of data and code availability are available at 10.1038/s41586-026-10447-1.

## Supplementary information

Supplementary Information This file contains Supplementary Notes 1–6, Supplementary Tables 1–40, Supplementary Figs. 1–12 and additional references.

Reporting Summary

Peer Review File

## Data Availability

The full dataset of political videos on TikTok analysed in this study can be found in ref. ^55^.

## Code availability

All data analysis was conducted using Python 3.13.1. All codes needed to replicate the findings can be found in ref. ^55^.
