# Supplementary material for: Systematic partisan content skews in TikTok during the 2024 US elections
Source: Nature. 2026 May 6;654(8120):1004–11. doi: 10.1038/s41586-026-10447-1 (PMC13293873; doi:10.1038/s41586-026-10447-1)
Supplement: Supplementary file 1 — This file contains Supplementary Notes 1–6, Supplementary Tables 1–40, Supplementary Figs. 1–12 and additional references. [file 41586_2026_10447_MOESM1_ESM.pdf]

nature portfolio

https://doi.org/10.1038/s41586-026-10447-1

Supplementary information

Systematic partisan content skews in TikTok during the 2024 US elections

In the format provided by the authors and unedited

Supplementary Information for "Systematic partisan content skews in TikTok during the 2024 U.S. elections"

Hazem Ibrahim[1+], HyunSeok Daniel Jang[1+], Nouar Aldahoul[1],
Aaron R. Kaufman[2], Talal Rahwan[1*], and Yasir Zaki[1*]

[1]Computer Science, New York University Abu Dhabi, UAE.
[2]Political Science, New York University Abu Dhabi, UAE.
[+]Joint first author
[*]Corresponding authors. E-mail: {talal.rahwan,yasir.zaki}@nyu.edu

This document is structured as follows:

- **Supplementary Note 1: Background on TikTok's geopolitical context** (*page 2*)
- **Supplementary Note 2: Partisan Presence on TikTok** (*page 3*)
- **Supplementary Note 3: Robustness checks** (*page 5*)
- **Supplementary Note 4: Top Democratic and Republican channels** (*page 7*)
- **Supplementary Note 5: Topic Analysis** (*page 9*)
- **Supplementary Note 6: Survey Materials** (*page 11*)
- **Supplementary Tables 2-40** (*page 16*)
- **Supplementary Figures 4-12** (*page 45*)

Supplementary Note 1: Background on TikTok's geopolitical context

TikTok, a social media platform owned by Chinese company ByteDance, has rapidly grown to more than a billion monthly active users worldwide [1], establishing itself as a major player in the social media space, particularly among younger demographics. In the United States alone, TikTok supports over 170 million monthly active users [2], with one-third of U.S. adults—and a majority of those under 30—using the platform regularly [3]. Due to its scale, the platform has emerged as a significant source for news content, with 39% of adults under 30 and 19% of those aged 30-49 reporting that they regularly get news from the app [4]. This shift in how Americans consume news has raised concerns about the platform's potential to shape political narratives and influence the democratic process.

Despite TikTok's policy prohibiting political advertising [5], the platform's role in elections has been widely acknowledged and highly scrutinized in the media. For example, TikTok may have played a pivotal role in the 2024 Romanian presidential election: researchers identified thousands of bot-like accounts engaging in coordinated activity to amplify the campaign of a surprise Pro-Russia candidate, Călin Georgescu [6]. In response, Romania's Prime Minister called for an investigation into the candidate's TikTok campaign funding and a national regulator suggested suspending TikTok for election interference [7]. The European Parliament has since summoned TikTok's CEO to address these allegations, with some lawmakers questioning whether the platform violated the EU's Digital Services Act [8] by failing to curb the spread of disinformation and inauthentic behavior [6]. In the U.S., concerns about TikTok's influence are compounded by national security owing to its Chinese ownership. A 2020 executive order by Donald Trump sought to ban the app unless it was sold to a U.S. company [9], though the order was ultimately blocked by a federal judge [10] and Trump later reversed his position during to the 2024 U.S. election, advocating against the banning of TikTok [11]. Nevertheless, bipartisan support for a potential TikTok ban has resurfaced, with a Senate-approved bill requiring the app to either divest from its Chinese parent company or face a ban in the United States [12]. The bill, which passed with a vote of 360-58 in April 2024, exists in a broader geopolitical conflict between the U.S. and China. Some arguments supporting the ban emphasize TikTok as a path for Chinese political influence on the U.S., especially considering the Chinese government's heavy hand in shaping corporate strategy within Chinese-owned companies [13, 14]. Moreover, TikTok has a history of politically-motivated censorship: TikTok's internal moderation guidelines, covered by The Guardian in 2019 [15], suggest that the company censored Chinese politically-sensitive topics such as Tiananmen Square or Tibetan independence, and TikTok publicly apologized after a glitch that censored hashtags relating to the Black Lives Matter movement in the wake of George Floyd's death in 2020 [16]. As of January 20th, 2025, after a brief interruption to TikTok's services, the platform resumed activity in the United States [17].

Supplementary Note 2: Partisan Presence on TikTok

What is the supply and partisan distribution of political content creators on TikTok? To categorize channels as Democratic-aligned or Republican-aligned, we calculate the proportion of each creator's videos labeled as Pro-Democratic or Anti-Republican versus Pro-Republican or Anti-Democratic, supplementing our dataset with up to 30 additional pre-election videos from the TikAPI [18] for channels with fewer than 10 labeled videos in our sample. These additional videos were then passed through the same pipeline described in the previous section to discern their partisan alignment. We label a channel as Democratic-aligned if at least 75% of its videos are either Pro-Democratic or Anti-Republican, and Republican-aligned if at least 75% of its videos are Pro-Republican or Anti-Democrat. This process yielded 56 Democratic-aligned channels and 75 Republican-aligned channels, which we manually validated following best practices on channel-level classification tasks [19, 20]. We adopt this relatively high threshold to ensure that classified channels are consistently partisan in their output, recognizing that many creators intersperse political content with non-political or more neutral material for many reasons such as monetization or audience growth. A stricter cutoff reduces the risk of misclassifying mixed-content creators as partisan based on a small subset of videos. Table 38 in the Methods section summarizes the average proportion of party-aligned videos across these channels.

Table 1 details the 20 largest channels classified as either Democratic-aligned or Republican-aligned ranked by follower count, including cumulative engagement metrics such as the total likes received and the number of videos published. The left columns show that the top 20 Democratic channels in our sample are dominated by known political figures (e.g., Kamala Harris, Tim Walz, Bernie Sanders, and Alexandria Ocasio-Cortez), talk shows (e.g., The View, Jimmy Kimmel Live, and The Colbert Late Show), and news-media outlets (e.g., New York Times, MSNBC, and Courier News Room). In contrast, the top Republican-aligned channels in the right columns of Table 1 feature more independent "influencer" channels (e.g., Adam Calhoun, Charlie Kirk, Ben Shapiro, Brandon Tatum, and Patrick Bet-David). The bottom of Table 1 summarizes engagement metrics for the top 50 accounts in both partisan categories. Republican channels saw higher median engagement across followers and likes per channel compared to their Democratic-aligned counterparts, but had a smaller number of likes overall, due to having far fewer videos published on the platform. The interquartile range (IQR), reported as the range between the 25th and 75th percentile, showed greater variability among Republican channels for followers but greater variability among Democratic channels for likes, suggesting that top Republican channels generally received more consistent engagement rates over their Democratic counterparts.

As of the writing of this manuscript, no public, platform-wide census of partisan TikTok creators currently exists. Consequently, our validated list of 131 partisan channels constitutes a secondary contribution of this study. The only proximate reference point is a 2024 Pew Research Center report [21], which finds that just 25% of high-reach TikTok creators self-identify as Republican

and 28% as Democratic. Pew's lower partisan shares likely reflect its stricter inclusion rule, as they require an explicit self-declaration of party support, whereas our classification relies on the ideological orientation of the channel's content, a broader criterion that captures politically active accounts even when creators do not state an affiliation.

Account name (ID)	Number of followers	Number of likes	Number of videos	Account name (ID)	Number of followers	Number of likes	Number of videos
Kamala Harris (kamalaharris)	9.3M	149.5M	219	President Donald J Trump (realdonaldtrump)	14.7M	107.2M	58
TizzyEnt (tizzyent)	6.7M	270.9M	2.6K	Team Trump (teamtrump)	8.3M	204.3M	447
Kamala HQ (kamalahq)	5.7M	308.9M	1.1K	The Charlie Kirk Show (thecharliekirkshow)	5.3M	134.7M	638
MSNBC (msnbc)	4.1M	265.9M	4.3K	Date Right Stuff (daterightstuff)	3.4M	197.6M	787
NowThis Impact (nowthisimpact)	4.0M	257.7M	2.9K	Candace Owens Show (candaceoshow)	3.3M	35.4M	342
The View (theviewabc)	3.7M	37.7M	1.8K	Robert F. Kennedy Jr (robertfkennedyjrofficial)	3.2M	63.7M	886
Late Show With Stephen Colbert (colbertlateshow)	2.2M	113.3M	1.4K	Adam Calhoun (adamcalhoun1)	3M	36.1M	153
Jeff Jackson (jeffjacksonnc)	2.2M	38.7M	121	Ben Shapiro (real.benshapiro)	2.7M	59.9M	1056
Tim Walz (timwalz)	2.1M	22.0M	104	J.D. Vance (jd)	2.3M	12.3M	39
Jimmy Kimmel Live (jimmykimmellive)	2.0M	76.2M	587	Piers Morgan Uncensored (piersmorganuncensored)	2.2M	32M	630
Bernie Sanders (bernie)	1.6M	14.4M	331	Tucker Carlson (tuckercarlson)	2.2M	26.2M	224
Harry Sisson (harryjsisson)	1.5M	136.2M	3.7K	Fox News (foxnews)	1.9M	55.5M	1138
The Democrats (thedemocrats)	1.4M	56.7M	1.3K	The Comments Section (thecommentssectiondw)	1.9M	65.6M	999
Courier Newsroom (couriernewsroom)	1.3M	156.4M	3.8K	Jeff Mead (the_jefferymead)	1.9M	45.7M	1.5K
Aaron Parnas (aaronparnas1)	1.3M	70.1M	2.7K	Donald Trump Jr. (donaldjtrumpjr)	1.7M	14.4M	166
The New York Times (nytimes)	1.3M	31.6M	1.5K	Charlie Kirk (charliekirkdebateclips)	1.6M	17.5M	147
MeidasTouch (meidastouch)	1.2M	91.6M	3.3K	Make America Great Again (maga)	1.4M	44.7M	408
Alexandria Ocasio-Cortez (aocinthehouse)	1M	7.5M	74	The Officer Tatum (theofficertatum)	1.4M	41.6M	671
Robert Reich (rbreich)	938.8K	17.8M	1.0K	Jesse Watters (jessebwatters)	1.3M	36.2M	1.3K
Late Night with Seth Meyers (latenightseth)	887.9K	72.8M	1.3K	Patrick Bet-David (patrickbetdavid)	1.3M	27.5M	3.4K
**Top 50 Accounts**							
Sum	66.8M	2.6B	83703	Sum	87.3M	1.78B	48642
Median	709K	17.9M	1038	Median	1.2M	18.5M	794
IQR	1M	61.5M	2180.5	IQR	1.3M	33.9M	739

Table 1: Top 20 Democrat-aligned channels (left) and Republican-aligned channels (right) by follower count, and the cumulative engagement metrics for the 50 Democrat and Republican-aligned channels with the largest number of followers (bottom).

Supplementary Note 3: Robustness checks

Main Figure 2 shows that TikTok recommends more Republican-aligned than Democratic-aligned content overall, and that Democratic-conditioned bots receive more Republican-aligned content than Republican-conditioned bots receive Democratic-aligned content. Before attributing these asymmetries to TikTok's internal algorithmic rules, we ask whether they could instead be explained by user-centric mechanisms: the overall supply of Republican- versus Democratic-aligned content, differences in engagement with that content, or asymmetric cross-party interest (for example, Democrats engaging more with Republican content than vice versa).

We first construct a series of counterfactual models in which recommendations are driven solely by observed engagement metrics for videos and channels (e.g., likes, comments, plays, follower counts, and aggregated engagement indices; see Methods, Video recommendation counterfactual models). Across 48 such counterfactuals, the ideological skew that would be expected if TikTok simply recommended content in proportion to these engagement measures is consistently smaller than the skew we observe, and in many cases would actually predict a Democratic-leaning skew; see Supplementary Figure 1A and Supplementary Table 14.

Second, we examine whether asymmetric homophily in co-viewership networks can account for the results [22]. If Democrats engaged more with Republican-aligned videos than Republicans engaged with Democratic-aligned videos, we would expect Republican-aligned videos with a higher share of Democratic comments to be shown more often to Democratic bots. Using a random sample of comments from political videos, we classify the partisan alignment of each comment relative to the video and compute the composition of comments by video type (see Methods, Comment classification and asymmetric homophily). We find that Democratic-aligned videos actually attract more Republican-aligned comments and more copartisan comments than Republican-aligned videos, while Republican-aligned videos receive more neutral comments. Incorporating comment composition into our engagement-based counterfactuals yields similar conclusions (Supplementary Figure 1A). These patterns provide no evidence of asymmetric homophily that could generate the observed Republican-leaning skew.

Third, we formalize these analyses in a linear probability model at the bot–video level, where the outcome indicates whether a recommended video is a cross-partisan recommendation and the key predictor is the bot's conditioned partisanship, controlling for state, week, number of videos watched, transcript availability, and a range of video- and channel-level engagement metrics from both the conditioning and recommendation stages (see Methods, Cross-partisan recommendation regression model). Even after accounting for these factors, Democrat-aligned bots are 30.8 percentage points more likely to receive cross-partisan recommendations than Republican-aligned bots, on average (Supplementary Figure 1B; Supplementary Table 22). Finally, a sensitivity analysis shows that any unobserved engagement signal would need to differ between Republican- and Democratic-aligned videos by a factor roughly 98 times larger than the observed gap in likes to fully explain

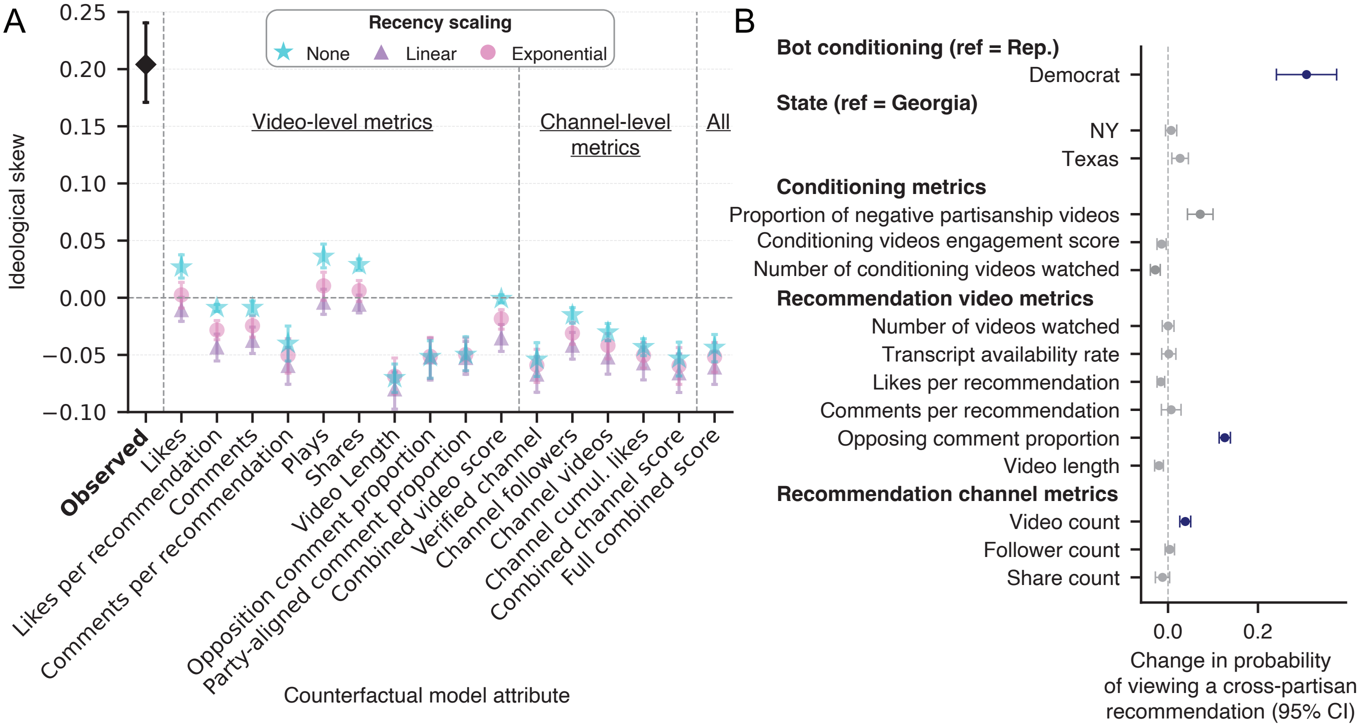

Figure 1: **Robustness checks.** (**A**) The observed ideological skew, and the expected ideological skew based on counterfactual models built on different engagement metrics. N = 1002 simulations across 17 counterfactual models and 3 scaling factors.(**B**) Linear probability model estimates on the percentage point change in the probability of viewing a cross-partisan recommendation. N = 3,539 videos. Statistical significance is measured using t-tests. Statistically significant coefficients after Benjamini-Hochberg corrections are highlighted in navy. Unadjusted p-values, and p-values after Benjamini-Hochberg multiple comparison corrections can be found in Supplementary Table 22. Standard errors are clustered by bot id and week in which experiment is conducted.

the ideological content gap we document (Methods, Sensitivity analysis). Taken together, these analyses suggest that the Republican-leaning skew in TikTok's political recommendations cannot be fully accounted for by observable user behaviour on the platform.

Supplementary Note 4: Top Democratic and Republican channels

Here we examine cross-partisan recommendations at the channel-level. We compute a channel's cross-partisan recommendations as the proportion of that channel's videos shown to bots conditioned with videos aligned with the opposing party. Here, we focus on the top Democratic and Republican channels by follower count who were watched at least 10 times by bots in each state. The proportion of cross-partisan recommendations for these channels are illustrated in Supplementary Figure 2A. Videos published by Donald Trump's official TikTok channel (`realdonaldtrump`) had an average proportion of 0.269, meaning that nearly 27% of the time his videos were recommended to our bots, they were recommended to Democratic-conditioned bots. In contrast, Kamala Harris's average proportion was only 0.153, despite being the sitting Vice President during our experiment.

Indeed, of the top Republican and Democratic channels, the highest four cross-partisan recommendation proportions were of Republican channels (`daterightstuff`, `foxnews`, `realdonaldtrump`, and `teamtrump`). In contrast, four channels were never shown to a bot of an opposite ideological alignment during our experiments (`real.benshapiro`, `rbreich`, `jeffjacksonnc`, and `timwalz`). Overall, Republican channels had a significantly higher proportion than Democratic channels (Chi-squared test; $\chi^2 = 36.5$, $p < 0.001$). Supplementary Figure 2B shows that the official channels of the Republican candidates, Donald Trump and JD Vance, had a significantly higher proportion than those of Democratic candidates Kamala Harris and Tim Walz (Chi-squared test; $\chi^2 = 28.1$, $p < 0.001$). To test whether it is specifically cross-partisan recommendations within top accounts that contribute to the skews observed overall, we compute a linear probability model similar to that shown in Supplementary Figure 1B, while including another binary channel-level metric designating whether a channel is amongst one of the top 20 channels by follower count. The results of this analysis are in Supplementary Figure 2C. Here, we again find that the partisan discrepancy observed is most strongly associated with the bot's partisan conditioning, as well as the opposing comment proportion of a given video. In contrast, we find that top channels were less likely to be recommended to a bot of an opposing ideological alignment, despite the discrepancies seen in Supplementary Figure 2A and 2B, suggesting that less popular channels were even more likely to be recommended to a bot of an opposing alignment. We again considered variables with a VIF value less than 5, as listed in Supplementary Table 21. See Supplementary Table 24 for the regression table containing these results with both adjusted and unadjusted p-values.

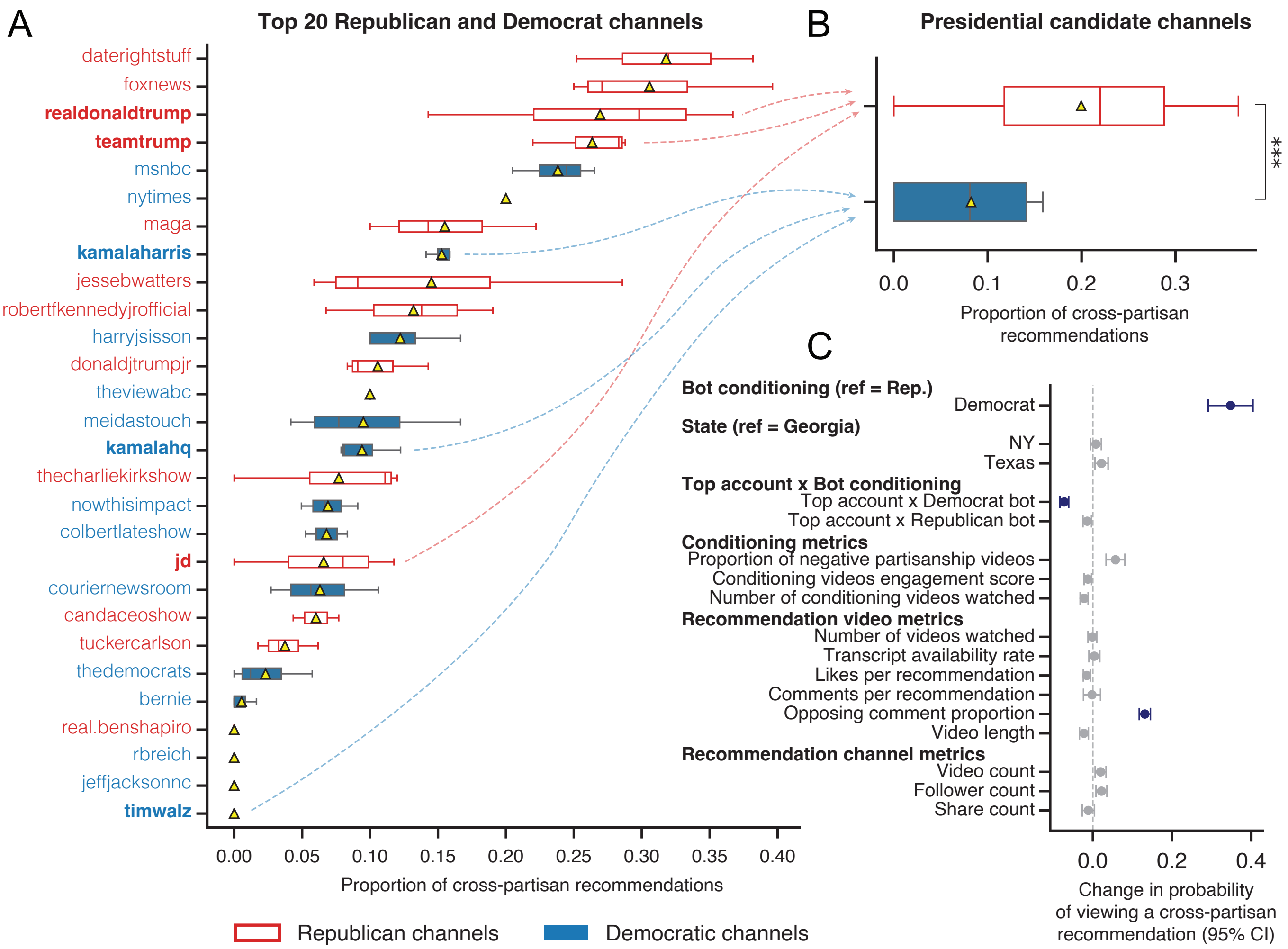

Figure 2: **Cross-partisan recommendation rates of videos by top Democratic and Republican channels.** (**A**) The proportion of cross-partisan recommendations for videos published by top Democratic and Republican TikTok channels by follower count. N = 75 across 28 channels. (**B**) The proportion of cross-partisan recommendations for videos published by the TikTok accounts of the main political figures in the 2024 U.S. elections (Trump, JD Vance, Kamala Harris, and Tim Walz). N = 18 across 6 channels. In both A and B, box bounds indicate Q1 and Q3 quartile values, while minima and maxima are shown with whiskers. The median and mean values are illustrated with a line and a triangle, respectively. (**C**) Linear probability model estimates on the percentage point change in the probability of viewing a cross-partisan recommendation when accounting for top account status. Statistical significance is measured using t-tests. N = 3,539 videos. Statistically significant coefficients after Benjamini-Hochberg corrections are highlighted in navy. Unadjusted p-values, and p-values after Benjamini-Hochberg multiple comparison corrections can be found in Supplementary Table 24. Standard errors are clustered by bot id and week in which experiment is conducted. Error bars denote 95% confidence intervals.

Supplementary Note 5: Topic Analysis

What do partisan videos discuss, and are the asymmetries we observe concentrated in particular policy domains rather than spread evenly across political content? To address this, we use methods from [23] to identify the substantive topics covered in each political video (see Topic Analysis in the Methods). This allows us to assess whether mismatched recommendations arise across the board or are driven by imbalances in specific issue areas—an important distinction for understanding which domains platform algorithms are most likely to polarize. We isolate topics appearing in at least 100 unique videos and compute the difference between the proportion of Republican- and Democratic-aligned videos for each topic. Figure 3A plots these differences, ordered from most proportionally Republican to most proportionally Democratic. Topics stereotypically associated with Democrats (e.g., climate change, abortion and reproductive health) show greater Democratic coverage, whereas immigration, foreign policy, and the Ukraine war are more heavily covered by Republican-aligned videos. Supplementary Table 19 reports full topic distributions and chi-squared comparisons.

Having established that Democratic-conditioned bots viewed significantly more cross-partisan videos in previous sections, next, we analyze what topics such videos discussed. To this end, we focus on topics with a given partisan stance (Democratic- or Republican-aligned) that were viewed at least 100 times. For each such topic, Figure 3B illustrates the proportion of watches by Democratic-conditioned bots that were Democratic-aligned (blue) and Republican-aligned (red). Similarly, Figure 3C illustrates these proportions but for Republican-conditioned bots. Videos seen by Democratic-conditioned bots on the topics of immigration, crime, the Gaza conflict, or foreign policy broadly, were most likely to be of an opposing ideological alignment, with cross-partisan recommendations representing 56.9% and 58.9% of the latter two topics. In contrast, videos on the topics of abortion and reproductive health were proportionally the most common cross-partisan recommendations for Republican-conditioned bots, although these only account for 25.0% of all abortion-related videos seen by Republican bots. Supplementary Figures 12A and 12B mirror Supplementary Figures 3B and 3C while including videos classified as “Neutral” as well. Supplementary Figure 11 illustrates the difference in the proportion of videos on a topic with a given stance and the proportion of videos of that stance out of all political videos. Together, these results show that cross-partisan exposure on TikTok is concentrated in a narrow set of contentious policy domains rather than spread uniformly across political content. This topic-specific structure implies that any algorithmically driven ideological polarization may become most pronounced on issues like immigration, foreign policy, and abortion, where recommendation asymmetries are greatest.

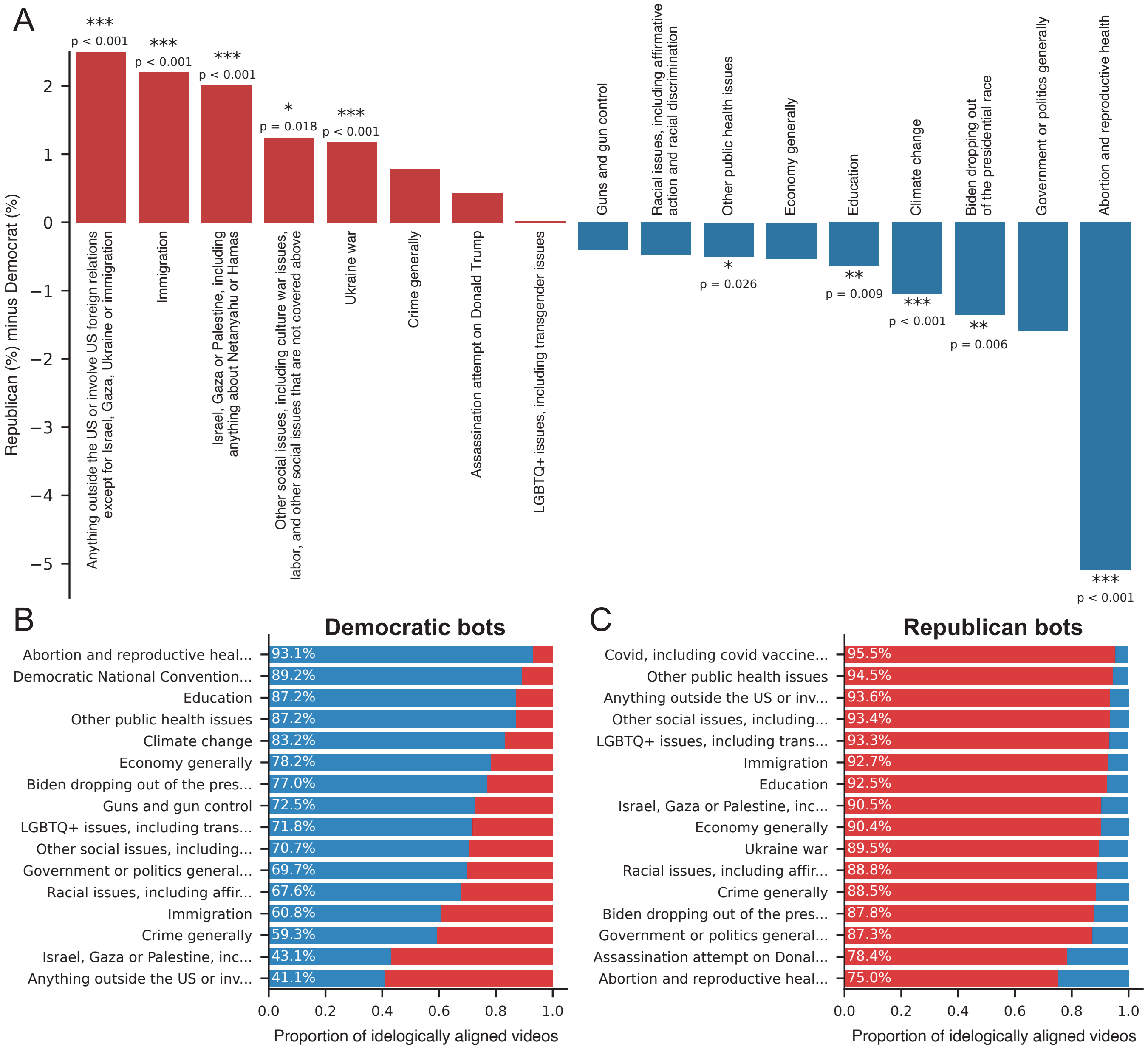

Figure 3: **Topic analysis of political TikTok videos.** (**A**) The percentage of Republican-aligned videos on a given topic minus the percentage of Democratic-aligned videos on that topic. Statistically significant difference between Republican and Democratic percentages are highlighted with stars (Two-sided chi-squared tests; $* : p < 0.05$, $** : p < 0.01$, $*** : p < 0.001$). (**B**, **C**) The proportion of videos on a given topic which are ideologically-aligned and ideologically-opposing seen by Democratic and Republican-conditioned bots, respectively. For each plot, topics are listed in descending order of ideological-alignment.

Supplementary Note 6: Survey Materials

Informed Consent

You are being asked to provide consent to participate in a research study. Participation is voluntary. You can say yes or no. If you say yes now, you can still change your mind later.

Purpose of Research: This research is being conducted to better understand changes in your TikTok feed over the past 12 months.

Procedures: You will be asked a series of open-ended and multiple-choice questions. **Please do not use ChatGPT or any large language model to generate answers. If use is detected, you will NOT be compensated for your participation.**

Duration: Participation will involve approximately 6 minutes of your time.

Risks: We believe there are no known risks associated with this research study. Your participation is voluntary, and you may withdraw at any time without penalty or loss of benefits.

Benefits: We aim to understand TikTok's recommendation algorithm in the context of U.S. politics, which may be beneficial in the future.

Compensation: You will be compensated approximately $1.15 for your time.

Privacy and Confidentiality: Study records will be kept in a secure location. Electronic files will be password-protected. Only the research team will have access. Shared data will be anonymized. Results may be published in summary form without identifying information.

The NYU Abu Dhabi Institutional Review Board (IRB) may inspect study records, but these reviews will only focus on researchers, not participants.

If you have questions about this study, contact the principal investigator, Yasir Zaki (yasir.zaki@nyu.edu). For questions about your rights as a participant, contact the IRB at irbnyuad@nyu.edu.

Consent Acknowledgment:

1. Your participation is voluntary.
2. You are 18 years of age or older.
3. You may withdraw at any time.

Options:

- I consent, begin the study.
- I do not consent, I do not wish to participate.

Survey Questions

Free Response Questions

Q1) Over the past 12 months, from March 2024 to today, how has the **overall content** of your TikTok feed changed?

[Text Entry]

Q2) Over the past 12 months, from March 2024 to today, how has the **political content** of your TikTok feed changed?

[Text Entry]

Q3) Over the past 12 months, from March 2024 to today, has the **political content** of your TikTok feed become **more positive or more negative**?

[Text Entry]

Structured Scale Questions

Q4) Over the past 12 months, your TikTok feed has become:
0 (Less Political) — 10 (More Political)

Q5) Over the past 12 months, your TikTok feed has become:
0 (More political content you disagree with) — 10 (More political content you agree with)

Q6) Over the past 12 months, the **political content** in your TikTok feed has become:
0 (More Democratic) — 10 (More Republican)

Q7) Over the past 12 months, the **political content** in your TikTok feed has become:
0 (More Pro-Trump) — 10 (More Anti-Trump)

Q8) Over the past 12 months, the **political content** in your TikTok feed has become:
0 (More Positive) — 10 (More Negative)

Q9) Over the past 12 months, the **political content** in your TikTok feed has become:
0 (More Pessimistic) — 10 (More Optimistic)

Demographic Questions

Q10) How old are you?

- Under 18
- 18–24

- 25–34
- 35–44
- 45–54
- 55–64
- 65+

Q11) How do you describe yourself?

- Male
- Female
- Non-binary / third-gender
- Prefer to self-describe: *[Text Entry]*
- Prefer not to say

Q12) What racial or ethnic group best describes you?

- White
- Black
- Hispanic
- Asian
- Native American
- Middle Eastern
- Two or more races
- Other: *[Text Entry]*
- Prefer not to answer

Q13) What is the highest level of education you have completed?

- Some high school or less
- High school diploma or GED
- Some college, but no degree
- Associates or technical degree
- Bachelor's degree
- Graduate or professional degree
- Prefer not to say

Q14) What is your US Zip Code?
[Text Entry]

Q15) Which political party do you most identify with?

- Democratic Party
- Republican Party
- Independent
- Other: *[Text Entry]*

Q16) If Democratic Party (from Q15): Would you call yourself a:

- Strong Democrat
- Not so strong Democrat
- Prefer not to answer

Q17) If Republican Party (from Q15): Would you call yourself a:

- Strong Republican
- Not so strong Republican
- Prefer not to answer

Q16) Do you think of yourself as closer to the Democratic or the Republican party?

- Democratic Party
- Republican Party
- Neither
- Not sure
- Prefer not to answer

Q17) In general, how would you describe your own political viewpoint?

- Very liberal
- Liberal
- Moderate
- Conservative
- Very conservative
- Not sure

- Prefer not to answer

Q18) Did you vote in the 2024 presidential election?

- Yes
- No
- Prefer not to answer

Q19) What party did you vote for?

- Democrat
- Green
- Independent
- Libertarian
- Republican
- Other

Q20) Do you plan to vote in the 2026 midterm elections?

- Yes
- No
- Not sure
- Prefer not to answer

Q21) Which party do you plan to vote for?

- Democrat
- Green
- Independent
- Libertarian
- Republican
- Other

Q22) Please tell us anything else you would like to share about your experience on TikTok, politics, or this survey?

[Text Entry]

Supplementary Tables

	State		
Conditioning-Leaning	New York	Georgia	Texas
Democrat	3x weekly	3x weekly	3x weekly
Republican	3x weekly	3x weekly	3x weekly
Neutral		3x weekly	

Table 2: Summary of experimental conditions

**Week**	**Georgia**			**New York**		**Texas**	
	Democrat	Neutral	Republican	Democrat	Republican	Democrat	Republican
30-04-2024	1	1	1	1	1	1	1
07-05-2024	0	2	0	1	1	0	0
14-05-2024	1	2	1	2	2	2	2
21-05-2024	1	2	1	2	2	2	2
28-05-2024	0	2	0	1	1	2	2
04-06-2024	1	2	1	1	1	2	2
12-06-2024	0	2	0	1	1	2	2
18-06-2024	0	2	0	2	2	2	2
26-06-2024	1	2	1	2	2	2	2
03-07-2024	0	2	0	2	2	3	3
11-07-2024	2	2	2	2	2	3	3
18-07-2024	1	2	1	1	1	3	3
25-07-2024	2	2	2	2	2	0	0
01-08-2024	2	1	2	3	3	0	0
08-08-2024	2	2	2	3	3	0	0
15-08-2024	2	2	2	3	3	0	0
23-08-2024	2	2	2	2	2	0	0
30-08-2024	2	2	2	3	3	0	0
08-09-2024	1	2	1	1	1	0	0
16-09-2024	2	2	2	3	3	0	0
23-09-2024	3	2	3	3	3	3	3
30-09-2024	3	2	3	3	3	3	3
08-10-2024	3	2	3	3	3	3	3
15-10-2024	2	2	1	2	2	1	1
21-10-2024	3	2	3	3	3	3	3
28-10-2024	0	2	0	0	0	2	2
04-11-2024	3	2	3	2	2	3	3

Table 3: The number of successful experimental runs per condition per week.

**Week**	**Conditioning**		**Recommendation**	
	Total Videos	Unique Videos	Total Videos	Unique Videos
1	2140	807	5136	4581
2	542	535	4340	3760
3	3785	862	6168	4287
4	3747	841	9922	7181
5	2333	919	8550	6813
6	2916	839	10644	8209
7	2384	857	7850	6665
8	3013	862	9922	7979
9	3725	815	12997	9553
10	3667	816	11488	8296
11	4003	683	17682	11812
12	2485	680	13344	9632
13	2531	851	9858	7907
14	3400	849	10194	8001
15	3356	871	9924	7483
16	3643	830	12110	9182
17	3196	826	9838	7959
18	3801	848	13122	9537
19	1587	806	7363	6352
20	3488	907	11592	9651
21	5849	903	16246	12178
22	7058	941	21178	15623
23	6855	927	14168	9950
24	2808	850	3777	3263
25	4477	871	12739	10059
26	1205	764	3110	2896
27	3940	855	10831	8605

Table 4: Total and unique number of videos watched by week and experimental stage

Account name (ID)	Leaning	% Politically Leaning Videos
LiveNOW from FOX (livenowfox)	Republican	71.4
Al Jazeera English (aljazeeraenglish)	Republican	70.6
Channel 4 News (c4news)	Republican	70.0
New York Post News (nypost)	Republican	70.0
News Nation (newsnationnow)	Republican	70.0
Daily Mail (dailymail)	Republican	69.5
Don Lemon (donlemon)	Republican	61.5
Zeteo (zeteonews)	Democrat	64.7
The Problem With Jon Stewart (theproblem)	Democrat	66.7
Walter Masterson (waltermasterson)	Democrat	67.6
ashleembanks (ashleembanks)	Democrat	69.6
USA TODAY (usatoday)	Democrat	72.7
The Daily Show (thedailyshow)	Democrat	72.9

Table 5: TikTok accounts with 60-75% of their analyzed videos aligning with either the Republican or Democrat party

Question	Majority Vote	Count
Question 1: Given the following video transcript, do you think the topic is political?	No	31520
	Yes	8744
Question 2: Given the following video transcript, do you think the topic is related to the 2024 US election or related to Donald Trump, Kamala Harris, Joe Biden, JD Vance, or Tim Walz?	No	4392
	Yes	4229
Question 3: Given the following video transcript, classify the transcript into one of the following categories:	Neutral	3389
	Anti Democrat	1949
	Anti Republican	1177
	Pro Democrat	667
	Pro Republican	586

Table 6: The number of videos classified as political, pertaining to the U.S. presidential election, and of each ideological stance.

	Fleiss Kappa	Krippendorf's Alpha
Question 1	0.715	0.615
Question 2	0.696	0.626
Question 3	0.631	0.560

Table 7: Inter-LLM agreement metrics for the three prompts.

	Inter-Rater Reliability (Krippendorf's Alpha)	Accuracy	Cohen Kappa	F1 Score
Question 1	0.886	0.965	0.930	0.965
Question 2	0.851	0.970	0.940	0.970
Question 3	0.886	0.957	0.936	0.784

Table 8: Human validation accuracy and agreement metrics for LLM-ensemble classification tasks.

		**Question 1**	**Question 2**	**Question 3**
Accuracy	GPT 4o	0.710	0.965	0.854
	Gemini-Pro	0.915	0.955	0.890
	GPT 4	0.955	0.945	0.909
	Majority	0.965	0.970	0.957
Cohen Kappa	GPT 4o	0.410	0.930	0.767
	Gemini-Pro	0.831	0.910	0.838
	GPT 4	0.910	0.888	0.859
	Majority	0.930	0.940	0.936
F1 Score	GPT 4o	0.683	0.965	0.652
	Gemini-Pro	0.915	0.955	0.707
	GPT 4	0.955	0.944	0.728
	Majority	0.965	0.970	0.784

Table 9: The accuracy, Cohen kappa, and F1-scores of each LLM model individually, as well as the LLM majority vote, when compared with human ground truth labels.

	Inter-Rater Reliability Krippendorf's Alpha	Accuracy	Cohen Kappa	F1 Score
Comment Classification	0.89371	0.95016	0.93366	0.95025

Table 10: Human validation accuracy and agreement metrics for comment classification task.

State	Leaning	Is content poltical?	If political, is content about election and/or candidates?	Ideological stance	If political, Stance %	If political and about election and/or candidates, Stance %
Georgia	Democrat	32.0%	38.9%	Neutral	43.0%	14.7%
				Anti Republican	22.9%	36.8%
				Pro Democrat	16.8%	27.2%
				Anti Democrat	13.8%	16.0%
				Pro Republican	3.5%	5.3%
	Neutral	3.8%	5.3%	Neutral	68.6%	23.8%
				Anti Democrat	17.9%	29.5%
				Pro Republican	6.1%	22.9%
				Anti Republican	4.4%	13.3%
				Pro Democrat	2.9%	10.5%
	Republican	29.1%	39.4%	Anti Democrat	42.0%	51.6%
				Neutral	33.6%	11.5%
				Pro Republican	16.7%	26.1%
				Anti Republican	5.6%	7.6%
				Pro Democrat	2.1%	3.1%
New York	Democrat	26.5%	34.1%	Neutral	46.7%	16.6%
				Anti Republican	23.2%	40.2%
				Pro Democrat	13.8%	23.3%
				Anti Democrat	13.5%	14.5%
				Pro Republican	2.9%	5.3%
	Republican	21.7%	32.4%	Neutral	39.3%	14.0%
				Anti Democrat	38.3%	50.4%
				Pro Republican	13.3%	20.8%
				Anti Republican	6.2%	10.3%
				Pro Democrat	2.9%	4.5%
Texas	Democrat	28.1%	31.8%	Neutral	47.3%	16.8%
				Anti Republican	21.8%	38.2%
				Anti Democrat	15.8%	17.8%
				Pro Democrat	12.4%	22.1%
				Pro Republican	2.6%	5.1%
	Republican	24.9%	35.3%	Anti Democrat	41.5%	54.1%
				Neutral	34.9%	11.3%
				Pro Republican	15.9%	24.2%
				Anti Republican	5.6%	6.9%
				Pro Democrat	2.1%	3.6%

Table 11: For each experimental condition, the proportion of videos that are political, and of those, the proportion of videos that pertain to the US elections or major political candidates. For each set of videos, the distribution of their ideological stances.

**Month**	**Georgia**			**New York**		**Texas**	
	Neutral	Dem.	Rep.	Dem.	Rep.	Dem.	Rep.
May	0.04	-0.07	0.23	0.03	0.12	0.02	0.23
June	0.05	-0.11	0.21	-0.05	0.15	0.03	0.24
July	0.08	-0.06	0.24	0.03	0.21	-0.01	0.3
August	0.04	-0.13	0.28	-0.1	0.2	∼	∼
September	0.05	-0.2	0.44	-0.25	0.36	-0.21	0.39
October	0.04	-0.28	0.44	-0.2	0.38	-0.2	0.44
November	0.09	-0.12	0.52	-0.06	0.4	-0.05	0.46
**Overall**	0.05	-0.13	0.37	-0.08	0.26	-0.06	0.33

Table 12: The mean ideological content seen by bots of different conditioning in each state over time.

	**Georgia**			**New York**	**Texas**
	Rep. vs. Neutral	Dem. vs. Neutral	Rep. vs. Dem.	Rep. vs. Dem.	Rep. vs. Dem.
May	5.87***	-0.16	1.65	3.39**	5.62***
June	4.41**	2.05	1.34	2.07	5.89***
July	5.41***	-0.87	2.93*	6.29***	9.99***
August	8.48***	2.44*	3.73**	3.35**	∼
September	7.01***	4.81***	4.1***	2.25*	4.19**
October	6.27***	4.69***	1.98	3.06**	4.91***
November	5.81*	0.15	3.07*	3.71	4.06*
**Overall**	13.22***	4.7***	5.83***	5.83***	9.21***

Table 13: Independent t-test results comparing ideological content of bots of different conditioning in each state over time. ($* : p < 0.05$, $** : p < 0.01$, $*** : p < 0.001$)

Counterfactual model attribute (Recency scaling)	Ideological skew	Independent t-test Expected vs. Observed
Comment Count (Exponential)	-0.024	-11.922***
Comment Count (Linear)	-0.037	-12.479***
Comment Count (No scaling)	-0.009	-11.191***
Comments per recommendation (Exponential)	-0.05	-12.972***
Comments per recommendation (Linear)	-0.059	-13.373***
Comments per recommendation (No scaling)	-0.04	-12.403***
Combined Author Score (Exponential)	-0.059	-13.343***
Combined Author Score (Linear)	-0.065	-13.607***
Combined Author Score (No scaling)	-0.052	-13.067***
Combined Likes, Shares, Plays Score (Exponential)	0.008	-10.162***
Combined Likes, Shares, Plays Score (Linear)	-0.005	-10.884***
Combined Likes, Shares, Plays Score (No scaling)	0.032	-8.949***
Combined Video Score (Exponential)	-0.018	-11.63***
Combined Video Score (Linear)	-0.035	-12.36***
Combined Video Score (No scaling)	-0.001	-10.801***
Like Count (Exponential)	0.002	-10.438***
Like Count (Linear)	-0.01	-11.126***
Like Count (No scaling)	0.027	-9.238***
Video Length (Exponential)	-0.068	-13.926***
Video Length (Linear)	-0.08	-14.356***
Video Length (No scaling)	-0.07	-14.111***
(Likes + Comments + Shares) / Plays (Exponential)	-0.029	-12.142***
(Likes + Comments + Shares) / Plays (Linear)	-0.043	-12.788***
(Likes + Comments + Shares) / Plays (No scaling)	-0.009	-11.263***
Channel followers (Exponential)	-0.031	-12.282***
Channel followers (Linear)	-0.042	-12.672***
Channel followers (No scaling)	-0.015	-11.479***
Full Combined score (Exponential)	-0.051	-13.12***
Full Combined score (Linear)	-0.061	-13.55***
Full Combined score (No scaling)	-0.044	-12.815***
Channel cumul. likes (Exponential)	-0.05	-13.15***
Channel cumul. likes (Linear)	-0.057	-13.321***
Channel cumul. likes (No scaling)	-0.042	-12.925***
Likes per recommendation (Exponential)	-0.028	-12.114***
Likes per recommendation (Linear)	-0.042	-12.796***
Likes per recommendation (No scaling)	-0.009	-11.243***
Video plays (Exponential)	0.01	-9.976***
Video plays (Linear)	-0.004	-10.773***
Video plays (No scaling)	0.036	-8.714***
Video shares (Exponential)	0.006	-10.341***
Video shares (Linear)	-0.005	-10.972***
Video shares (No scaling)	0.029	-9.242***
Channel video count (Exponential)	-0.042	-12.728***
Channel video count (Linear)	-0.052	-13.077***
Channel video count (No scaling)	-0.03	-12.294***
Channel verified (Exponential)	-0.059	-11.519***
Channel verified (Linear)	-0.065	-11.803***
Channel verified (No scaling)	-0.05	-11.138***
Party-aligned comment proportion (Exponential)	-0.05	-9.771***
Party-aligned comment proportion (Linear)	-0.052	-9.84***
Party-aligned comment proportion (No scaling)	-0.049	-9.735***
Opposition comment proportion (Exponential)	-0.051	-8.608***
Opposition comment proportion (Linear)	-0.051	-8.622***
Opposition comment proportion (No scaling)	-0.051	-8.623***
**Observed**	**0.204**	

Table 14: The ideological skew observed in the experiments, as well as the ideological skew computed through counterfactual models which sample videos based on a given attribute. The right-most column denotes independent t-test results between the expected ideological skew and that observed by the bots. ($* : p < 0.05$, $** : p < 0.01$, $*** : p < 0.001$)

**Counterfactual model attribute (Recency scaling)**	**Ideological skew**	**Independent t-test Expected vs. Observed**
Comment Count (Exponential)	0.027	0.626
Comment Count (Linear)	0.017	0.061
Comment Count (No scaling)	0.071	3.364**
Comments per recommendation (Exponential)	-0.062	-5.025***
Comments per recommendation (Linear)	-0.067	-5.312***
Comments per recommendation (No scaling)	-0.046	-4.36***
Combined Author Score (Exponential)	-0.078	-5.762***
Combined Author Score (Linear)	-0.078	-6.041***
Combined Author Score (No scaling)	-0.069	-5.37***
Combined Likes, Shares, Plays Score (Exponential)	0.043	1.418
Combined Likes, Shares, Plays Score (Linear)	0.038	1.122
Combined Likes, Shares, Plays Score (No scaling)	0.089	4.159***
Combined Video Score (Exponential)	0.003	-0.788
Combined Video Score (Linear)	-0.022	-2.227*
Combined Video Score (No scaling)	0.03	1.053
Like Count (Exponential)	0.034	0.965
Like Count (Linear)	0.029	0.652
Like Count (No scaling)	0.079	3.577***
Video Length (Exponential)	-0.065	-5.162***
Video Length (Linear)	-0.082	-5.796***
Video Length (No scaling)	-0.068	-6.513***
(Likes + Comments + Shares) / Plays (Exponential)	-0.047	-4.074***
(Likes + Comments + Shares) / Plays (Linear)	-0.06	-4.636***
(Likes + Comments + Shares) / Plays (No scaling)	-0.031	-3.711***
Channel followers (Exponential)	-0.014	-2.082*
Channel followers (Linear)	-0.028	-3.031**
Channel followers (No scaling)	0.016	0.015
Full Combined score (Exponential)	-0.068	-5.339***
Full Combined score (Linear)	-0.073	-5.758***
Full Combined score (No scaling)	-0.058	-5.057***
Channel cumul. likes (Exponential)	-0.019	-2.206*
Channel cumul. likes (Linear)	-0.033	-3.2**
Channel cumul. likes (No scaling)	0.008	-0.591
Likes per recommendation (Exponential)	-0.046	-3.989***
Likes per recommendation (Linear)	-0.059	-4.602***
Likes per recommendation (No scaling)	-0.034	-3.938***
Video plays (Exponential)	0.052	1.87
Video plays (Linear)	0.048	1.575
Video plays (No scaling)	0.102	4.728***
Video shares (Exponential)	0.042	1.329
Video shares (Linear)	0.035	0.984
Video shares (No scaling)	0.081	3.841***
Channel video count (Exponential)	0.025	0.511
Channel video count (Linear)	0.005	-0.703
Channel video count (No scaling)	0.058	2.9**
Channel verified (Exponential)	-0.074	-5.459***
Channel verified (Linear)	-0.081	-5.941***
Channel verified (No scaling)	-0.066	-5.011***
Party-aligned comment proportion (Exponential)	0.018	0.119
Party-aligned comment proportion (Linear)	0.001	-0.874
Party-aligned comment proportion (No scaling)	0.02	0.245
Opposition comment proportion (Exponential)	-0.016	-1.62
Opposition comment proportion (Linear)	-0.022	-1.963
Opposition comment proportion (No scaling)	-0.016	-1.653
**Observed**	**0.0159**	

Table 15: The ideological skew observed in the experiments, as well as the ideological skew computed through counterfactual models which sample videos based on a given attribute when only considering positive-partisanship videos (Pro Democrat or Pro Republican). The right-most column denotes independent t-test results between the expected ideological skew and that observed by the bots. ($* : p < 0.05$, $** : p < 0.01$, $*** : p < 0.001$)

Counterfactual model attribute (Recency scaling)	Ideological skew	Independent t-test Expected vs. Observed
Comment Count (Exponential)	-0.033	-17.873***
Comment Count (Linear)	-0.046	-17.981***
Comment Count (No scaling)	-0.027	-17.39***
Comments per recommendation (Exponential)	-0.045	-18.194***
Comments per recommendation (Linear)	-0.054	-18.326***
Comments per recommendation (No scaling)	-0.039	-17.763***
Combined Author Score (Exponential)	-0.052	-18.875***
Combined Author Score (Linear)	-0.058	-19.009***
Combined Author Score (No scaling)	-0.048	-19.066***
Combined Likes, Shares, Plays Score (Exponential)	0.002	-15.192***
Combined Likes, Shares, Plays Score (Linear)	-0.012	-16.356***
Combined Likes, Shares, Plays Score (No scaling)	0.021	-14.131***
Combined Video Score (Exponential)	-0.02	-17.043***
Combined Video Score (Linear)	-0.034	-17.445***
Combined Video Score (No scaling)	-0.006	-16.478***
Like Count (Exponential)	-0.002	-15.511***
Like Count (Linear)	-0.015	-16.654***
Like Count (No scaling)	0.016	-14.501***
Video Length (Exponential)	-0.065	-19.538***
Video Length (Linear)	-0.075	-19.655***
Video Length (No scaling)	-0.068	-20.294***
(Likes + Comments + Shares) / Plays (Exponential)	-0.022	-17.163***
(Likes + Comments + Shares) / Plays (Linear)	-0.035	-17.954***
(Likes + Comments + Shares) / Plays (No scaling)	-0.004	-16.426***
Channel followers (Exponential)	-0.033	-18.069***
Channel followers (Linear)	-0.041	-18.175***
Channel followers (No scaling)	-0.021	-17.557***
Full Combined score (Exponential)	-0.045	-18.723***
Full Combined score (Linear)	-0.054	-19.052***
Full Combined score (No scaling)	-0.04	-18.874***
Channel cumul. likes (Exponential)	-0.054	-19.356***
Channel cumul. likes (Linear)	-0.059	-19.191***
Channel cumul. likes (No scaling)	-0.054	-19.995***
Likes per recommendation (Exponential)	-0.022	-17.15***
Likes per recommendation (Linear)	-0.035	-18.002***
Likes per recommendation (No scaling)	-0.002	-16.342***
Video plays (Exponential)	0.003	-15.049***
Video plays (Linear)	-0.012	-16.294***
Video plays (No scaling)	0.022	-13.918***
Video shares (Exponential)	0.001	-15.407***
Video shares (Linear)	-0.011	-16.346***
Video shares (No scaling)	0.018	-14.478***
Channel video count (Exponential)	-0.054	-19.207***
Channel video count (Linear)	-0.061	-18.856***
Channel video count (No scaling)	-0.051	-19.586***
Channel verified (Exponential)	-0.052	-16.929***
Channel verified (Linear)	-0.057	-17.276***
Channel verified (No scaling)	-0.044	-16.432***
Party-aligned comment proportion (Exponential)	-0.068	-15.53***
Party-aligned comment proportion (Linear)	-0.064	-15.275***
Party-aligned comment proportion (No scaling)	-0.068	-15.431***
Opposition comment proportion (Exponential)	-0.063	-13.608***
Opposition comment proportion (Linear)	-0.06	-13.466***
Opposition comment proportion (No scaling)	-0.064	-13.646***
**Observed**	**0.1882**	

Table 16: The ideological skew observed in the experiments, as well as the ideological skew computed through counterfactual models which sample videos based on a given attribute when only considering negative-partisanship videos (Anti Democrat or Anti Republican). The right-most column denotes independent t-test results between the expected ideological skew and that observed by the bots. ($*: p < 0.05$, $**: p < 0.01$, $***: p < 0.001$)

Counterfactual model based on weight of Channel Verification status (Recency scaling)	Ideological skew	Independent t-test Expected vs. Observed
Weight = 0.55 (Exponential)	-0.058	-11.238***
Weight = 0.55 (Linear)	-0.065	-11.549***
Weight = 0.55 (No scaling)	-0.049	-10.881***
Weight = 0.6 (Exponential)	-0.058	-11.25***
Weight = 0.6 (Linear)	-0.065	-11.553***
Weight = 0.6 (No scaling)	-0.05	-10.888***
Weight = 0.65 (Exponential)	-0.059	-11.259***
Weight = 0.65 (Linear)	-0.065	-11.537***
Weight = 0.65 (No scaling)	-0.05	-10.9***
Weight = 0.7 (Exponential)	-0.058	-11.244***
Weight = 0.7 (Linear)	-0.065	-11.558***
Weight = 0.7 (No scaling)	-0.049	-10.87***
Weight = 0.75 (Exponential)	-0.058	-11.237***
Weight = 0.75 (Linear)	-0.065	-11.546***
Weight = 0.75 (No scaling)	-0.049	-10.873***
Weight = 0.8 (Exponential)	-0.058	-11.238***
Weight = 0.8 (Linear)	-0.065	-11.557***
Weight = 0.8 (No scaling)	-0.05	-10.908***
Weight = 0.85 (Exponential)	-0.058	-11.248***
Weight = 0.85 (Linear)	-0.065	-11.532***
Weight = 0.85 (No scaling)	-0.049	-10.879***
Weight = 0.9 (Exponential)	-0.058	-11.237***
Weight = 0.9 (Linear)	-0.065	-11.552***
Weight = 0.9 (No scaling)	-0.049	-10.87***
Weight = 0.95 (Exponential)	-0.058	-11.243***
Weight = 0.95 (Linear)	-0.065	-11.548***
Weight = 0.95 (No scaling)	-0.05	-10.905***
Weight = 1.0 (Exponential)	-0.058	-11.256***
Weight = 1.0 (Linear)	-0.065	-11.548***
Weight = 1.0 (No scaling)	-0.05	-10.89***
**Observed**	**0.204**	

Table 17: The ideological skew observed in the experiments, as well as the ideological skew computed through counterfactual models which sample videos based on channel verification status with varying weights given to a channels verification status. The right-most column denotes independent t-test results between the expected ideological skew and that observed by the bots. ($*: p < 0.05$, $**: p < 0.01$, $***: p < 0.001$)

Republican hashtag	Count	Democrat hashtag	Count
foryou	173	jamaalbowman	163
fyp :)	173	therentistoodamnhigh	163
usa	173	donaldtrump	159
trump2024	173	supremecourt	158
country	173	immigration	157
america	173	trump	155
fyp	173	moreteachinglesstesting	155
trump	173	emmys	155
politics	172	jonstewart	155
biden	170	theproblem	155
fypdoesntwork	168	theproblemwithjonstewart	154
republican	168	transgenderrights	154
military	168	fyp	154
backtheblue	168	climatechange	153
facts	168	climate	153
opinion	166	migrantes	153
w	166	scotus	153
president	166	appletv	153
4u	166	tiktok	153
conservative	160	strictscrutiny	153
duet	160	law	153
army	159	breakingnews	153
police	159	biden	153
stitch	155	elections	153
donaldtrump	154	outstandingtalkseries	153
tucker	153	peace	153
consantanderconecto	153	podcast	153
tuckercarlson	153	globalwarming	153
tiktokban	152	fyc	153
reaction	149	economy	152

Table 18: The 20 most common hashtags for Democrat and Republican conditioning videos.

Topic	Prop. of Dem. videos	Prop. of Rep. videos	Difference	χ^2 test
Abortion and reproductive health	6.6	1.5	5.1	172.0***
Government or politics generally	43.9	42.3	1.6	1.02
Biden dropping out of the presidential race	6.6	5.2	1.4	7.41**
Climate change	1.6	0.6	1.0	25.78***
Education	1.8	1.2	0.6	6.66**
Economy generally	6.2	5.7	0.5	1.08
Racial issues, including affirmative action and racial discrimination	5.8	5.4	0.5	0.87
Other public health issues	1.4	1.0	0.5	4.98*
Democratic National Convention (DNC)	1.1	0.7	0.4	5.39*
Guns and gun control	1.6	1.2	0.4	2.75
Environment generally	0.6	0.4	0.2	1.73
LGBTQ+ issues, including transgender issues	2.4	2.5	-0.0	0.0
Republican National Convention (RNC)	0.4	0.4	0.0	0.0
AI, LLMs	0.1	0.2	-0.0	0.07
Crypto	0.1	0.1	-0.0	0.0
Other vaccines	0.0	0.2	-0.1	3.15
Covid, including covid vaccines	0.8	1.0	-0.2	0.74
Other technology issues	0.3	0.6	-0.3	3.48
Assassination attempt on Donald Trump	1.1	1.5	-0.4	3.13
Crime generally	3.6	4.4	-0.8	3.62
Other	0.2	1.2	-1.0	30.38***
Other social issues, including culture war issues, labor, and other social issues that are not covered above	5.7	6.9	-1.2	5.55*
Ukraine war	0.4	1.5	-1.2	33.14***
Israel, Gaza or Palestine, including anything about Netanyahu or Hamas	2.3	4.3	-2.0	29.21***
Immigration	3.5	5.7	-2.2	24.66***
Anything outside the US or involve US foreign relations except for Israel, Gaza, Ukraine, or immigration	1.8	4.6	-2.8	57.05***

Table 19: The proportion of Democrat and Republican videos on a given topic, the difference in these proportions, and chi-squared tests comparing these proportions. ($* : p < 0.05$, $** : p < 0.01$, $*** : p < 0.001$)

Variable	VIF
Conditioning stage video play count	37.0054
Conditioning stage video like count	35.5774
Recommendation stage video play count	14.9198
Conditioning stage video share count	13.8981
Conditioning stage channel verification status	10.5766
Conditioning stage channel video count	8.3425
Conditioning stage channel cumulative like count	7.0947
Proportion of videos with negative-partisanship in seeding stage	6.7599
Recommendation stage video comment count	6.3564
Recommendation stage channel cumulative like count	5.6784
Conditioning stage channel follower count	5.0894
Conditioning stage video comment count	4.0431
Recommendation stage channel video count	3.9704
Recommendation stage video share count	3.1209
Recommendation stage channel follower count	3.0829
Recommendation stage video likes per recommendation	1.4819
Recommendation stage video comments per recommendation	1.4176
Number of videos watched during conditioning stage	1.3931
Opposition comment proportion	1.2967
Recommendation stage channel verification status	1.1898
Number of videos watched during recommendation stage	1.1710
Video Duration	1.0483
Transcript availability rate	1.0291

Table 20: VIF values for control variables in linear probability model considering all videos viewed during experiment.

Variable	VIF
Conditioning stage video like count	35.8474
Recommendation stage video play count	15.0881
Conditioning stage video share count	13.9690
Conditioning stage video comment count	12.6072
Recommendation stage video like count	11.7736
Conditioning stage channel verification status	10.8042
Conditioning stage channel video count	8.3650
Conditioning stage channel cumulative like count	7.1172
Recommendation stage video comment count	6.3587
Recommendation stage channel cumulative like count	5.6939
Conditioning stage channel follower count	5.1481
Recommendation stage channel video count	4.3401
Conditioning stage video play count	4.0491
Recommendation stage channel follower count	3.2246
Recommendation stage video share count	3.1259
Top account X Republican bot	1.7722
Top account X Democrat bot	1.6720
Recommendation stage video likes per recommendation	1.4850
Recommendation stage video comments per recommendation	1.4286
Number of videos watched during conditioning stage	1.4073
Recommendation stage channel verification status	1.3980
Opposition comment proportion	1.3077
Number of videos watched during recommendation stage	1.1715
Transcript availability rate	1.1214
Video Duration	1.0502

Table 21: VIF values for control variables in linear probability model when considering top account status.

Parameter	Est.	Unadjusted p-value	Benjamini-Hochberg adjusted p-value	Std. error
**Bot conditioning (ref = Rep.)**				
Democrat	.3080	.0002	.0020	.0669
**State (ref = NY)**				
Texas	.0069	.5865	.7820	.0124
Georgia	.0268	.1618	.2866	.0183
**Conditioning metrics**				
Proportion of negative partisanship videos	.0716	.0219	.0585	.0284
Number of conditioning videos watched	-.02827	.0196	.05854	.0109
Conditioning videos engagement score	-.01429	.1791	.2866	.0102
**Recommendation video metrics**				
Number of videos watched	.0002	.9863	.9863	.0130
Transcript availability rate	.0017	.9130	.9740	.0157
Likes per recommendation	-.01590	.0781	.1564	.0084
Comments per recommendation	.0070	.7498	.8570	.0218
Opposing comment proportion	.1262	$p < 0.0001$	$p < 0.0001$	.0125
Video Length	-.02006	.0548	.1254	.0097
**Recommendation channel metrics**				
Video count	.0383	.0057	.0306	.0121
Follower count	.0039	.7099	.8570	.0104
Share count	-.0122	.4502	.6550	.0158

Table 22: Linear Probability Model estimating the likelihood of cross-party recommendations across bot political alignments.

Parameter	Est.	Unadjusted p-value	Benjamini-Hochberg adjusted p-value
**Bot conditioning (Ref = Rep.)**			
Democrat	2.1263	$p < 0.0001$	$p < 0.0001$
**State (ref = NY)**			
Texas	.0984	.3572	.4762
Georgia	.2134	.0572	.0916
**Conditioning metrics**			
Proportion of negative partisanship videos	.5157	$p < 0.0001$	$p < 0.0001$
Number of conditioning videos watched	-.1778	.0005	.0013
Conditioning videos engagement score	-.0969	.1965	.2858
**Recommendation metrics**			
Number of videos watched	.0190	.6757	.6757
Transcript availability rate	.0209	.6410	.6757
Likes per recommendation	-.1240	.0267	.0534
Comments per recommendation	.0311	.6613	.6757
Opposing comment proportion	.7058	$p < 0.0001$	$p < 0.0001$
Video length	-.1575	.0105	.0240
**Recommendation channel metrics**			
Video count	.2102	$p < 0.0001$	.0001
Follower count	.0267	.6323	.6757
Share count	-.0901	.0533	.0916

Table 23: Logit model estimating the likelihood of cross-party recommendations across bot political alignments.

Parameter	Est.	Unadjusted p-value	Benjamini-Hochberg adjusted p-value	Std. error
**Bot conditioning (ref = Rep.)**				
Democrat	.3476	$p < 0.0001$	$p < 0.0001$	.0565
**State (ref = NY)**				
Texas	.0078	.5752	.6902	.0136
Georgia	.0219	.2047	.2853	.0166
**Top account x Bot conditioning**				
Top account x Democrat bot	-.0720	$p < 0.0001$	$p < 0.0001$	.0111
Top account x Republican bot	-.0141	.2060	.2853	.0107
**Conditioning metrics**				
Proportion of negative partisanship videos	.0572	.0274	.0988	.0237
Conditioning videos engagement score	-.0125	.2037	.2853	.0094
Number of conditioning videos watched	-.0218	.0441	.1324	.0100
**Recommendation video metrics**				
Number of videos watched	-.0011	.9191	.9219	.0110
Transcript availability rate	.0039	.7785	.8576	.0136
Likes per recommendation	-.0152	.1031	.2321	.0088
Comments per recommendation	-.0021	.9218	.9219	.0215
Opposing comment proportion	.1313	$p < 0.0001$	$p < 0.0001$	.0141
Video Length	-.0224	.0627	.1613	.0112
**Recommendation channel metrics**				
Video count	.0196	.1714	.2853	.0137
Follower count	.0218	.1311	.2623	.0137
Share count	-.0114	.4731	.6083	.0155

Table 24: Linear Probability Model estimating the likelihood of cross-party recommendations across bot political alignments when accounting for top Republican and Democrat channels.

Parameter	Est.	Unadjusted p-value	Benjamini-Hochberg adjusted p-value
**Bot conditioning (Ref = Rep.)**			
Democrat	2.2805	$p < 0.0001$	$p < 0.0001$
**State (ref = NY)**			
Texas	.1209	.2662	.3194
Georgia	.1956	.0860	.1291
**Top account x Bot conditioning**			
Top account x Democrat	-.4096	$p < 0.0001$	$p < 0.0001$
Top account x Republican	-.1191	.0428	.0771
**Conditioning metrics**			
Proportion of negative partisanship videos	.4235	.0002	.0009
Number of conditioning videos watched	-.1447	.0053	.0138
Conditioning videos engagement score	-.0882	.2409	.3098
**Recommendation metrics**			
Number of videos watched	.0073	.8740	.8740
Transcript availability rate	.0329	.4699	.5287
Likes per recommendation	-.1291	.0224	.0448
Comments per recommendation	-.0205	.7827	.8288
Opposing comment proportion	.7601	$p < 0.0001$	$p < 0.0001$
Video length	-.2071	.0023	.0069
**Recommendation channel metrics**			
Video count	.0866	.1199	.1660
Follower count	.1364	.0188	.0423
Share count	-.0846	.0652	.1067

Table 25: Logit model estimating the likelihood of cross-party recommendations across bot political alignments when accounting for top Republican and Democrat channels.

Parameter	Est.	Unadjusted p-value	Benjamini-Hochberg adjusted p-value	Std. error
**Bot conditioning (ref = Rep.)**				
Democrat	.1959	.0023	.0093	.0547
**State (ref = NY)**				
Texas	.0040	.7802	.8148	.0143
Georgia	.0181	.2988	.3984	.0169
**Top account x Bot conditioning**				
Top account x Democrat bot	-.0641	$p < 0.0001$	$p < 0.0001$	.0099
Top account x Republican bot	-.0160	.1736	.2894	.0112
**Partisanship x Bot conditioning**				
Negative partisanship x Democrat bot	.1014	$p < 0.0001$	.0004	.0198
Negative partisanship x Republican bot	.0160	.0737	.1844	.0084
**Conditioning metrics**				
Proportion of negative partisanship videos	.0523	.0213	.0710	.0206
Conditioning videos engagement score	-.0117	.2004	.3084	.0088
Number of conditioning videos watched	-.0206	.0338	.0968	.0089
**Recommendation video metrics**				
Number of videos watched	-.0022	.8147	.8148	.0094
Transcript availability rate	.0043	.7249	.8148	.0120
Likes per recommendation	-.0135	.1735	.2893	.0095
Comments per recommendation	.0059	.7832	.8148	.0214
Opposing comment proportion	.1375	$p < 0.0001$	$p < 0.0001$	.0138
Video Length	-.0187	.1110	.2221	.0111
**Recommendation channel metrics**				
Video count	.0142	.3694	.4619	.0154
Follower count	.0304	.0865	.1923	.0167
Share count	-.0188	.2922	.3984	.0173

Table 26: Linear Probability Model estimating the likelihood of cross-party recommendations across bot political alignments when accounting for top Republican and Democrat channels and video partisanship.

Parameter	Est.	Unadjusted p-value	Benjamini-Hochberg adjusted p-value
**Bot conditioning (Ref = Rep.)**			
Democrat	1.3373	$p < 0.0001$	$p < 0.0001$
**State (ref = NY)**			
Texas	.0998	.3874	.4843
Georgia	.1765	.0909	.1653
**Top account x Bot conditioning**			
Top account x Democrat	-.3757	$p < 0.0001$	$p < 0.0001$
Top account x Republican	-.1390	.1144	.1907
**Partisanship x Bot conditioning**			
Negative partisanship x Democrat bot	.6578	$p < 0.0001$	$p < 0.0001$
Negative partisanship x Republican bot	.1406	.0186	.0465
**Conditioning metrics**			
Proportion of negative partisanship videos	.4005	.0015	.0052
Number of conditioning videos watched	-.1379	.0112	.0322
Conditioning videos engagement score	-.0930	.3056	.4074
**Recommendation metrics**			
Number of videos watched	-.0066	.9101	.9101
Transcript availability rate	.0314	.6430	.6769
Likes per recommendation	-.1191	.1395	.2147
Comments per recommendation	.0537	.5877	.6677
Opposing comment proportion	.8395	$p < 0.0001$	$p < 0.0001$
Video length	-.2146	.0820	.1641
**Recommendation channel metrics**			
Video count	.0537	.6009	.6677
Follower count	.2086	.0395	.0878
Share count	-.1587	.1568	.2240

Table 27: Logit model estimating the likelihood of cross-party recommendations across bot political alignments when accounting for top Republican and Democrat channels and video partisanship.

		Democrat bots	Neutral bots	Republican bots
	Unique videos	1847	8177	2640
Video play count	mean	2413648.8843	5637526.6721	2707925.1944
	std	7506332.0558	19249220.6577	5683604.0638
	min	239.0	48.0	516.0
	25%	67200.0	190250.0	103725.0
	50%	409400.0	970600.0	497650.0
	75%	1500000.0	3700000.0	2400000.0
	max	56200000.0	177600000.0	56600000.0
Video share count	mean	18692.9357	45206.1413	21480.1101
	std	64829.2859	160370.5541	67609.5854
	min	0.0	0.0	1.0
	25%	256.25	572.0	363.0
	50%	1700.5	4151.0	2903.0
	75%	10475.0	21400.0	15000.0
	max	586900.0	2200000.0	1800000.0
Video like count	mean	250751.5964	643048.7369	253244.4459
	std	735713.9236	2327330.5311	560122.1661
	min	21.0	2.0	2.0
	25%	6954.0	14900.0	11300.0
	50%	39900.0	87100.0	50950.0
	75%	144000.0	376050.0	214150.0
	max	4700000.0	24200000.0	8700000.0
Video likes per recommendation	mean	0.1183	0.1206	0.1172
	std	0.0596	0.0821	0.0622
	min	0.0125	0.0012	0.0029
	25%	0.0737	0.0563	0.0695
	50%	0.1083	0.1045	0.1052
	75%	0.1567	0.169	0.1536
	max	0.4994	0.7676	0.3916
Video comment count	mean	7728.3862	7935.9829	7235.3002
	std	18115.1643	23225.5984	13568.1027
	min	0.0	0.0	0.0
	25%	414.75	403.0	523.0
	50%	2108.5	1730.0	2384.0
	75%	6759.5	5733.5	8080.5
	max	131200.0	773000.0	184300.0
Video comments per recommendation	mean	0.0078	0.0063	0.0071
	std	0.0099	0.0153	0.0088
	min	0.0	0.0	0.0
	25%	0.0025	0.0007	0.0019
	50%	0.005	0.0018	0.0043
	75%	0.0097	0.0049	0.0088
	max	0.164	0.2166	0.1164

Table 28: Engagement metrics of videos watched during recommendation stage.

		**Democrat bots**	**Neutral bots**	**Republican bots**
	Unique channels	640	5644	1197
Channel follower count	mean	3036086.1187	3075284.5458	3028526.2507
	std	3264755.0004	5075331.3993	4228698.84
	min	2102.0	32.0	746.0
	25%	665550.0	212400.0	618200.0
	50%	1500000.0	1000000.0	1300000.0
	75%	4100000.0	4100000.0	3300000.0
	max	16700000.0	75300000.0	42200000.0
Channel cumulative like count	mean	146046830.1019	150456706.5605	125131927.1811
	std	222423644.7813	322884691.3661	306548628.1349
	min	18900.0	0.0	0.0
	25%	11800000.0	6100000.0	13800000.0
	50%	63700000.0	38750000.0	47200000.0
	75%	265900000.0	153400000.0	107200000.0
	max	1700000000.0	1900000000.0	1700000000.0
Channel video count	mean	2121.7534	1977.6035	1852.6265
	std	2319.6505	2939.3987	2635.1561
	min	23.0	3.0	16.0
	25%	546.5	286.0	447.0
	50%	1197.0	820.0	1025.0
	75%	3413.0	2605.0	2605.0
	max	24700.0	30700.0	16300.0
Channel verification status	count	70	222	75

Table 29: Engagement metrics of channels watched during recommendation stage.

		Democrat bots	Republican bots
	Unique videos	1534	2561
Video play count	mean	647921.0081	563931.6978
	std	2103402.7393	5879274.2821
	min	619.0	435.0
	25%	12600.0	8043.0
	50%	58300.0	26800.0
	75%	366400.0	126400.0
	max	174500000.0	1100000000.0
Video share count	mean	4753.1959	4515.1846
	std	20217.5614	44045.2628
	min	1.0	0.0
	25%	142.0	70.0
	50%	350.0	175.0
	75%	2180.0	698.0
	max	1400000.0	4600000.0
Video like count	mean	93469.9648	56878.0389
	std	295319.8032	405934.5273
	min	4.0	9.0
	25%	1458.0	640.0
	50%	6865.0	2810.0
	75%	44400.0	13000.0
	max	21800000.0	33400000.0
Video likes per recommendation	mean	0.1364	0.1059
	std	0.0595	0.052
	min	0.0036	0.0003
	25%	0.0914	0.0715
	50%	0.133	0.0971
	75%	0.1791	0.1337
	max	0.365	0.4419
Video comment count	mean	2580.4743	1261.7448
	std	6773.3462	4909.7431
	min	0.0	0.0
	25%	38.0	33.0
	50%	244.0	106.0
	75%	1582.0	469.0
	max	109800.0	187700.0
Video comments per recommendation	mean	0.0054	0.0059
	std	0.0044	0.0075
	min	0.0	0.0
	25%	0.0021	0.0018
	50%	0.0044	0.004
	75%	0.0075	0.0075
	max	0.0395	0.1184

Table 30: Engagement metrics of videos watched during conditioning stage.

		**Democrat**	**Republican**
	Unique channels	12	12
Channel follower count	mean	928811.6177	1114729.5803
	std	678815.8153	982530.0651
	min	0.0	0.0
	25%	384100.0	385100.0
	50%	721900.0	720000.0
	75%	1400000.0	2200000.0
	max	27700000.0	49200000.0
Channel cumulative like count	mean	17632176.9303	25118611.7036
	std	20284093.3183	26866917.3477
	min	2288.0	0.0
	25%	7000000.0	8600000.0
	50%	10900000.0	14900000.0
	75%	26500000.0	47200000.0
	max	1500000000.0	1900000000.0
Channel video count	mean	421.0464	1218.6955
	std	377.7008	963.9262
	min	0.0	0.0
	25%	121.0	345.0
	50%	331.0	955.0
	75%	604.0	2605.0
	max	6377.0	30700.0
Channel verification status	count	11	2

Table 31: Engagement metrics of channels watched during conditioning stage.

Metric	Anti-Dem	Anti-Rep	Pro-Dem	Pro-Rep	Negative	Positive
**Views**	1286490.9	935614.7	825041.1	1365280.4	1159497.7	1074803.4
**Likes**	133539.7	103084.7	95966.5	119514.8	122517.1	106853.3
**Comments**	3790.4	3719.6	3399.5	4191.3	3764.8	3765.6
**Likes per View**	0.1176	0.1212	0.1286	0.1141	0.1189	0.1219
**Comments per View**	0.0074	0.0082	0.0096	0.0091	0.0077	0.0094

Table 32: Average engagement metrics for videos of different ideological stances on TikTok

	Free response questions			
Variable	Mentioned changes to political content when asked about content overall	Mentioned shifts towards co-partisan content when asked about content overall	Mentioned shifts towards co-partisan content when asked about political content	Mentioned shifts to positive content
Intercept	-1.286**	0.011	-0.094	-0.202
Republican Party (ref. Democratic Party)	-0.353*	0.024	0.08**	0.274***
Age (ref. 65+ years old)				
18-24 years old	0.099	-0.04	0.071	-0.035
25-34 years old	0.486	-0.027	0.036	0.051
35-44 years old	0.464	-0.021	0.024	0.044
45-54 years old	0.718	-0.003	-0.001	0.011
55-64 years old	0.364	-0.015	0.062	-0.072
Race (ref. White)				
Asian	1.013**	0.1	0.152	-0.266*
Black	-0.074	0.002	0.025	0.001
Hispanic	0.243	-0.03	0.06	-0.377**
Native American	-0.558	-0.005	-0.038	0.144
Two or more races	0.891	-0.0	-0.089	-0.456*
Other	-0.123	-0.003	-0.009	0.242
Prefer not to answer	-237.16***	0.009	0.016	0.15*
Female (ref. Male)	0.036	0.007	0.042	0.0
Education (ref. Bachelor's degree)				
Associates or technical degree	0.381	0.009	0.07	-0.247*
Graduate or professional degree	-0.345*	-0.006	0.026	0.078
High school diploma or GED	-0.38	-0.027	-0.071	-0.224*
Some college, but no degree	0.133	-0.006	-0.017	-0.119
Some high school or less	-0.75	-0.013	0.175	0.367
Prefer not to say	-240.424***	-0.021	-0.052	0.883*

Table 33: Regression coefficients for various models estimating responses to free-response (text-entry) questions based on participant demographics; (Q1 - Q3) of Survey. * : $p < 0.05$; **: $p < 0.01$; ***: $p < 0.001$

Variable	More co-partisan/ cross-partisan content	More Pro-Trump More Anti-Trump content	More political/ Less political content	More positive/ More negative content	More optimistic/ More pessimistic content	More content you agree /disagree with
	Structured questions					
Intercept	0.394	4.755***	7.25***	4.68***	4.575***	6.267***
Republican Party (ref. Democratic Party)	1.445***	1.071***	-0.047	1.029***	0.994***	0.358*
Age (ref. 65+ years old)						
18-24 years old	0.38	0.174	-0.13	-0.38	0.114	-0.018
25-34 years old	-0.236	-0.172	-0.388	-0.527	-0.091	-0.376
35-44 years old	-0.416	0.073	-0.29	-0.628	-0.026	-0.386
45-54 years old	-0.677	-0.166	-0.491	-0.698	-0.219	-0.895*
55-64 years old	-0.228	-0.639	-0.077	-0.531	-0.019	-0.368
Race (ref. White)						
Asian	0.356	-1.156*	-1.112*	-1.012*	-1.726***	-0.497
Black	0.139	-0.329	0.164	0.062	0.263	0.02
Hispanic	-0.751	0.418	0.418	-0.516	0.018	-0.251
Native American	0.715	-0.702	-0.204	0.619	1.253	0.043
Two or more races	-1.812*	0.317	0.476	-1.49*	-1.123	-2.146**
Other	0.583	1.428	-1.188	1.182	1.811	0.657
Prefer not to answer	-0.152	-1.416	2.507*	-2.141	-2.587	-4.043
Female (ref. Male)	-0.006	-0.167	-0.369*	-0.012	0.103	0.152
Education (ref. Bachelor's degree)						
Associates or technical degree	0.337	-0.555	-0.787	-0.325	-0.562	-0.274
Graduate or professional degree	0.636***	0.172	0.341*	0.424*	0.541**	0.546**
High school diploma or GED	-0.332	-0.765*	-0.134	-0.73*	-0.367	-0.246
Some college, but no degree	0.362	-0.364	-0.663	-0.075	-0.414	-0.047
Some high school or less	0.66	-0.969	-3.007*	-0.079	-0.567	-0.725
Prefer not to say	2.583***	2.269***	-5.543*	3.932***	-4.645	2.609*

Table 34: Regression coefficients for various models estimating responses to structured (scale-based) questions based on participant demographics; (Q4 - Q9) of Survey. * : $p < 0.05$; **: $p < 0.01$; ***: $p < 0.001$

Demographic	Count
25-34 years old	373
35-44 years old	232
45-54 years old	173
18-24 years old	109
55-64 years old	86
65+ years old	35
Female	547
Male	449
Non-binary / third gender	12
White	646
Black	276
Asian	28
Hispanic	25
Two or more races	21
Native American	7
Other	5
Bachelor's degree	417
Graduate or professional degree	412
Some college, but no degree	74
High school diploma or GED	55
Associates or technical degree	46
Some high school or less	3
Prefer not to say	1
Party	
Republican Party	552
Democratic Party	317
Independent	139
Democrat Strong	
Strong Democrat	210
Not so strong Democrat	106
Prefer not to answer	1
Republican Strong	
Strong Republican	346
Not so strong Republican	202
Prefer not to answer	4
Independent close to Democrat or Republican	
Neither	66
Democratic Party	35
Republican Party	34
Not sure	3
Prefer not to answer	1
Liberal/Conservative	
Conservative	341
Liberal	215
Moderate	178
Very conservative	160
Very liberal	108
Not sure	5
Prefer not to answer	1
Voted in 2024 elections	
Yes	924
No	64
Prefer not to answer	20
Party in 2024 elections	
Republican	590
Democrat	307
Independent	15
Other	6
Libertarian	4
Green	2
Plans to vote in 2026 elections	
Yes	798
Not sure	152
No	46
Prefer not to answer	12
Party in 2026 elections	
Republican	449
Democrat	290
Independent	39
Other	15
Libertarian	4
Green	1

Table 35: Participant demographic counts

Left-leaning Account	Right-leaning Account	Euclidean Distance
indianahousedemocrats	republicantoks	3.83K
repbowman	elsakurt_official	13.90K
repstansbury	republicanfamily	13.94K
repsumnerlee	republican_army_	20.73K
iampoliticsgirl	therepublicanjournal	209.84K
bernie	tuckercarlson	608.28K
theproblem	donaldtrumpwasright	636.73K
reprokhanna	kayetriots	707.32K
aocinthehouse	restoringamerica	920.82K
thedemocrats	thecamhigby	1.6M
biancagraulau	conservativeant2.0	2.4M
jeffjacksonnc	real.benshapiro	13.5M

Table 36: Left- and right-leaning TikTok accounts used during the conditioning phase, paired based on the minimum Euclidean distance with respect to the number of followers and likes.

State	Democrat bots	Republican bots
Georgia	379.15	300.51
New York	350.78	312.81
Texas	381.4	314.16

Table 37: The mean number of conditioning videos watched per experimental condition.

Category	Count	Proportion ($M \pm SD$)
Democrat-aligned	56	0.925 ± 0.0733
Republican-aligned	75	0.94 ± 0.0568

Table 38: Classification of TikTok channels based on political videos, including channel counts and average proportions of partisan content.

Engagement metric	True diff. in normalized metric values (Rep - Dem)	Required Scaling with different distributions			
		Binomial	Lognormal	Normal	Poisson
(Comments, Likes, Shares, Plays) Score	0.0209	31.2593	9.4323	34.0259	29.03865
Video share count	0.0028	169.4288	51.1241	184.4241	157.3927
Video play count	0.0024	177.4398	53.5413	193.1442	164.8346
Comment count	0.0006	782.0713	235.9851	851.2887	726.5135
Video Like count	0.0009	325.8119	98.3117	354.6479	302.6664
Comments per recommendation	-0.0005	-77.8383	-23.4872	-84.7274	-72.3087
Video engagement rate	-0.0016	-70.573	-21.295	-76.8191	-65.5596
Channel follower count	-0.0017	-122.0704	-36.834	-132.8742	-113.3986
Likes per recommendation	-0.0026	-73.7079	-22.2409	-80.2314	-68.4717
Channel cumul. likes	-0.0116	-48.4503	-14.6196	-52.7384	-45.0084
Video length	-0.014	-31.1608	-9.4026	-33.9187	-28.9472
Channel video count	-0.0143	-26.1214	-7.882	-28.4333	-24.2657
Opposing comment proportion	-0.0543	-15.3969	-4.6459	-16.7596	-14.3031
Party-aligned comment proportion	-0.1043	-4.8802	-1.4726	-5.3121	-4.5335
Verified account	-0.1556	-3.6068	-1.0883	-3.926	-3.3505

Table 39: The true difference between Republican and Democratic videos with regards to their normalized values for a given engagement metric as well as the ratio between the difference needed to match the observed skew and that of the true difference in mean between Republican and Democratic videos for a given engagement metric.

Topic	Anti Democrat	Anti Republican	Neutral	Pro Democrat	Pro Republican
AI, LLMs	9	1	29	4	0
Abortion and reproductive health	82	148	96	148	8
Anything outside the US or involve US foreign relations except for Israel, Gaza, Ukraine, or immigration	235	72	1217	8	40
Assassination attempt on Donald Trump	38	46	57	3	53
Biden dropping out of the presidential race	288	68	153	227	25
Climate change	33	33	64	40	2
Covid, including covid vaccines	52	29	35	7	7
Crime generally	236	141	1257	19	25
Crypto	4	5	11	0	4
Democratic National Convention (DNC)	38	6	28	43	1
Economy generally	260	147	496	133	82
Education	54	44	176	36	15
Environment generally	18	15	117	12	6
Government or politics generally	1926	1254	2466	715	609
Guns and gun control	55	38	227	35	18
Immigration	286	123	244	34	56
Israel, Gaza or Palestine, including anything about Netanyahu or Hamas	239	88	1014	13	17
LGBTQ+ issues, including transgender issues	132	61	160	48	15
Other	63	6	2561	3	7
Other public health issues	39	28	350	37	18
Other social issues, including culture war issues, labor, and other social issues that are not covered above	355	164	2082	92	61
Other technology issues	28	12	136	2	7
Other vaccines	7	0	6	1	2
Racial issues, including affirmative action and racial discrimination	299	175	408	87	23
Republican National Convention (RNC)	5	18	5	0	18
Ukraine war	77	14	135	2	15

Table 40: The number of videos of each political classification on a certain topic.

Supplementary Figures

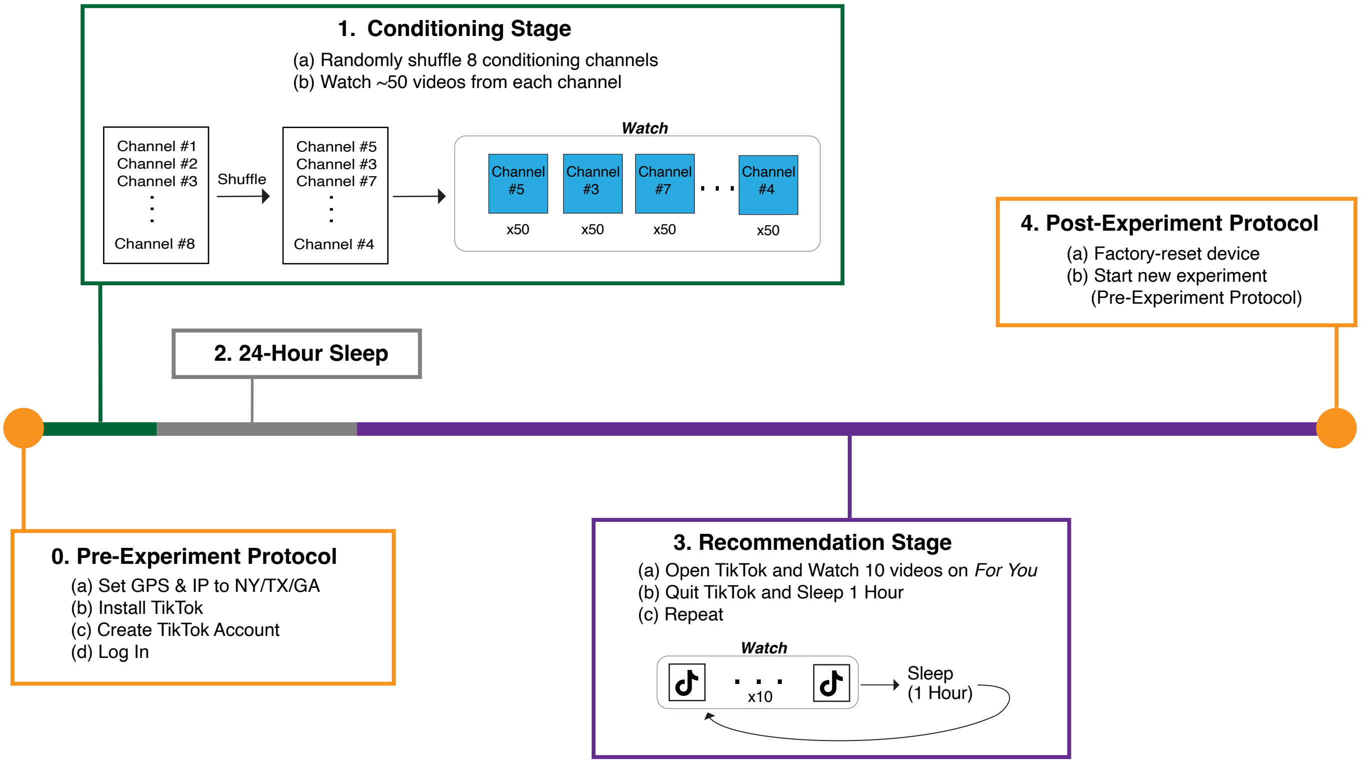

Figure 4: A device's timeline during a weekly experimental run

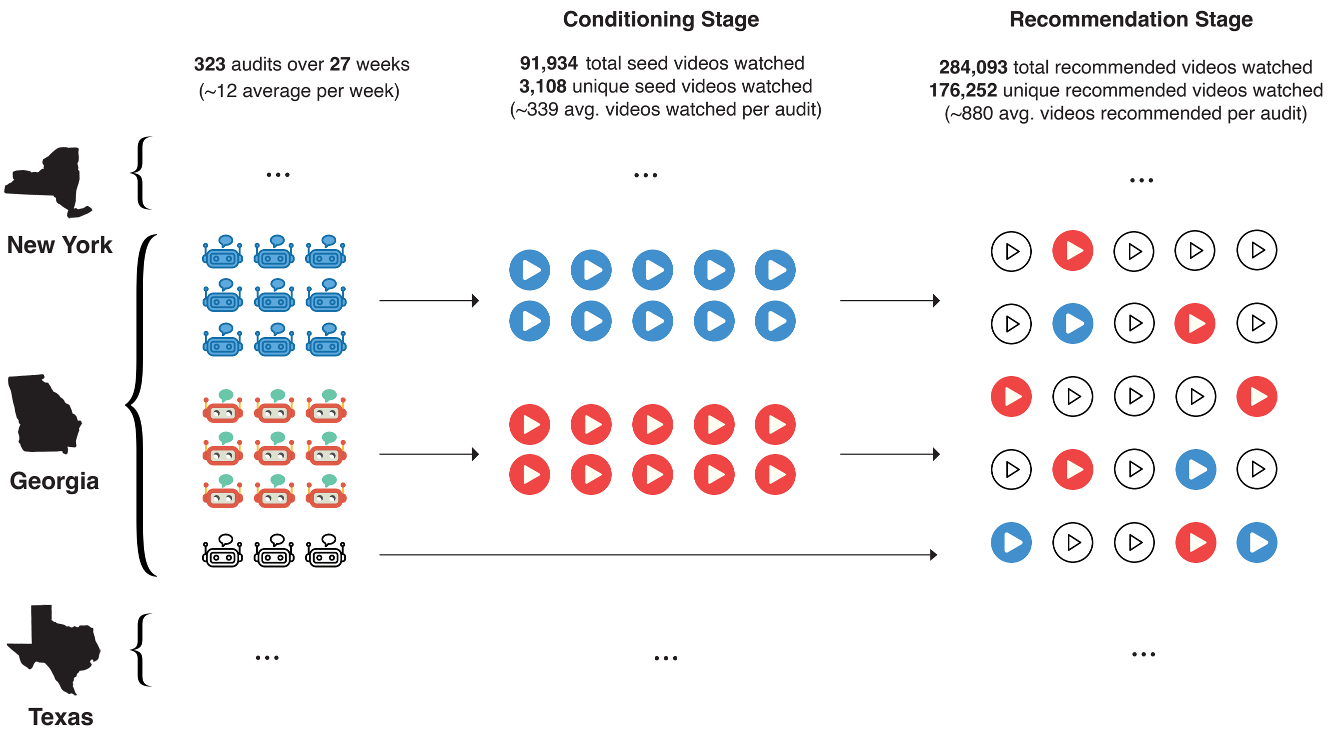

Figure 5: Visual overview of experimental setup and data collected at each stage

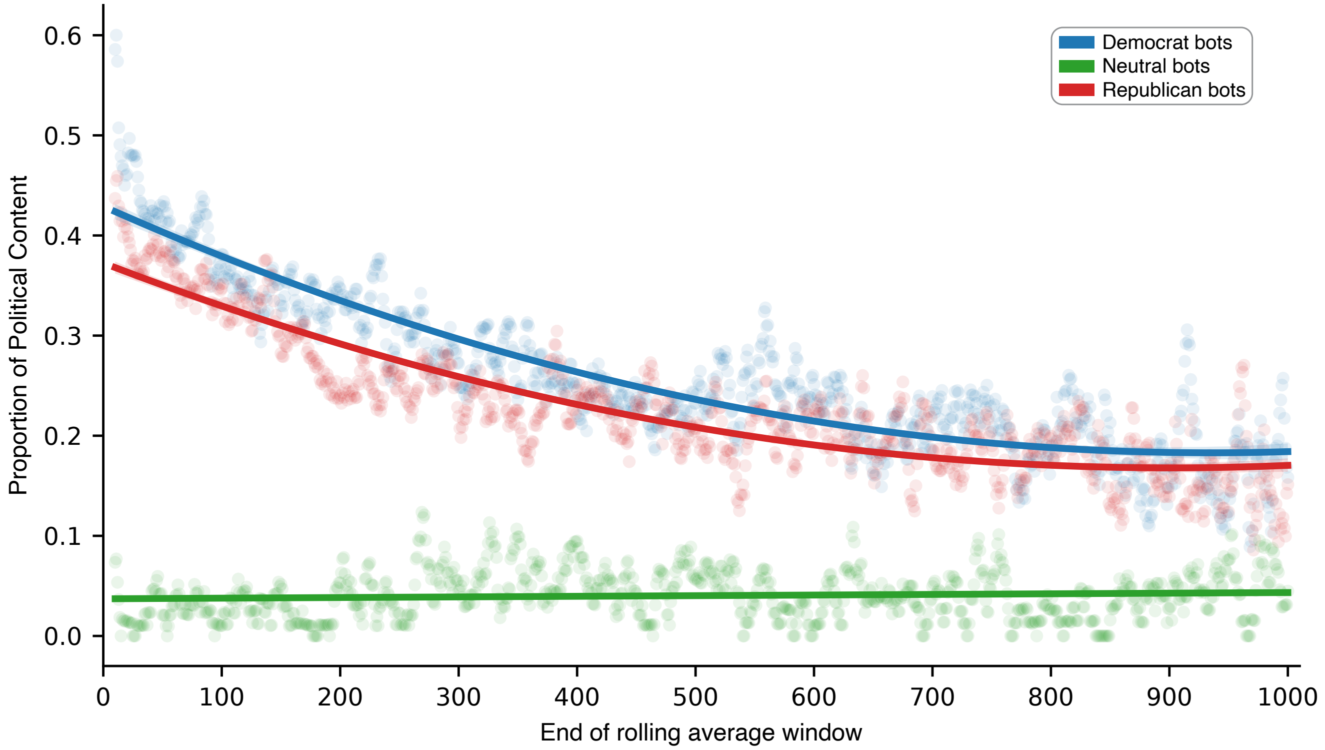

Figure 6: Rolling average of political content viewed by bots of different conditioning in 10 video windows.

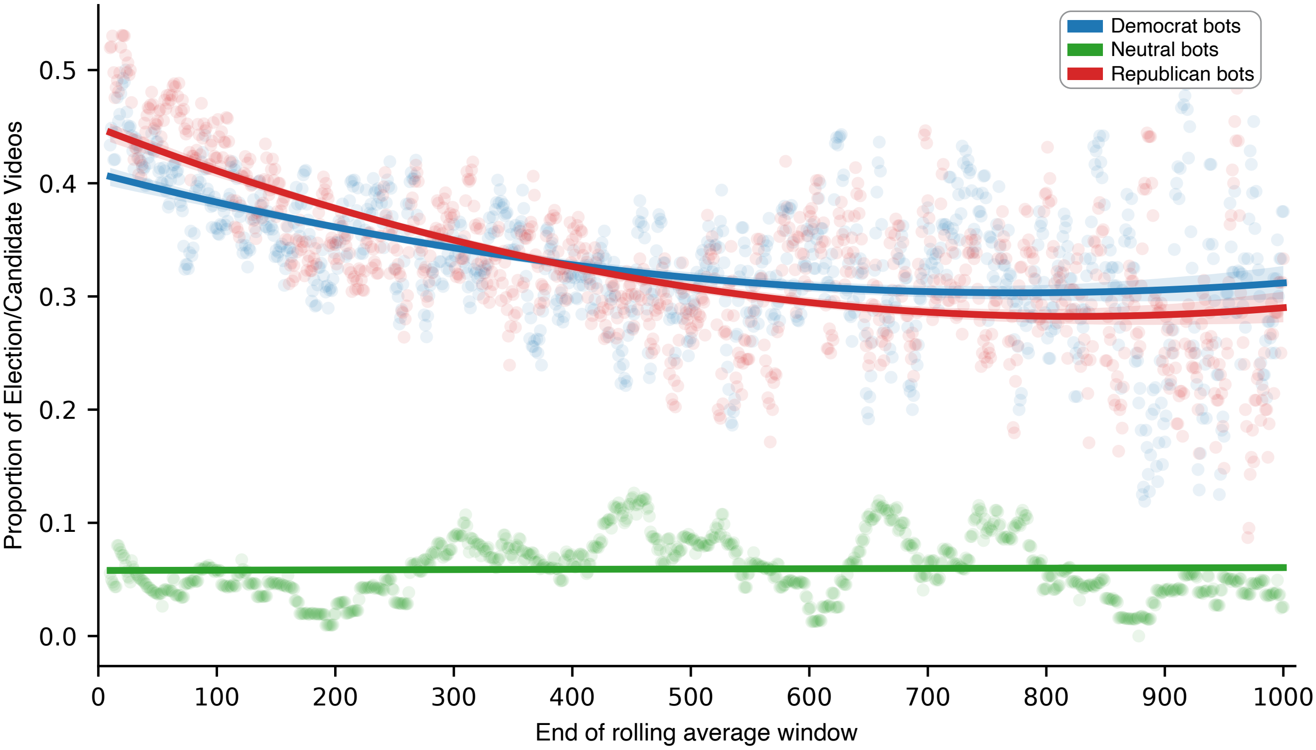

Figure 7: Rolling average of political content about the US elections or about major political candidates (out of all political content) viewed by bots of different conditioning in 10 video windows.

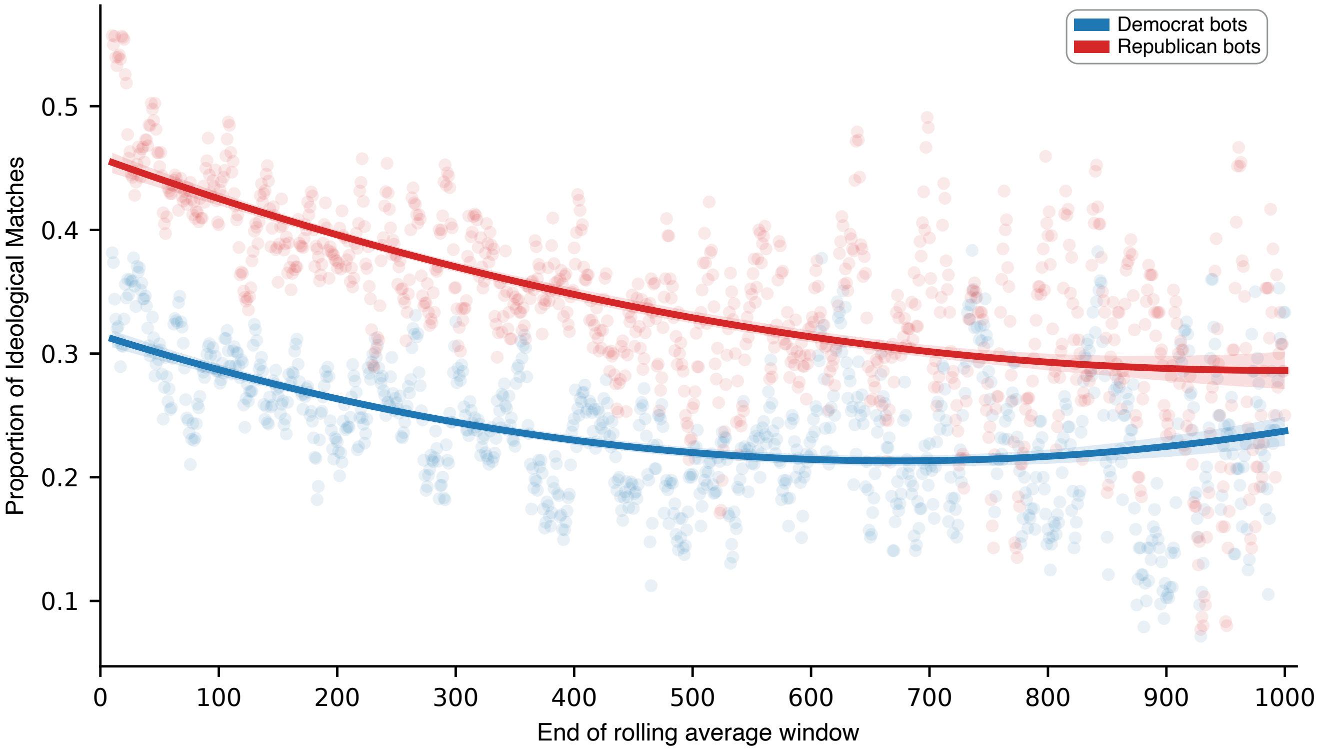

Figure 8: Rolling average of political content that is ideologically matched (out of all political content) to bots of different conditioning in 10 video windows. Pro Democrat or Anti Republican videos are ideologically matched with Democrat bots, while Pro Republican or Anti Democrat videos are ideologically matched with Republican bots.

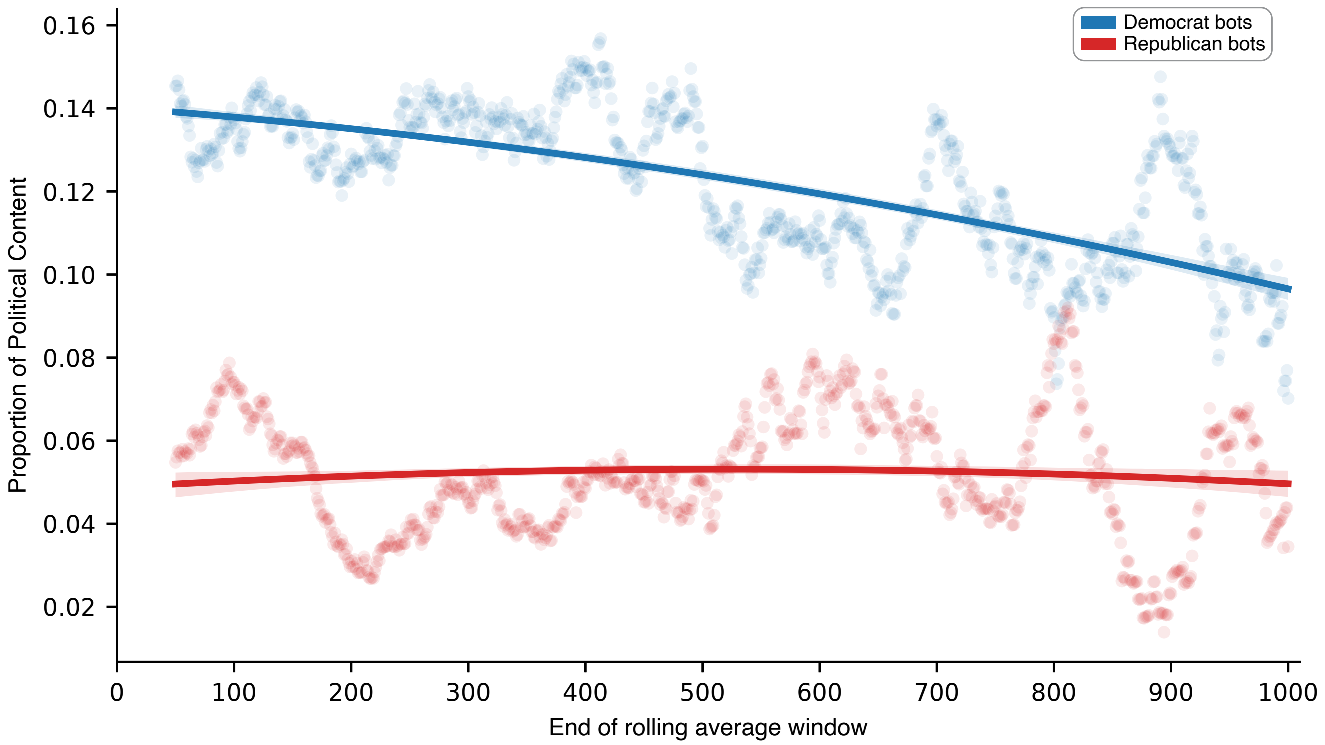

Figure 9: Rolling average of political content that is ideologically mismatched (out of all political content) to bots of different conditioning in 10 video windows. Pro Republican or Anti Democrat videos are ideologically mismatched with Democrat bots, while Pro Democrat or Anti Republican videos are ideologically mismatched with Republican bots.

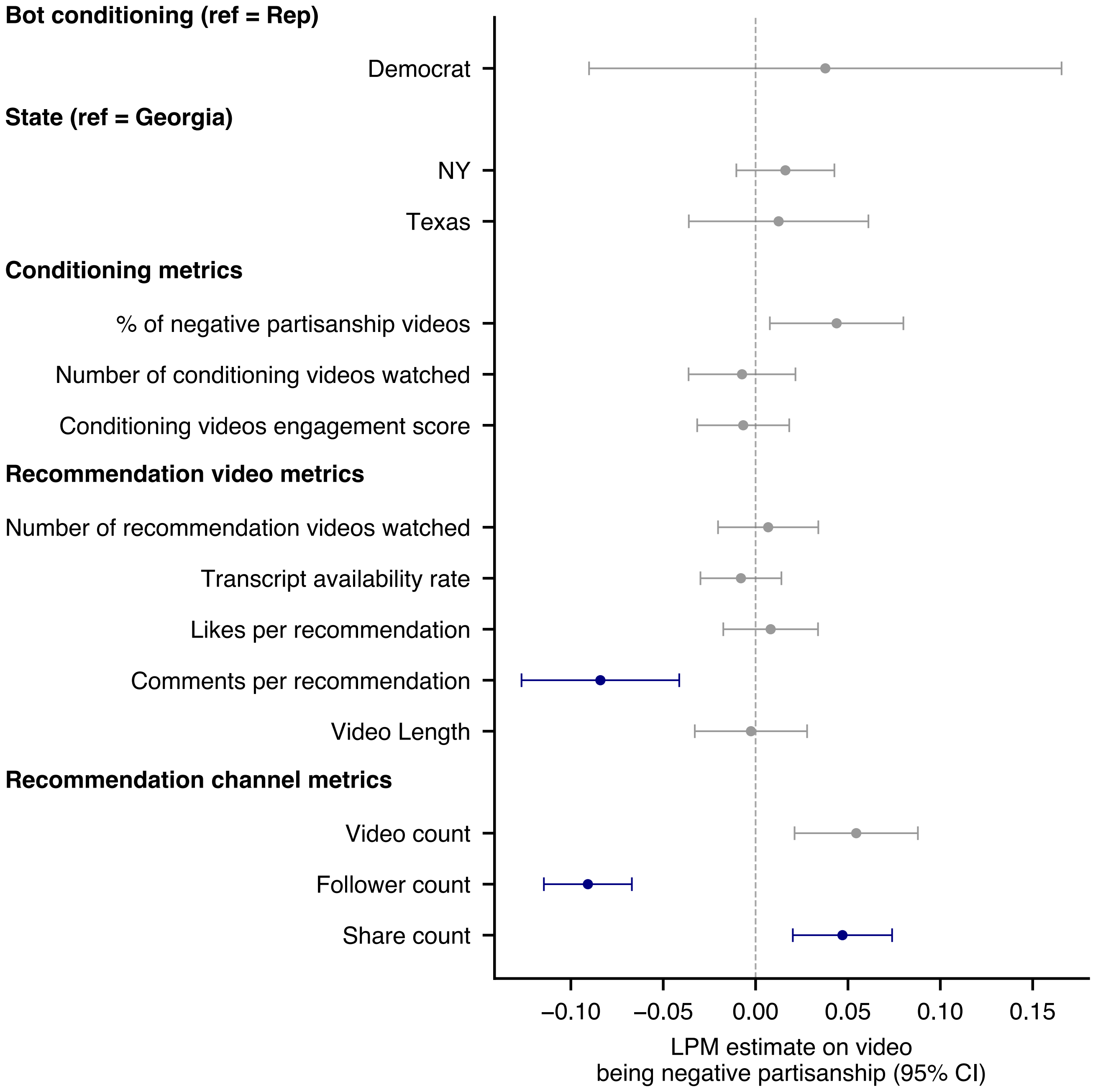

Figure 10: Linear probability model estimates on the percentage point change in the probability of a recommended video being of negative partisanship. Statistically significant coefficients after Benjamini-Hochberg multiple comparison adjustments are highlighted in navy.

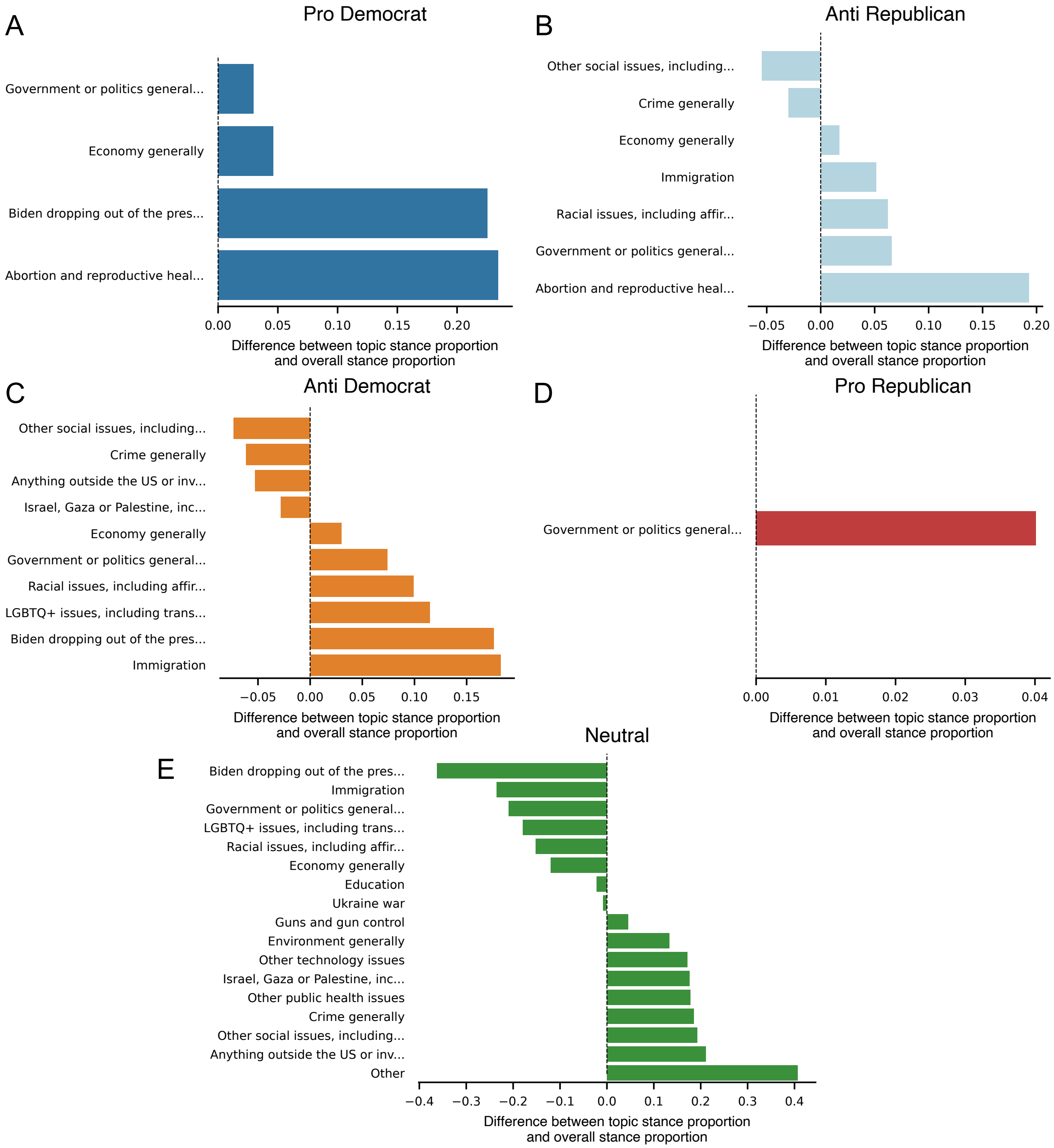

Figure 11: Of topic-stance pairs with at least 100 videos, the difference in the proportion of videos on the topic with the given stance and the proportion of videos of that stance out of all political videos. Positive values indicate a higher than average representation of the stance within a topic, while negative values lower than average representation of the stance within the topic. Plots **A**, **B**, **C**, **D**, **E** correspond to the Pro Democrat, Anti Republican, Anti Democrat, Pro Republican, and Neutral stances, respectively.

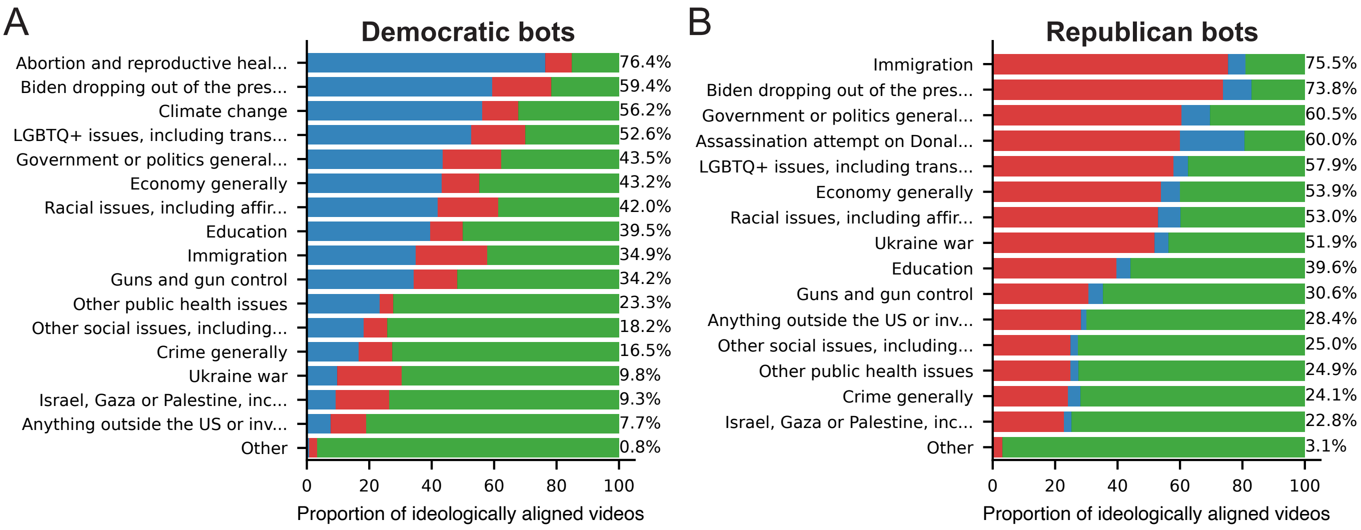

Figure 12: (**A**, **B**) The proportion of videos on a given topic which are ideologically-aligned, ideologically-opposing, or neutral, seen by Democrat- and Republican-conditioned bots, respectively. For each plot, topics are listed in descending order of ideological-alignment.

References

[1] Doyle, B. TikTok Statistics. https://wallaroomedia.com/blog/social-media/tiktok-statistics/. [Accessed 28-11-2024].

[2] TikTok CEO pledges 2 billion to protect 170 million US users. https://www.bloomberg.com/news/articles/2024-01-31/tiktok-ceo-pledges-2-billion-to-protect-170-million-us-users. [Accessed 28-11-2024].

[3] Eddy, K. 6 facts about Americans and TikTok — pewresearch.org. https://www.pewresearch.org/short-reads/2024/04/03/6-facts-about-americans-and-tiktok/. [Accessed 28-11-2024].

[4] Leppert, R. & Matsa, K. E. More Americans – especially young adults – are regularly getting news on TikTok — pewresearch.org. https://www.pewresearch.org/short-reads/2024/09/17/more-americans-regularly-get-news-on-tiktok-especially-young-adults/. [Accessed 28-11-2024].

[5] TikTok. Politics, Religion, and Culture — TikTok Advertising Policies — ads.tiktok.com. https://ads.tiktok.com/help/article/tiktok-ads-policy-politics-religion-and-culture (October, 2024). [Accessed 28-11-2024].

[6] Vinocur, N. & Haeck, P. TikTok CEO summoned to European Parliament over role in shock Romania election — politico.eu. https://www.politico.eu/article/elections-tiktok-ceo-eu-parliament-romania-election-fake-accounts-pro-russia-calin-georgescu-nato-shock-victory/. [Accessed 28-11-2024].

[7] Henly, J. Romania regulator calls for TikTok suspension amid vote interference fears — theguardian.com. https://www.theguardian.com/world/2024/nov/27/romanian-regulator-tiktok-suspended-cyber-interference-election-georgescu. [Accessed 28-11-2024].

[8] Commission, E. The EU's Digital Services Act — commission.europa.eu. https://commission.europa.eu/strategy-and-policy/priorities-2019-2024/europe-fit-digital-age/digital-services-act_en. [Accessed 28-11-2024].

[9] House, T. W. Addressing the Threat Posed by TikTok, and Taking Additional Steps to Address the National Emergency With Respect to the Information and Communications Technology and Services Supply Chain. https://www.federalregister.gov/documents/2020/08/11/2020-17699/addressing-the-threat-posed-by-tiktok-and-taking-additional-steps-to-address-the-national-emergency. [Accessed 28-11-2024].

[10] Allyn, B. U.S. Judge Halts Trump's TikTok Ban, The 2nd Court To Fully Block The Action. https://www.npr.org/2020/12/07/944039053/u-s-judge-halts-trumps-tiktok-ban-the-2nd-court-to-fully-block-the-action (2020). [Accessed 28-11-2024].

[11] Jeff Stein, D. H. & Bogage, J. Trump expected to try to halt TikTok ban, allies say. https://www.washingtonpost.com/business/2024/11/12/trump-tiktok-ban-sale/. [Accessed 28-11-2024].

[12] Maheshwari, S. & McCabe, D. Congress Passed a Bill That Could Ban TikTok. Now Comes the Hard Part. — nytimes.com. https://www.nytimes.com/2024/04/23/technology/bytedance-tiktok-ban-bill.html. [Accessed 28-11-2024].

[13] Toh, M. Jack Ma loses more than half of his wealth after criticizing Chinese regulators — CNN Business — edition.cnn.com. https://edition.cnn.com/2023/07/12/business/china-jack-ma-wealth-drop-intl-hnk/index.html. [Accessed 29-11-2024].

[14] Ye, J. China announces rules to reduce spending on video games. https://www.reuters.com/world/china/china-issues-draft-rules-online-game-management-2023-12-22/. [Accessed 29-11-2024].

[15] Hern, A. Revealed: how TikTok censors videos that do not please Beijing — theguardian.com. https://www.theguardian.com/technology/2019/sep/25/revealed-how-tiktok-censors-videos-that-do-not-please-beijing. [Accessed 29-11-2024].

[16] Harris, M. TikTok apologized for the glitch affecting the 'black lives matter' hashtag after accusations of censorship: 'We know this came at a painful time' — businessinsider.com. https://www.businessinsider.com/tiktok-apologizes-for-blm-hashtag-glitch-after-censorship-allegations-2020-6. [Accessed 29-11-2024].

[17] News, N. Trump likely to give tiktok a 90-day extension to avoid ban (2025). URL https://www.nbcnews.com/politics/donald-trump/trump-likely-give-tiktok-90-day-extension-avoid-ban-rcna188258. [Accessed: 2025-01-19].

[18] TikAPI. Tikapi: Tiktok api for developers (2025). URL https://tikapi.io/.

[19] Hosseinmardi, H. *et al.* Evaluating the scale, growth, and origins of right-wing echo chambers on youtube. *arXiv preprint arXiv:2011.12843* (2020).

[20] Ledwich, M. & Zaitsev, A. Algorithmic extremism: Examining youtube's rabbit hole of radicalization. *arXiv preprint arXiv:1912.11211* (2019).

[21] Stocking, G. *et al.* News influencers on TikTok — pewresearch.org. https://www.pewresearch.org/journalism/2024/11/18/news-influencers-on-tiktok/ (2024). [Accessed 11-06-2025].

[22] Hosseinmardi, H. *et al.* Causally estimating the effect of YouTube's recommender system using counterfactual bots. *Proceedings of the National Academy of Sciences* **121**, e2313377121 (2024).

[23] Stocking, G. America's News Influencers — pewresearch.org. https://www.pewresearch.org/journalism/2024/11/18/americas-news-influencers/. [Accessed 02-01-2025].
