# Supplementary material for: Systematic partisan content skews in TikTok during the 2024 US elections
Source: Nature. 2026 May 6;654(8120):1004–11. doi: 10.1038/s41586-026-10447-1 (PMC13293873; doi:10.1038/s41586-026-10447-1)
Supplement: Supplementary file 2 — Reporting Summary [file 41586_2026_10447_MOESM2_ESM.pdf]

nature portfolio

Corresponding author(s): Talal Rahwan, Yasir Zaki

Last updated by author(s): Mar 8, 2026

Reporting Summary

Nature Portfolio wishes to improve the reproducibility of the work that we publish. This form provides structure for consistency and transparency in reporting. For further information on Nature Portfolio policies, see our Editorial Policies and the Editorial Policy Checklist.

Statistics

For all statistical analyses, confirm that the following items are present in the figure legend, table legend, main text, or Methods section.

n/a	Confirmed	
☐	☒	The exact sample size (*n*) for each experimental group/condition, given as a discrete number and unit of measurement
☐	☒	A statement on whether measurements were taken from distinct samples or whether the same sample was measured repeatedly
☐	☒	The statistical test(s) used AND whether they are one- or two-sided<br>*Only common tests should be described solely by name; describe more complex techniques in the Methods section.*
☐	☒	A description of all covariates tested
☐	☒	A description of any assumptions or corrections, such as tests of normality and adjustment for multiple comparisons
☐	☒	A full description of the statistical parameters including central tendency (e.g. means) or other basic estimates (e.g. regression coefficient) AND variation (e.g. standard deviation) or associated estimates of uncertainty (e.g. confidence intervals)
☐	☒	For null hypothesis testing, the test statistic (e.g. *F*, *t*, *r*) with confidence intervals, effect sizes, degrees of freedom and *P* value noted<br>*Give P values as exact values whenever suitable.*
☒	☐	For Bayesian analysis, information on the choice of priors and Markov chain Monte Carlo settings
☒	☐	For hierarchical and complex designs, identification of the appropriate level for tests and full reporting of outcomes
☒	☐	Estimates of effect sizes (e.g. Cohen's *d*, Pearson's *r*), indicating how they were calculated

Our web collection on statistics for biologists contains articles on many of the points above.

Software and code

Policy information about availability of computer code

Data collection

We created a suite of scraping scripts in order to simulate user behaviour on TikTok. This required us to dedicate an Android smartphone, namely Samsung Galaxy A34 5G, to each of the 21 accounts created every week. Before each experiment, we control device geo-location across three target states using a combined approach of GPS mocking and VPN tunneling. Specifically, we employed AnyTo for GPS coordinate spoofing, setting New York bots to <40.7308, -73.9976> in Manhattan, New York City, Texas bots to <33.148, -96.638> in Collin County, and Georgia bots to <33.961, -84.537> in Cobb County. These specific locations were chosen as counties which voted strongly Democrat, Republican, or was a close call in the 2020 U.S. presidential elections, respectively.

Furthermore, to align each bot's network identity with the intended state's geo-location, we tunnel each phone's public IP address to one of three custom VPN servers we hosted on third-party cloud providers. We avoided commercial VPN services to minimize the risk of TikTok identifying the IPs as virtual. We install TikTok from Google PlayStore only after each phone's GPS and IP address had been appropriately modified. At the conclusion of a weekly experiment, we factory-reset every phone before beginning the next round of experiment. This step ensures that any TikTok-related cache is cleared and does not influence the subsequent experiments conducted on the same phones. Finally, all phones operated on Android 13 which re-randomizes the MAC address every 24 hours, precluding the possibility of TikTok's device-level tracking or bot detection throughout a weekly experiment.

With regards to the Survey conducted, participants were recruited via Prolific.

Data analysis

All data analysis was done using Python 3.13.1. The code needed to replicate our findings can be found at https://doi.org/10.5281/zenodo.18907781

For manuscripts utilizing custom algorithms or software that are central to the research but not yet described in published literature, software must be made available to editors and reviewers. We strongly encourage code deposition in a community repository (e.g. GitHub). See the Nature Portfolio guidelines for submitting code & software for further information.

Data

Policy information about availability of data

All manuscripts must include a data availability statement. This statement should provide the following information, where applicable:

- Accession codes, unique identifiers, or web links for publicly available datasets
- A description of any restrictions on data availability
- For clinical datasets or third party data, please ensure that the statement adheres to our policy

The full dataset of political videos on TikTok analyzed in this study can be found at https://doi.org/10.5281/zenodo.18907781

Research involving human participants, their data, or biological material

Policy information about studies with human participants or human data. See also policy information about sex, gender (identity/presentation), and sexual orientation and race, ethnicity and racism.

Reporting on sex and gender	In the Participants Demographics table of the Supplementary, we group sex along with age and race under the header Demographics. In the remainder of the paper, we do not mention gender or sex explicitly.
Reporting on race, ethnicity, or other socially relevant groupings	Our study focuses on political ideology, which may be understood as a socially relevant grouping. In the context of our survey, this was based on self-reported ideology by the participants. The options given to the participants were taken from the CES 2024.
Population characteristics	Participants (N = 1,008) were recruited via Prolific from U.S.-based active TikTok users; the sample was not nationally representative and skewed Republican (54.8% Republican, 31.4% Democratic, 13.8% Independent), with 54.3% female, 44.5% male, and 1.2% non-binary respondents; 64.1% identified as White and 27.4% as Black; 82.2% held at least a bachelor's degree; and the largest age group was 25–34 years old (37.0%).
Recruitment	We administered a pre-registered survey with a sample of 1,008 U.S.-based TikTok users to assess whether individuals had noticed changes to the content of their TikTok feeds, particularly political content, over the past year. The survey was preregistered on OSF (\url{https://osf.io/udywb/}) and was deemed exempt by the authors' institutional review board (IRB Protocol Number: HRPP-2025-69). Participants were recruited via convenience sampling on Prolific. The conditions for inclusion into the target pool included being located in the United States, being an active TikTok user, and being a native English speaker.
Ethics oversight	The study was approved by the author's institutional review board. IRB Protocol Number: HRPP-2025-69

Note that full information on the approval of the study protocol must also be provided in the manuscript.

Field-specific reporting

Please select the one below that is the best fit for your research. If you are not sure, read the appropriate sections before making your selection.

☐ Life sciences ☒ Behavioural & social sciences ☐ Ecological, evolutionary & environmental sciences

For a reference copy of the document with all sections, see nature.com/documents/nr-reporting-summary-flat.pdf

Behavioural & social sciences study design

All studies must disclose on these points even when the disclosure is negative.

Study description	The survey used a mixed-methods design and consisted of two parts: (1) a series of qualitative open-ended text entry questions, and (2) a series of quantitative structured, scale-based questions. Open-ended items asked participants whether they had noticed any changes to the content on their TikTok feed in general, any changes to political content specifically, and whether the tone of political content had become more positive or negative. Responses to these questions were manually coded by the first author to determine whether participants explicitly referenced changes to political content and, if so, whether they described seeing more Republican-aligned or Democratic-aligned content.<br><br>Structured questions asked participants to rate, on a 0–10 scale, the extent to which their feed had shifted toward Democratic or Republican content, become more positive or negative in tone, or featured more political content they agreed or disagreed with.
Research sample	The survey was conducted with a sample of 1,008 U.S.-based TikTok users. Our inclusion criteria included that the participant was present in the United States in 2024, was an active TikTok user, and spoke English, given that our study focuses on English-language videos shown to US users in 2024. Participants (N = 1,008) were recruited via Prolific from U.S.-based active TikTok users; the sample was not nationally representative and skewed Republican (54.8% Republican, 31.4% Democratic, 13.8% Independent), with 54.3% female, 44.5% male, and 1.2% non-binary respondents; 64.1% identified as White and 27.4% as Black; 82.2% held at least a bachelor's degree; and the largest age group was 25–34 years old (37.0%). This sample is not representative of the general United States population, and it is unclear whether it is representative of the population of TikTok users.

Sampling strategy	As this analysis was exploratory, we considered a sample size of 1000 participants. These participants were recruited via Prolific. Convenience sampling was used to recruit participants on the platform.
Data collection	Participants conducted the survey online via their tablets or laptops using Qualtrics (the researcher was not present while the participants conducted the survey). There were no experimental conditions in this survey, and thus neither the participants nor the researchers were blind to any conditions.
Timing	The survey was conducted on April 17th, 2025.
Data exclusions	Two participants which indicated that they do not consent to their participants in the study were excluded.
Non-participation	All consenting participants completed the full questionnaire.
Randomization	Participants were not allocated into groups and no randomization was conducted.

Reporting for specific materials, systems and methods

We require information from authors about some types of materials, experimental systems and methods used in many studies. Here, indicate whether each material, system or method listed is relevant to your study. If you are not sure if a list item applies to your research, read the appropriate section before selecting a response.

Materials & experimental systems

n/a	Involved in the study	
☒	☐	Antibodies
☒	☐	Eukaryotic cell lines
☒	☐	Palaeontology and archaeology
☒	☐	Animals and other organisms
☒	☐	Clinical data
☒	☐	Dual use research of concern
☒	☐	Plants

Methods

n/a	Involved in the study	
☒	☐	ChIP-seq
☒	☐	Flow cytometry
☒	☐	MRI-based neuroimaging

Plants

Seed stocks	*Report on the source of all seed stocks or other plant material used. If applicable, state the seed stock centre and catalogue number. If plant specimens were collected from the field, describe the collection location, date and sampling procedures.*
Novel plant genotypes	*Describe the methods by which all novel plant genotypes were produced. This includes those generated by transgenic approaches, gene editing, chemical/radiation-based mutagenesis and hybridization. For transgenic lines, describe the transformation method, the number of independent lines analyzed and the generation upon which experiments were performed. For gene-edited lines, describe the editor used, the endogenous sequence targeted for editing, the targeting guide RNA sequence (if applicable) and how the editor was applied.*
Authentication	*Describe any authentication procedures for each seed stock used or novel genotype generated. Describe any experiments used to assess the effect of a mutation and, where applicable, how potential secondary effects (e.g. second site T-DNA insertions, mosiacism, off-target gene editing) were examined.*
