# Supplementary material for: Systematic partisan content skews in TikTok during the 2024 US elections
Source: Nature. 2026 May 6;654(8120):1004–11. doi: 10.1038/s41586-026-10447-1 (PMC13293873; doi:10.1038/s41586-026-10447-1)
Supplement: Supplementary file 3 — Peer Review File [file 41586_2026_10447_MOESM3_ESM.pdf]

nature portfolio

Peer Review File

Systematic partisan content skews in TikTok during the 2024 U.S. elections

Corresponding Author: Professor Yasir Zaki

Any redactions in this file are there to maintain patient confidentiality, the confidentiality of unpublished data, or to remove third-party material.

This file contains all reviewer reports in order by version, followed by all author rebuttals in order by version.

Version 1:

Reviewer comments:

Referee #1

(Remarks to the Author)
Key results: This study is a unique piece of evidence excellently documenting the power of filtering algorithms on the platform TikTok when it comes to politics. The comprehensive steps authors took to ensure the validity of their results is impressive. The key outstanding features, in my view, are 1) the combination of observational components (the audits) combined with content analaytical techniques (to identify stances) and additional analyses to exclude engagement-based asymmetries as confounders. 2) The longitudinal nature of the study allows a dynamic observation of algorithmic curation and its reinforcement learning based on user preferences. 3) The application of the study's interest in an electoral context helps uncovering TikTok's potential power on election outcomes, at least shaping the agenda and tone of content individual citizens receive.
It is striking that Republican accounts receive 11.5% more congenial content than Democratic accounts, emphasizing an asymmetry in the ideology-driven algorithmic curation of content on TikTok. This result has strong implications for content regulation policies.
The quality of this work is uniquely high and I recommend the editor's team to consider this piece for publication, provided that authors are willing to address the points I am raising below which, I think, could help increasing the ultimate contribution of this work.

Validity: I do not identify any key flaws the impede publication.

Originality and significance: These results have high significance for several disciplines, inter alia, political science, communication studies, political psychology, sociology, ethics as well as legal sciences as these findings clarify an imbalance in the distribution of ideology-based content, having users not being aware of this, leading to a inhbition of users' informational self-determination. Would Democrats keep on using this platform after being educated that their content feed does not equate their Republication counterparts in terms of ideological constitution?

The multi-methodological approach of this piece and its rigor is a clear strength. Still, there are a series of observations that I would like to see addressed:
1) There are a lot of numbers thrown around in the experimental setup und methodological section (audits, runs, videos, etc). While their interconnection might be clear for authors, it would be helpful to have a figure that indicates how many weeks, how many audits, how many videos, how many experiment runs, … were conducted and whether and how these numbers are interrelated. While Figure 1 is helpful in disentangling the experimental design, another one (maybe in the supplementary material) could help to comprehend the massive data set you rely this study on.
2) Authors justify their selection of States as assignment as likely Democrat, Republican or Swing State. Still, it is unclear why these three were chosen. Is there any rationale behind these three states? Do you expect that results are similar in other states that fall into the same category?
3) You write: „This process yielded 56 Democratic-aligned channels and 75 Republican-aligned channels, which we manually validated following best practices on channel-level classification tasks" (p. 9) -> Is there a chance to compare this distribution with other numbers to see whether it resembles the actual prevalence of political channels on Tik Tok?
4) What is the exact difference between symmetric and asymmetric polarization? In the explanation, they both read quite

similar. It seems to me that in the asymmetric form the algorithm considers other users, too? Could you please explain?

Conclusions: Despite the excellence and high level of quality that is provided from a theoretical and methodological point of view, the conclusions drawn need to be refined:
5) You state: „we contribute to ongoing debates about whether algorithmic bias or user behavior is more responsible for the polarization observed on social media platforms“ (p. 3) But you are not studying „polarization“ per se. Your study presents the distribution of political content in the network of TikTok. This evidence does not allow any conclusions about polarization and the ultimate link between social media use and polarization is yet to be understood. The state-of-the-art is mixed and I would not take it as a given that social media (and human- or algorithm-based decisions) are responsible for a polarized system.
6) In the presentation of the survey results, you state the Republicans „were more likely to characterize the political content on their feed as positive, optimistic, agreeable, and pro-Trump“(p. 21). The fact that Republicans perceive to see more pro-Trump content the Democrats is not surprising. The key question is whether they perceived to access more pro-Trump content than Democrats perceived to access pro-Harris content? This would just be an interesting addition, since we all know that these perceptions may not fully reflect reality. Still, it would be interesting to know whether the imbalance is also resembled in users‘ perception.
7) You write „these findings are consistent with growing evidence that conservative users in particular may be more likely to occupy ideologically homogeneous media environments“ (p. 24). This interpretation could be clarified. I am not sure whether it is a deliberate occupation by Republicans or whether it is just that the algorithms play in their hands?
8) The interpretation of the results could be expanded as these results have a high significance from different perspectives. Maybe, the following questions could help extending the discussion and making the contribution of this piece clearer
a) Do these results clearly point to an ideological bias in the algorithmic filtering?
b) If yes, why so? Who is or should be responsible for this?
c) How are these results comparable with previous works on the ideological bias of algorithmic curation on social media?
d) Is TikTok unique in this regard? Is it because the algorithm is so powerful compared to other platforms?
e) What could effects of this imbalance in the distribution of political content be? How may it shape public opinion formation? How may it shape voting decisions? Is there any group that may be very susceptible in this context?
f) Does this observation indicate a violation of human rights? How would the EU Digital Services Act interpret these results? Is is transferrable to the U.S. system?

I really enjoyed reading this research and wish authors the best of luck.

(Remarks on code availability)

Referee #2

(Remarks to the Author)
The submitted manuscript seeks to measure partisan bias in TikTok's recommendation of political content to Democratic- and Republican-leaning accounts using "sock puppet" accounts that are created and seeded by viewing aligned political content. The results are interesting and novel -- as the authors note, TikTok is less well understood than other prominent social media platforms and the data collection approach here is ambitious and impressive. However, their design cannot rule out alternative explanations for the asymmetries in recommendation patterns by partisanship that they observe. We therefore regretfully recommend that the article be declined. Our comments follow:

1. Why do the authors think this is happening?
The manuscript offers no convincing explanation for the observed partisan asymmetries in content recommendations. Is TikTok intentionally promoting more pro-Republican and anti-Democrat content or is it somehow responding to the preferences and behavior of users on the platform? The authors consider several possibilities but find the evidence is not consistent with each of them. This creates a gaping theoretical/interpretive hole.

2. Alternative explanations for asymmetry
Most importantly, we are concerned that the central finding of the paper - that Republican-seeded accounts on TikTok receive more congenial recommendations than do Democrat-seeded accounts - could be an artifact of the research design or other features of the platform rather than a true partisan asymmetry in algorithmic recommendation behavior.

Most notably, eight of the twelve Democratic-leaning accounts used for seeding the Democratic sock puppet accounts are official Democratic Party sources or Democratic members of Congress (e.g., Ro Khanna, Bernie Sanders, and Alexandria Ocasio-Cortez), whereas all twelve of the Republican-leaning accounts used for seeding the Republican sock puppet accounts are right-wing content creators who are not formally affiliated with the Republican Party (Tucker Carlson, Ben Shapiro, and various lesser-known accounts). The differences in the videos watched by the Democratic- and Republican-seeded accounts could easily reflect asymmetries between these two sets of videos that are not a reflection of partisanship. For instance, the observed differences in recommendation patterns could reflect how the algorithm responds to differences in ideological extremism or preferences for institutional versus independent sources rather than being an asymmetry in how it recommends content to Democratic- and Republican-leaning users.

Consider, for instance, how TikTok describes its algorithm as reported by Axios (https://www.axios.com/2020/09/10/inside-tiktoks-killer-algorithm):

"The algorithm identifies similar videos to those that have engaged a user based on video information, which could include details like captions, hashtags or sounds. Recommendations also take into account user device and account settings, which include data like language preference, country setting, and device type."

"Once TikTok collects enough data about the user, the app is able to map a user's preferences in relation to similar users and group them into 'clusters.' Simultaneously, it also groups videos into 'clusters' based on similar themes, like 'basketball' or 'bunnies.'"

"Using machine learning, the algorithm serves videos to users based on their proximity to other clusters of users and content that they like."

There’s no reason to necessarily think that TikTok is grouping users into “Democrat” and “Republican” clusters and making recommendations to them asymmetrically. One could easily imagine instead the accounts being assigned who watch videos from members of Congress being grouped into a cluster that is broadly interested in politics and receives recommendations that match that preference, while those who watch videos from conservative content creators might be clustered with people who have different viewing habits.

In general, the paper lacks theoretical depth on different types of algorithmic biases or recommendation patterns and how they might help explain the observed results. It would also be helpful to consider whether and how bias is appropriately defined in this context.

3. Data limitations
The authors also face two key data limitations that make it extremely difficult to make convincing inferences about the quantities they wish to estimate. These limitations are of course not their fault, but still raise questions about the claims that the authors offer.

The first is that they do not have access to dwell time, which is reportedly the key video-level metric of engagement that TikTok’s algorithm seeks to optimize. They instead use accessible “video-level metrics such as a video’s likes, likes per recommendation, comments, comments per recommendation, plays, and share count.” These are surely correlated with dwell time but they are not the same. The sensitivity analysis seems to help address this concern (though see below), but the absence of dwell time statistics means we can’t rule it out as an explanation for the observed differences in recommendations. In particular, the authors dismiss an argument about asymmetric homophily using (insignificant differences in) comments on videos (see below) but dwell time is surely the most relevant metric here.

In addition, the authors’ video-level analyses are conducted only among the videos that were recommended to the sock puppet accounts and not the true population of posts or a random sample of it. As a result, it is not possible to rule out the possibility that the observed asymmetries reflect an underlying imbalance in the supply of partisan content on the platform. This problem applies to every video-level datapoint (including the comments analysis, the transcripts, etc.).

4. Clustering
The reported results treat each recommended video as independent but of course the recommendations are surely correlated within bot and within a given time period. We recommend implementing two-way clustering by bot and week in all statistical analyses.

5. Discrepancies in pre-registration and tables/figures
-“We also exclude pairs of bots that watched fewer than 150 videos each during the recommendation stage to allow for a sufficient number of political videos to be recommended, bearing in mind that not all recommendations are political in nature” (page 6 of paper) vs. “ We will exclude any bot instances that do not meet the 500 recommended video threshold due to data collection errors.” (preregistration).
-The preregistration states that “The first hypothesis is that there will be significant differences between the recommendation rates of partisan political videos made to the bots who are located (via VPN) in projected republican states and those in projected democratic states” but the paper does not formally test for differences between states.
-No code is provided to generate Figures 5 or 7 or the tables.
-For Figure 2H (page 12), why are log odds centered at 1? Shouldn’t be at 0?

6. Other missing data issues
Sensitivity analyses on transcript availability and bot data missingness would be helpful as both raise selection bias concerns despite transcript availability seemingly not being associated with political leaning.

7. Other points:
-How much should we worry about multiple comparisons problems here? Should the authors be using a false discovery rate correction?
-Non-statistically significant differences in the proportion of Republican comments on Democrat posts and vice-versa are reported as “meaningful”. The authors may be able to improve precision by increasing the sample size from 200 comments.
-The sock puppet accounts are set to a random age in the range 22-24. Given that age is likely a factor in TikTok’s recommendation algorithm, this narrow and specific range limits the general interpretation of the findings. The authors should clarify this limitation and avoid overgeneralizing.
-The authors put a lot of effort into testing for state-based differences but end up finding few differences by state. They should make this point more clearly in the main text and explain to the reader when and why they pool their data versus separately

analyzing data by state. It would be helpful to specifically justify why the neutral Georgia bots are the relevant point of comparison for the partisan bots.

(Remarks on code availability)

Referee #3

(Remarks to the Author)
I co-reviewed this manuscript with one of the reviewers who provided the listed reports.

(Remarks on code availability)

Referee #5

(Remarks to the Author)
In “TikTok's recommendations skewed towards Republican content during the 2024 U.S. presidential race,” the authors report the results of an ambitious study of partisan skew in TikTok's recommendation algorithm. The study is primarily comprised of a large-scale experiment in which hundreds of sock puppet accounts (i.e., bots) are seeded by watching a set of videos with partisan skew (except for a small number of 'Neutral' accounts in the control condition). The authors then collect and analyze information about the videos that were subsequently recommended by TikTok's algorithm. They find a partisan skew in these recommendations, whereby accounts seeded with Republican content more consistently received more partisan content than those seeded with Democratic content. Moreover, in a result that is currently very much undersold in the paper, they find that this effect primarily originates from content that facilitates negative partisanship – that is, Republicans see more anti-Democrat content than Democrats see anti-Republican content, with positive partisanship varying little across the two partisan groups.

The study also includes several other analyses, including models that attempt (and ultimately fail) to explain the ideological skew from engagement metrics, topic models of the recommended content, an 'exploratory' misinformation analysis, and an original survey of TikTok users that aims to measure their perceptions of how political/partisan is the content in their feeds. Unfortunately, none of these other analyses are motivated at all in the front end of the paper (i.e., in the introduction or literature review), which makes for a difficult read. That said, there is a lot on the whole to like about the paper, from the experimental setup to the thorough discussion of the results, but the organization of the paper currently requires a lot of restructuring before it is published in a journal such as Nature.

One of the most striking missteps of the paper is the framing of the study as testing the effect of user behavior vs. algorithmic curation. While this is no doubt an interesting puzzle, and one that is exceedingly difficult to provide a comprehensive answer to, the authors simply do not (and, given the experimental setup, cannot) provide such an answer. This primary reason for this is that the experimental setup seeds each of the sock puppet accounts with partisan content, which is very much user behavior, so that any downstream effects on algorithmic recommendations simply cannot be assumed as being independent of user behavior. Moreover, it is unclear what content was used to seed these accounts as Republican or Democrat (vs. Neutral), so that the downstream ideological skew does not clearly have something to do with the algorithm but could instead results from differences in how ideologically extreme or centrist the initially seeded content is (more on this below).

As such, while the data collection and analysis efforts of the team are very impressive, the motivation for the study falls significantly short. I do not believe that this is a paper about user behavior vs. algorithmic swaying but rather a paper about negative partisanship in algorithmic recommendations – and to a lesser extent, a paper on ideological skew, if the authors can make a convincing case that this ideological skew does not result from a skew in how ideologically extreme the seeded content is. If, for example, the content seeded for Republican sock puppet accounts was more ideological extreme, or more likely to feature negative partisanship, than content seeded for Democratic sock puppet accounts, then it would seem plausible that the downstream effects for algorithmic recommendations originate from this initial seeding asymmetry and not something about the algorithm (aside from the its tendency to recommend more of what the user has already viewed).

Addressing these points would require substantial revisions to current the manuscript, and especially the frontend sections. However, given the unique and interesting data that the authors have collected, I would be happy to review a revised manuscript that addresses these points. Below, I include several other points that I hope will help the authors as they continue making revisions to the manuscript.

1) The work cited by the authors that argues online news consumption is more diverse than offline news consumption is over 10 years old. Given the changes we have seen to digital platforms and the drifting of systems more generally it would be nice to have some more recent evidence or to cite additional evidence that supports this general point.

2) I am confused about why the authors suggest that the 'weak tie phenomenon' does not happen on TikTok. If weak ties introduce users to more diverse content, wouldn't strangers have a high chance of doing the same? Or is there a different

weak tie phenomenon that the authors are referring to?

3) It is not clear why the authors spend so much time discussing the recent TikTok ban. While I understand that this is the platform they are using for data collection, the whole section has little to do with the research they are conducting and does not appear to provide any necessary context for understanding the method or results.

4) The decision to have 7 experimental conditions, instead of 9 experimental conditions, is odd. While Dem-seeded and Rep-seeded accounts were allocated to the different states (NY, TX, and GA), Neutral-seeded accounts were only allocated to GA but not NY or TX. So, if one wants to asses the effect of partisanship for a NY-Dem account, the baseline would be GA-Neutral and not NY-Neutral, which obfuscates whether the difference is the state or partisan alignment. It seems useful to have NY-Neutral and TX-Neutral accounts to assess whether differences are due to the state or partisanship of the account. Some of this could be circumvented by leveraging GA-Dem to pick up the (Dem specific) state effect, but to get at the (NY specific) party effect we would need to assume that the difference between GA-Neutral and GA-Dem is the same as NY-Neutral and NY-Dem (since we cannot explicitly measure the latter). Why did the authors not include a control group for each state? In general, it is better to compare two treatments to a control rather than to one another.

5) One concern I mentioned above is the selection of initial content that was seeded. In particular, one would expect an ideological skew in algorithmic recommendations if left-leaning seeded content was centrist, on average, while right-leaning seeded content was extreme towards the right. Can the authors speak to the ideological differences of the seeded videos, as well as the extent that they feature positive vs. negative partisanship cues? One additional difficulty here is that right-leaning content may just be more extreme than left-leaning content, on average, and so the results are an artifact of this initial skew (rather than the alternative hypothesis, which is that the TikTok algorithm sways right-leaning users more to the right more than left-leaning accounts to the left). Of course, ultimately this conflates exactly what the authors are claiming to disentangle – the decisions of users (and how extreme is the initial content they consume) vs. algorithmic swaying.

6) My major takeaway from the section on negative partisanship (and especially Figure 4) is that the source of the ideological skew identified in the previous section is entirely negative, not positive, partisanship. In particular, Pro-Democrat content for Democrats was just as likely as Pro-Republican content for Republicans. However, Anti-Democrat content for Republicans was MUCH more likely than Anti-Republican content was for Democrats. This seems like a major result, and yet it is currently difficult to decipher from the section on negative partisanship (I only realized it from studying Figure 4). I believe this finding likely belongs in the title of the paper and should be discussed much earlier and more thoroughly in the frontend (intro and lit review) sections. There is existing work on e.g., outgroup animosity (https://www.pnas.org/doi/10.1073/pnas.2024292118) and negativity (https://www.nature.com/articles/s41562-023-01538-4) that the authors can leverage here.

7) If the authors are planning to include the analyses on topic modeling, misinformation, and/or user perceptions (via the survey) then they should make motivate these analyses earlier on by using relevant literature that frames why we should care about these results. As of now, these sections come out of the blue and it is unclear what contribution these analyses make to our understanding of political behavior or digital media. The section on misinformation is especially problematic, as it relies on cosine similarity matching of sentence embeddings which strays in a very significant way from how misinformation is detected in other studies. It is, of course, quite possible that this technique is used, but the authors need to convince the reader that this is a reasonable analysis. I know this is mentioned in the limitations section, but that is not enough to justify keeping that particular analysis in the paper as it is not even motivated in the frontend sections of the paper. I believe the authors should either keep these analyses but properly motivate them or leave them out entirely and focus instead on the experiment that is central to the paper.

8) An inherent limitation in the study design, which is mentioned in the limitations section, is that it captures a very limited time window for these sock puppet accounts. In particular, we see recommendations for an account that was only very shallowly trained with content, which of course does not capture what the algorithm is doing for the vast majority of actual accounts that are seeded with a lot more content than these sock puppet accounts. As such, what the study is picking up is ideological skew and negative partisanship in algorithmic recommendations that are made VERY EARLY ON in a user's time on the platform. It is not at all clear how much these results would generalize to how the algorithm works for users who have engaged with the platform for much longer. This seems like a pretty important limitation, yet the authors simply gloss it over in the limitations section. More discussion on the implications of this is warranted, and the authors should warn readers that we simply cannot use these findings to generalize to the typical TikTok user.

Minor comments:

1) Christian Bail is a trained sociologist, not a trained political scientist (his book is listed as a reference in political science instead of sociology). Also, Vosughi et al. are not trained in communication studies (with the possible exception of Deb Roy) – they are in computer science / information systems scholars.

2) For channels with fewer than 10 videos, the authors supplemented with up to 30 additional videos from TikAPI. How was the partisan alignment of these additional videos measured? Did this also go through LLMs as with the other videos collected?

3) It would be useful to see the top accounts that did not meet the 75% threshold to be assigned a partisanship label. I assume many of these are not political, but some may be political but less biased, and a breakdown based on whether or not these were deemed political by the LLMs would also be useful to see.

4) It is weird to see IQR, which I assume is short for Interquartile Range, depicted as a single number rather than a range. How was this calculated? Also, the authors should mention this in the main text if they are reporting it in the table. They might also add a note about how total (sum) likes across the top 20 accounts skewed towards Democrats, even though median likes skewed towards Republicans.

5) It is a bit unclear from the text what time represents. Each bot ran for a week, correct? So that the time effect implies that, as the election nears, there is a larger ideological skew in recommended videos for a new bot (rather than the alternative interpretation, which is that the skew for a given bot increased the longer they were on the platform). The authors might specify this alternative and highlight that this is *not* what's being measured, as this would seem to be important to study in future research (and, as far as I can tell, is the only way to address point 8 above - that the longevity of the bot accounts is tiny compared to the average user)

6) Figure 5 should be moved to later on in the manuscript, after it has been discussed.

(Remarks on code availability)
I took a very cursory look at the Github repository, selecting a few files and viewing them on Github for a minute or two each. I did not do a comprehensive review of all of the code in the repository, nor did I attempt to replicate the code in the repository.

Version 2:

Reviewer comments:

Referee #1

(Remarks to the Author)
I am fully satisfied with the authors' responses and corresponding changes in the manuscript. I recommend the publication of this piece.

(Remarks on code availability)

Referee #2

(Remarks to the Author)
We thank the authors for their response, which addressed many of our concerns. However, there are still a few outstanding issues that should be addressed before publishing. We therefore recommend another R&R.

1. Why do the authors think this is happening?

The authors added the following passage to the Robustness checks section:

"However, if there are more Republican users or content creators on TikTok, if Republican-aligned content is more engaging, or if Democrats are more interested in Republican-aligned content than Republicans are in Democratic-aligned videos, then this ideological skew is exactly what we might expect TikTok's algorithm to produce. Alternatively, if user behavior cannot explain the asymmetries we observe, it would suggest that there are internal governing rules governing the algorithm's behaviour that cause these results, independent of user behavior."

We agree that this statement is helpful. However, it comes deep in the paper (page 13). There are several passages earlier which seem to attribute the findings to recommendation algorithms without acknowledging the inferential challenge the authors face. Here are a few examples from the paper that nudge the reader toward the algorithm explanation without acknowledging the points made in their response:

From the abstract: "These asymmetries … persist … and are driven primarily by negative-partisanship content. Our findings suggest that recommendation algorithms can play an important role in shaping users' media diets."

From "Our Contributions": "Our findings reveal a significant Republican bias in TikTok's recommendation algorithm" sounds like a settled verdict on the algorithm itself.

The authors also suggest they "isolate the causal role of algorithms" in the introduction.

The authors are much more clear about the design of the paper and the assumptions they have to make in their memo:

"Our goal was to study partisan asymmetries in TikTok's recommendation algorithm, and indeed we found significant asymmetries in co-partisan and cross-partisan recommendation rates. There are only two plausible factors behind these asymmetries. Either user behavior has trained" the TikTok recommendation algorithm to produce partisan asymmetries, or else there are internal governing rules of the TikTok algorithm that have produce[d] this result independent of user behavior;

these governing rules may be intentionally partisan or they may not. Importantly, if we find that the observed asymmetries cannot be explained by the user behavior, then the rules governing the algorithm must be Responsible."

"In our study, we systematically rule out all plausible routes through which user behavior might result in the partisan asymmetry, including content type, channel- and video-level engagement metrics, and co-viewership networks, based on the available data and within the constraints of our experimental design. Our goal behind ruling out all those user-centered explanations was to strengthen our conclusion that the asymmetries could indeed be influenced, at least partially, by the algorithm's governing rules."

"Crucially, we are careful throughout the manuscript to avoid asserting that TikTok is intentionally promoting pro-Republican or anti-Democrat content. Nonetheless, our findings are of potential public concern, regardless of whether they stem from intentional algorithmic design choices made by TikTok. Indeed, an algorithm which asymmetrically recommends partisan content may have potential significant downstream implications on user behavior."

We recommend that these points be clearly stated in the manuscript itself. They should be integrated throughout the paper (framing, intro, abstract, conclusion, etc) and not just on page 13. We also recommend against using the term "bias" as in the quote from the "Our Contributions" section above - the authors use the term "skew" elsewhere, which is preferable. A more precise alternative might be "Republican-leaning skew in recommendations that cannot be explained by the user engagement metrics we observed" (perhaps with a reference to the Robustness section).

2. Alternative explanations for asymmetry

The authors use data to address concerns about possible asymmetries between the Democratic and Republican videos used to train Tik Tok algorithm. Their response cites insignificant differences in embedding space, comments on videos by party alignment, and rates of election-related videos and positive versus negative partisanship. These are all helpful in ruling out any obvious differences between the conditioning content within the limits of the available data. However, we still believe the authors should more explicitly warn readers that there may still be unobservable asymmetries in the conditioning stage. For example, unobserved account-level factors may be sorting users into groups that are not addressed in the new feed condition validation section. Because eight of the twelve Democratic seed channels are official office holders and the Republican set skews toward independent creators, the follower communities attached to those accounts may differ systematically in ways that the authors' comment-level analysis cannot detect. If TikTok clusters users partly on latent engagement profiles, the bots could inherit those cluster-level characteristics despite comparable video semantics. While an account-level analysis is understandably out of the reach of the study, we believe it's important to acknowledge this point and make these limits of the analysis transparent.

The authors might also consider whether the asymmetry of concern is not positive vs. negative partisanship or Democrat vs. Republican but the interaction between negative partisanship and Republican alignment. As Figure 7A shows, the rate of positive partisanship is lower among R videos and the rate of negative partisanship is higher. Neither is significant but the difference in differences is potentially of concern. For instance, Republicans are more likely to share content from low quality news sources both among the mass public https://www.nature.com/articles/s41586-024-07942-8 and elites https://academic.oup.com/pnasnexus/article/1/4/pgac186/6695314. Do the authors observe evidence that anti-Democratic videos perform better? It would be helpful to stratify their analyses by negativity and party rather than just party to see if the results are robust and/or to estimate models with party X negativity interactions.

7. Other points: Should the authors be using a false discovery rate correction?

The manuscript states that it applies Bonferroni adjustments (noted briefly in text and figure captions) but does not explain or justify that choice. Please briefly clarify the set of analyses to which you are applying Bonferroni adjustments and explain why they are preferred over other FWER or FDR methods (e.g., Benajmini–Hochberg). You may also wish to report both adjusted and unadjusted p-values so readers can gauge robustness.

(Remarks on code availability)
We confirmed revisions to the code

Referee #3

(Remarks to the Author)
I co-reviewed this manuscript with one of the reviewers who provided the listed reports.

(Remarks on code availability)
Yes, all of the code was functional, and the code for all figures was present.

Referee #5

(Remarks to the Author)
In their revised manuscript, "TikTok's recommendations skewed towards Republican content and negative-partisanship during the 2024 U.S. elections," the authors take several measures to address my comments on their previous draft. These

efforts are commendable, and I appreciate that the authors have taken the time to review my comments and respond accordingly. The addition of the section on “Conditioning Stage Validation” is a very welcome step towards additional transparency in the study’s experimental manipulations. The removal of the section on misinformation is justified, given both that it relied on unusual methods for identifying misinformation and that the section was not motivated and served as somewhat of a distraction from the study’s main findings.

Unfortunately, I still believe that the paper suffers from too many issues to recommend publication. The main issue is that the discussion and, to a lesser extent, the analysis are too unfocused for a Nature article. There is too much discussion outside of the study’s main findings and this makes it difficult to decipher the main contributions of the article (of which there are several, but they currently get lost). Despite my previous comments, the authors still choose to frame the abstract and introduction as a test of user behavior vs. algorithmic recommendations. As I mentioned previously, and as the authors agreed in their rebuttal, the very manipulation that the authors use, which relies on seeding accounts with partisan videos, is simulating user behavior. Furthermore, the authors simply do not need this positioning, and it serves to distract from the main findings rather than illuminate them. In my opinion, the authors need to do substantial filtering down of their text to focus on presenting their main findings in as clear a way as possible, and try not to pontificate on general topics in the broader intellectual space (e.g., social media “harms”) that they cannot speak to. This is a major weak point of the manuscript as it is currently written. Rather than simply adding text to address the reviewer comments, the authors should focus the text down substantially to improve readability.

In addition, the authors do a poor job of discussing the effects to which they can actually speak. One of the reasons is the insistence to continue using ‘negative partisanship’ as their jargon of choice without properly defining it or separating it from related (but distinct) concepts. For example, as far as I can tell, the authors (e.g., in the abstract) refer to Democrats seeing anti-Democrat content as ‘negative partisanship’ when this term would typically refer to Democrats seeing anti-Republican content. They also refer to ‘opposite-party recommendations’ which further confuses multiple distinct phenomena (e.g., Democrats seeing pro-Republican content and Democrats seeing anti-Democratic content). There is no need for all this confusion, and it arises because the authors discuss things using abstract terms instead of speaking to the different kinds of effects that they uncover. They can simply refer to Democrats seeing pro-Democrat content, anti-Democrat content, pro-Republican content, or anti-Republican content (and do that same thing for Republicans) instead of lumping these into terms like negative partisanship and opposite-party recommendations, but if they do choose to use these terms then they need to define them precisely and stick to those definitions.

Another concern arises from the new section that the authors added on validating their conditioning stage. While the authors argue that there is no skew in the videos they used for seeding, their own analysis shows the opposite and in the predicted direction – in particular, Figure 7A shows that Republican bots were more likely to be seeded with negative partisanship than positive partisanship, while the difference for Democratic bots was not statistically significant (or, at least, the confidence bands are overlapping). In general, this may be OK, but the authors need to acknowledge it and discuss it rather than pretend like these effects don’t exist. In particular, it might be a problem if the authors went out of their way to seed videos that differed across parties. However, if in the task of selecting videos the authors came across more negative partisanship videos targeting Republicans than Democrats, that could be an artifact of the supply of content that is available – in which case, it may actually match the true conditions that real users are in when using TikTok.

Of course, this does put wrinkle on the effects that the authors find, and especially the finding that the Republican bots were recommended more videos containing negative partisanship than the Democratic bots. Are these effects due to the seeding asymmetry or to an algorithmic asymmetry? It is possible that we cannot know using the specific study that the authors conducted. But if the authors were upfront about this and left room for discussing this issue, it might be something that does not altogether invalidate the paper. Such a discussion may also be the appropriate place for a mention of the debate around user behavior vs. algorithmic filtering, since it is directly relevant here in a way that it isn’t to the broader paper (which, again, I believe is about partisan asymmetries in algorithmic recommendations). Another interesting finding that the authors could highlight more is that the asymmetries appear to increase as the election nears (i.e., see Figure 2 A, B, and C). If the seeding videos were the same across the different bots that were deployed over time, then the partisan asymmetry in seeding could not explain this trend - which may be one way of addressing the seeding asymmetry.

Currently, there is a story to tell about partisan recommendations on TikTok that the authors tap into but the paper still falls way short of motivating and presenting in a clear and focused manner. There is simply too much discussion that is unrelated to the specific study that the authors present. Below, I include a few more specific comments to complement what I have already stated, and I would be happy to review a revised manuscript that takes these comments seriously and makes a significant effort to revise the paper in a way that makes for a clear and concise read.

1) The addition of ‘negative partisanship’ to the title makes it less accessible to a general audience as most readers will be unfamiliar with the term. I suggest reworking the title to highlight the main findings in a clear manner and without jargon. That is, the title could be about anti-Democrat messaging to Republicans or something similar. Moreover, the hyphen (-) in negative-partisanship is odd and should be removed. I also suggest saying ‘skew’ instead of ‘skewed’ to imply that, unless something recently changed, the algorithm likely still functions in the way that the authors found.

2) I find the sentence added to the abstract to be very confusing. To me, negative partisanship is NOT about Democrats seeing anti-Democrat messaging. Rather, negative partisanship means having Democrats see anti-Republican messages instead of pro-Democrat ones, or Republicans see anti-Democrat messages instead of pro-Republican ones. Judging from Figure 4 (A and B), the interesting effects (to me) include:

a. Republicans see more anti-Democrat messaging that Democrats see anti-Republican messaging (more negative partisanship for Republicans than Democrats)
b. Democrats see more anti-Democrat messaging than Republicans see anti-Republican messaging. I believe this is, perhaps, the effect mentioned in the abstract. I do not know what you call this – self-critical messaging, maybe? – but if the authors are going to focus on this effect then they should separate it conceptually from negative partisanship.

3) It is a bit weird to refer to 'the type of partisanship' videos reflect (e.g., page 3). In particular, videos don't reflect negative or positive partisanship, since the partisanship of the audience will determine in part whether they are being exposed to negative partisanship. For example, a Democrat seeing a video that is anti-Democrat is not typically considered to be negative partisanship. A Republican viewing the same video would constitute negative partisanship. The authors could avoid all of this by not relying so much on the jargon of 'negative partisanship' and instead discussing things in simpler terms – people viewing information that is critical of their opposite side vs. their own side, or information that is supportive of the opposite side vs. one's own side, instead of discussing 'types of partisanship' which makes little sense.

(Remarks on code availability)

Version 3:

Reviewer comments:

Referee #2

(Remarks to the Author)
We thank the authors for their response, which addressed many of our concerns. We recommend making a few minor changes in consultation with the editor before proceeding to publication.

1. Why do the authors think this is happening?

With the authors' edits to the abstract, introduction, and the contributions and political recommendation rates sections, our concerns that the paper too strongly implied that TikTok's algorithm itself was the cause of the partisan asymmetries—beyond the scope of the design—have largely been addressed. We also appreciate the clearer early definition of skew and the replacement of "bias" with "skew" throughout.

One of the new paragraphs added to the "Our Contributions" section states that the "plausible user-centered explanations" are "ruled out." This omits the necessary caveat (noted in our earlier review of the Sensitivity Analysis section) that the observed skew could still reflect unobserved internal engagement metrics (e.g., dwell time). A small edit to the new paragraph would resolve this concern (edits indicated with *):

Our findings reveal a significant Republican-leaning skew in TikTok's recommendations—one that persists across states even after accounting for channel- and video-level engagement metrics. By systematically ruling out plausible user-centered explanations ***to the extent possible with the available data***, we provide evidence that the asymmetries we observe may be influenced, at least partially, by the algorithm's governing rules, though not necessarily by intentional design choices.

Outside of this small concern, we are satisfied with the authors' edits.

2. Alternative explanations for asymmetry

We asked the authors to acknowledge that unobservable asymmetries may remain in the conditioning stage, specifically that Democratic seeds were disproportionately elected officials, while Republican seeds skewed toward independent creators. We suggested the authors warn that latent account-level clustering could influence results. The authors added cautionary notes to the Experimental Setup and Limitations sections explaining this possibility, which we believe clarify the limits of the inferences they can make and improve the transparency of the study's design. We are satisfied with these edits.

2a. Party x negativity interactions

We suggested that the authors estimate models with party x negativity interactions. The authors clarified that they had already run such interaction models, and they updated the manuscript to make this explicit. They also added engagement metrics broken out by the five categories (Pro-Dem, Anti-Dem, Pro-Rep, Anti-Rep, Neutral) in the supplementary materials. We are satisfied with these substantive changes.

We did notice a few very minor labeling inconsistencies in relevant figures that could be corrected for clarity:

Inconsistency in label wording:
-Figure 2H: "% of negative partisanship videos" vs. Figures 4E & 3C: "Proportion of negative partisanship videos."
-Inconsistency in label ordering under "Conditioning Metrics":
-"Number of conditioning videos watched" appears third in Figures 4E & 3C but second in Figure 2H.
-"Conditioning videos engagement score" appears second in Figure 4E but third in Figure 2H.

-Minor formatting: “Length” is capitalized in “Video Length” across Figures 4E, 3C, and 2H.

These are only aesthetic issues and do not affect the results, but fixing them would improve consistency and interpretation across figures.

3. Other points: Should the authors be using a false discovery rate correction?

We asked the authors to clarify where Bonferroni corrections were applied, why they were chosen over other adjustments, and to provide both adjusted and unadjusted p-values.

The authors have made progress: Supplementary Tables 15–17 now report unadjusted, Bonferroni, and Benjamini–Hochberg (BH) adjusted p-values, and figure captions for Figures 2H, 3C, and 4E note that multiple-comparison corrections were applied.

However, several points remain unclear:

-Justification: The manuscript does not explain why both Bonferroni (family-wise error rate) and BH (false discovery rate) corrections are used or why they are treated interchangeably. Family-wise error rate controls the probability of any false positive result across all tests, whereas false discovery rate controls the expected proportion of false positives among the rejected hypotheses. These are conceptually distinct approaches; using both without explanation is confusing.

-Which p-values define significance in figures: It appears Figures 2H, 3C, and 4E highlight coefficients as significant based on unadjusted p-values, even when the corresponding Bonferroni and BH values are not below 0.05. For example, in Supplementary Table 15, “Proportion of negative partisanship videos” and “Number of conditioning videos watched” are statistically significant when unadjusted but not after correction, yet Figure 2H presents them as significant.

-Inconsistencies between Bonferroni and BH: In one case (Video count under Recommendation channel metrics, Table 15), Bonferroni and BH differ in whether a result is considered significant, but the manuscript gives no guidance on how to interpret such disagreements.
While we agree with the authors that the multiple comparison results do not affect the paper’s substantive conclusions, the current presentation is confusing. If they proceed with using both adjustments, we recommend that the authors briefly explain in the main text or appendix (a) why they use both Bonferroni and BH corrections, (b) which set of p-values (unadjusted, Bonferroni, or BH) underlies the significance markers in Figures 2H, 3C, and 4E, and (c) how they handle disagreements between the two comparisons. It isn’t obvious to us why using both would be necessary, so we would advise using BH as it is more appropriate when the goal is to limit the expected proportion of false discoveries rather than the probability of any false discovery.

(Remarks on code availability)

Referee #3

(Remarks to the Author)
I co-reviewed this manuscript with one of the reviewers who provided the listed reports.

(Remarks on code availability)
From my review, all results are reproducible.

Referee #5

(Remarks to the Author)
In their second revision of "TikTok’s recommendations skewed toward oppositional and Republican-aligned content during the 2024 U.S. elections," the authors make some welcome edits to their previous manuscript. The improved focus of the manuscript is noticeable, and I appreciate the lengths to which the team went to improve the readability of the paper. I believe this round of edits was much more successful than the previous round, and I encourage the authors to continue focusing on the specific theories to which the authors can speak to and the actual contributions their study makes.

I still believe that there are several issues with the way that the study is motivated, the data are presented, and the amount of unnecessary language that remains in the manuscript for a journal such as Nature. Below, I highlight where I believe some further interventions are needed.

1) the abstract is quite wordy and yet still does not address the findings of the analysis of top accounts or the survey or topic modeling section. These additional analyses thus seem to come out of nowhere, are not motivated, and their findings are not properly placed within aims specified by the authors early on in the paper. I suggest either disposing of these findings or including them in the abstract as well as motivating them in the introductory portions of the paper. As of now, it still feels like these descriptive analyses are motivated by data availability and not theoretical or methodological contributions. The abstract also feels repetitive, with the main findings stated twice (once with percentages and once without) but with different wording. Such slopinness is unfortunate for a manuscript that is this late in the review process.

2) the early focus on affective polarization is odd, and especially in the subsection heading, since the article does not use any measures typical in this literature (thermometer ratings obtained through surveys). Incidentally, these could have been nice measures to add as the team did launch a survey, but no matter. The proper framing would seem to be prior work on algorithmic amplification rather than psychometric measures that the paper cannot speak to. A better section heading would be Social Media, Something, and Content Polarization where "Something" might be about Negativity, Political Orientation, Partisanship, Algorithmic Recommendations, or some other construct that is actually addressed in the analyses.

3) I appreciate that the authors note limitations early on in the manuscript, but I believe they should not preemptively do this but rather report the actual findings first without unnecessary pontificating. Then, they should leave discussion of limitations until after the design and results have been discussed so that these limitations can be properly assessed by readers of the paper.

4) I am confused about Figure 2. The gap between Dems and Reps clearly increases over time in the top panels, but the middle panels show a straight line. Shouldn't they be increasing?

5) Why use LPMs instead of logit or probit? If there were interactions in the model then perhaps an LPM would be reasonable, as interaction terms in nonlinear models are more difficult to interpret and depend on other covariates. However, in most of the models there are no interaction terms, as far as i can tell. More justification using the appropriate literature is needed here.

6) There appear to be too may robustness checks and sensitivity analyses in the main text for a Nature article. I might mention these briefly in the main body and move most of the text to the appendix.

7) Figure 4 says that we should expect data to be shown "over time" but none of the plots appear to have a time axis

8) what is "negative out-party content" (p. 21)? The authors have been using anti-Democratic/Republican, co/cross-partisan, etc. and should make sure to stick to the terms they have actually defined throughout

9) It is unclear how the comparison is made in the x-axis label of Figure 5. It would help if the authors included the actual metrics they use as axis labels. This also holds for "mismatch proportion" - the plots should be readable without reference to the manuscript text.

(Remarks on code availability)

Version 4:

Reviewer comments:

Referee #2

(Remarks to the Author)
We thank the authors for their response, which addresses all of our concerns. We are recommending the manuscript for publication.

1. Why do the authors think this is happening?

The authors incorporated our suggestion to add a caveat that the observed skew could still reflect unobserved internal engagement metrics (e.g., dwell time) in the relevant paragraph of the “Our Contributions” section. This revision addresses our final concern on this point.

2. Party x negativity interactions

The authors implemented our minor aesthetic suggestions for Figures 2H, 3C, and 4E. These panels are now consistent across figures, and we are satisfied with the revisions.

3. Other points: Should the authors be using a false discovery rate correction?

We asked the authors to clarify the justification for using both Bonferroni and Benjamini–Hochberg (BH) corrections and why they were treated interchangeably. We suggested explaining the rationale for multiple-comparison corrections and selecting one approach (preferably BH). The authors addressed this concern by relying exclusively on BH corrections, no longer reporting Bonferroni-adjusted results, and explaining their choice in the main text (p. 39):

“Given the large number of coefficients tested across related models, we adjust for multiple comparisons using Benjamini–Hochberg (BH) corrections, which control the false discovery rate.”

We also asked the authors to resolve inconsistencies in Figures 2H, 3C, and 4E regarding how BH- and Bonferroni-adjusted p-values were reflected in the significance indicators. The figures have been corrected, and the significance markers now

align with the BH-adjusted p-values.

Finally, we had noted that the manuscript did not articulate how cases would be handled in which Bonferroni and BH adjustments led to contradictory conclusions. This concern is now moot given the exclusive use of BH corrections.

(Remarks on code availability)

Referee #3

(Remarks to the Author)
I co-reviewed this manuscript with one of the reviewers who provided the listed reports.

(Remarks on code availability)
I've ran the code and verified all of the figures are replicable

Referee #6

(Remarks to the Author)
I thank the authors for the opportunity to review this work. As a new reviewer added to the process after two rounds of revisions, I am focusing my efforts on assessing the extent to which comments from the previous round of reviews were addressed. That said, I have read the manuscript and supplemental materials in full and have considered the standard set of criteria I would normally use for an initial review as well.

This paper uses and experimental approach to investigate whether or not partisan differences can be observed in TikTok’s recommendation system. The authors find Republican-seeded accounts receive more co-partisan recommendations while Democrat-seeded accounts receive more cross-partisan recommendations. They argue this suggests TikTok’s algorithm contributes to political polarization. This work is original and significant, it will be of value to researchers in a range of fields. Overall, I believe the authors did a good job of replying to past reviews and believe there are only a few minor issues to address now.

Request 1: A previous review commented on the abstract and requested substantial changes. I believe the edits to the abstract now ensure it is clear and concise and related edits in the introduction makes it clear why each of the different analyses are included. However, the opening sentence of the abstract suggests the study will examine self-selection versus algorithmic curation, yet this study actually only looks at algorithmic curation. As such, a very small re-frame of the first sentence is requested.

To the best of my ability I have assessed whether the various methodological requests from previous reviews were made. I see no red flags. That said, I do not have computer science expertise and cannot speak to the appropriateness of the statistical analyses chosen or performed for the experiment.

Request 2: That said, in my review of the methods, I did note a new item. It is highly irregular in my field to report survey results before describing the sample of respondents. On page 20 the 1008 respondents are described as “U.S.-based TikTok users” but nothing is said about whether the sample was random, representative, or what the demographic breakdown of the sample actually was. I see this as essential information for interpreting the results. I am not suggesting table 28 in the supplemental materials need to be move to the main body, but I do think a single sentence describing the sample and a reference to the table in the supplemental materials is appropriate here. That said, I will defer to the standard approach for the journal and rely on the editors to make the final call.
Previous reviews commented on small inconsistencies in a range of figures and graphs. These appear to be addressed.

Request 3: While reviewing these I did also note an accessibility concern. Many of the figures and graphs are unlikely to be accessible for people who are colourblind and for people who rely on grey-scale settings. I recommend all figures and graphs be examined to ensure they are accessible. Using different patterns or shapes in addition to the existing colour variation is likely the easiest way to accomplish this.

Next, I offer three optional suggestions related to the discussion of limitations:
Request 4: While the authors do appropriately point to the limits of using LLMs to analyze content and of relying on only transcripts and not visual cues, there is no mention of the limits of focusing on exclusively English content. A brief mention of this limitation reflecting would be useful given that the political information sharing on TikTok in non-English languages in the US might reasonably be different given that it likely comes from minority groups who may use and be impacted by the platform differently.

Request 5: 75% seems like a high threshold for amount of content that is partisan aligned – many creators add in content that is not political or is more neutral in order to be able to monetize, avoid de-prioritization, or to perform authenticity. Ideally, I’d like to see a sentence on page 9 in the top paragraph commenting on the high threshold for what counts as a partisan account or a line added to the limitations section.

Request 6: The bots were trained in a similar way in order to establish an ideological stance for each sock puppet, but this assumes that actual Democrats and Republicans would consume content in the same way. However, we know there are partisan differences in political information seeking practices and exposure via social media and it is therefore possible that training the sock puppets in the same way reduces external validity. Consider noting this as a limitation.

Request 7: Finally, the bibliography, while including an appropriate range of sources, contains many capitalization errors such as journal names and proper nouns. Beyond standard editing, please also note danah boyd is spelt all lower case.

(Remarks on code availability)

Open Access This Peer Review File is licensed under a Creative Commons Attribution 4.0 International License, which permits use, sharing, adaptation, distribution and reproduction in any medium or format, as long as you give appropriate credit to the original author(s) and the source, provide a link to the Creative Commons license, and indicate if changes were made.
In cases where reviewers are anonymous, credit should be given to 'Anonymous Referee' and the source.
The images or other third party material in this Peer Review File are included in the article's Creative Commons license, unless indicated otherwise in a credit line to the material. If material is not included in the article's Creative Commons license and your intended use is not permitted by statutory regulation or exceeds the permitted use, you will need to obtain permission directly from the copyright holder.
To view a copy of this license, visit https://creativecommons.org/licenses/by/4.0/

Response to the Editor:

Firstly, we would like to thank you for the opportunity to revise our manuscript. We are confident that the changes suggested by yourself and the reviewers have considerably improved our manuscript. We have carefully addressed each of the key revision points you have raised as follows:

1. Reframing around user behavior vs. platform algorithm (R2, R3, R5): We have clarified our theoretical stance throughout the manuscript, removing language suggesting a clean causal separation between user behavior and algorithmic curation. We now explicitly state that our design cannot fully disentangle these forces, but instead provides valuable evidence of partisan asymmetries that persist even when user behavioral inputs are carefully standardized across experimental conditions.

2. Testing alternative explanations for asymmetry: We have added a new section titled "Conditioning Stage Validation" that rules out differences in seeding video properties as an alternative explanation of asymmetry. This includes analyses of: (a) election relevance and positive/negative partisanship balance, (b) audience partisanship through comment analysis, (c) semantic similarity using high-dimensional embeddings of seed videos.

3. Reframing of theoretical discussions (R1): We have revised our framing from "polarization" to "content polarization" or "polarization in recommended content" throughout, distinguishing this from "affective polarization" in users' attitudes and behaviors. We now explicitly acknowledge that our study addresses media environment polarization rather than downstream attitude changes, and have updated the title of the corresponding section and its content, as well as the discussion accordingly.

4. Clarifications on methodological issues (R2, R3): We have implemented standard error clustering by bot and week in all statistical analyses, explicitly documented deviations from pre-registration with justifications, and added sensitivity analyses for transcript availability and bot data missingness as covariates in our regressions.

5. Interpretation of findings given seeding limitations (R5): As mentioned before, we include a new section demonstrating the equivalence of seeding conditions. We have also addressed R5's other concerns regarding expanding our discussion of our limitations, particularly emphasizing that our findings reflect early-stage user interactions rather than long-term platform behavior.

6. Maintaining robust conclusions: Throughout these revisions, we have preserved the core empirical findings while appropriately tempering claims about mechanisms; on the whole, we believe our conclusions are now stronger than before. We've also enhanced the discussion of negative partisanship findings, which one of the reviewers identified as a key contribution, including this emphasis in our revised title.

Throughout this memo, we include the reviewers' original comments in black, our responses in blue, and text that is newly added to our manuscript in green.

Referee #1 (Remarks to the Author):

Key results: This study is a unique piece of evidence excellently documenting the power of filtering algorithms on the platform TikTok when it comes to politics. The comprehensive steps authors took to ensure the validity of their results is impressive. The key outstanding features, in my view, are 1) the combination of observational components (the audits) combined with content analaytical techniques (to identify stances) and additional analyses to exclude engagement-based asymmetries as confounders. 2) The longitudinal nature of the study allows a dynamic observation of algorithmic curation and its reinforcement learning based on user preferences. 3) The application of the study's interest in an electoral context helps uncovering TikTok's potential power on election outcomes, at least shaping the agenda and tone of content individual citizens receive.

It is striking that Republican accounts receive 11.5% more congenial content than Democratic accounts, emphasizing an asymmetry in the ideology-driven algorithmic curation of content on TikTok. This result has strong implications for content regulation policies.
The quality of this work is uniquely high and I recommend the editor's team to consider this piece for publication, provided that authors are willing to address the points I am raising below which, I think, could help increasing the ultimate contribution of this work.

Validity: I do not identify any key flaws the impede publication.

Originality and significance: These results have high significance for several disciplines, inter alia, political science, communication studies, political psychology, sociology, ethics as well as legal sciences as these findings clarify an imbalance in the distribution of ideology-based content, having users not being aware of this, leading to a inhbition of users' informational self-determination. Would Democrats keep on using this platform after being educated that their content feed does not equate their Republication counterparts in terms of ideological constitution?

Response:
Thank you for your generous evaluation of our work and for your thoughtful and constructive comments. Below, we respond point-by-point to each of your comments. We hope our responses satisfy your concerns.

The multi-methodological approach of this piece and its rigor is a clear strength. Still, there are a series of observations that I would like to see addressed:
1) There are a lot of numbers thrown around in the experimental setup und methodological section (audits, runs, videos, etc). While their interconnection might be clear for authors, it would be helpful to have a figure that indicates how many weeks, how many audits, how many videos, how many experiment runs, … were conducted and whether and how these numbers are interrelated. While Figure 1 is helpful in disentangling the experimental design, another one (maybe in the supplementary material) could help to comprehend the massive data set you rely this study on.

Response:

We agree that an additional figure would help clarify the structure and scale of the data collected. We have now added a new supplementary figure (Supplementary Figure 2) which we attach below that visually summarizes the number of experimental runs, weeks, accounts, and videos at each stage of our study. We've also added Supplementary Table 24 which summarizes the number of videos watched in each week at each stage of the experiment.

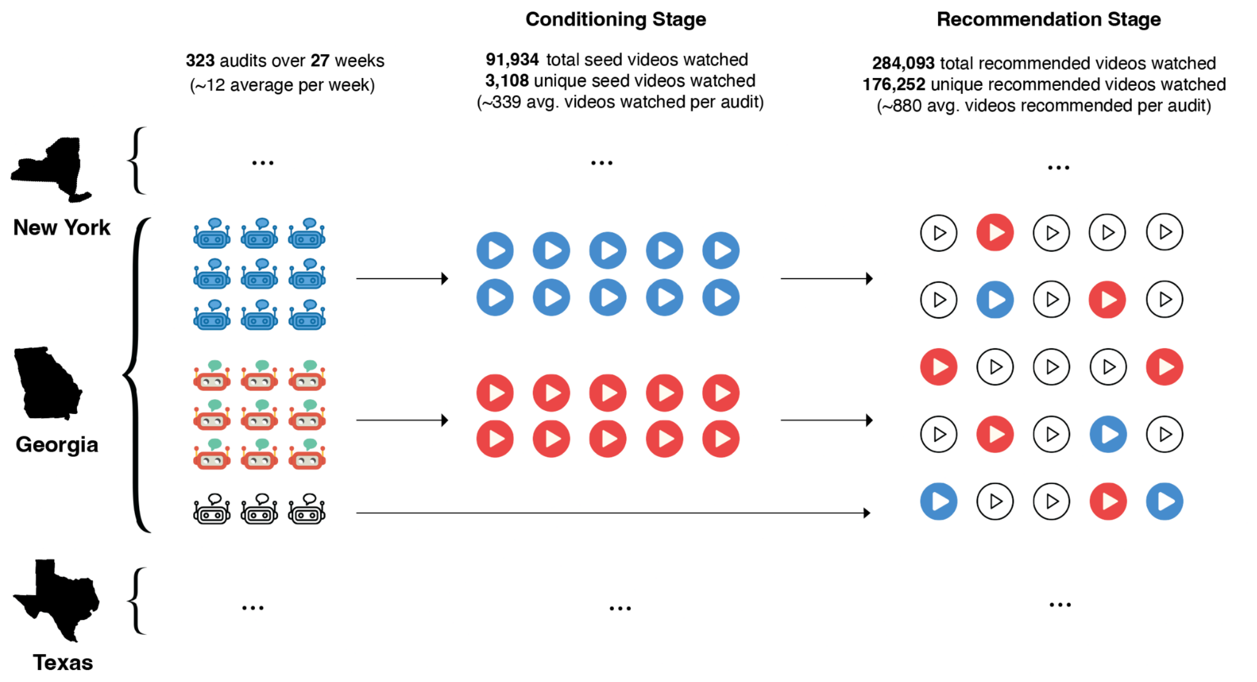

2) Authors justify their selection of States as assignment as likely Democrat, Republican or Swing State. Still, it is unclear why these three were chosen. Is there any rationale behind these three states? Do you expect that results are similar in other states that fall into the same category?

Response:

We selected New York, Texas, and Georgia to represent prototypical Democratic, Republican and Swing states, respectively, in the context of the 2024 U.S. election, based on the results of the 2020 U.S. election. This decision was ultimately also a logitistical constraint governed by the availability of VPN infrastructure. As such, we chose among the available VPN location options we maximally varied across the political landscape.

That said, we did not see large differences in partisan recommendation rates for bots of each partisan type across states. We now clarify our rationale in the Methods section by adding the following sentences:

“We chose New York, Texas, and Georgia because, on the basis of the 2020 presidential results, they serve as clear prototypes of (i) a reliably Democratic state, (ii) a reliably Republican state, and (iii) a competitive swing state in the 2024 electoral landscape. Practical constraints also shaped this choice, as only these three locations were simultaneously available and stable across our VPN infrastructure. Among the feasible options, these maximized geographic and partisan diversity.”

3) You write: „This process yielded 56 Democratic-aligned channels and 75 Republican-aligned channels, which we manually validated following best practices on channel-level classification tasks“ (p. 9) -> Is there a chance to compare this distribution with other numbers to see whether it resembles the actual prevalence of political channels on Tik Tok?

Response:

We share the desire for an external benchmark; unfortunately, no authoritative, platform-wide catalogue of political TikTok channels, by party or ideology, presently exists. TikTok does not publish aggregate statistics on the partisan affiliation of creators, and (to our knowledge) no third-party dataset provides a census-level view. Because of that gap, we consider this channel-classification procedure one of our study’s key contributions. That said, in a report published by Pew Research Center in 2024, they identify that TikTok stands out as the only platform among the five platforms they study (Facebook, Instagram, X, YouTube, and TikTok) where Democratic creators outnumbered Republican ones, with 25% and 28% of creators explicitly identifying as Republican or Democratic, respectively. The discrepancy between our results and those published in this report may be based in their inclusion criteria, where they necessitate an explicit declaration of support for a given political party, while our inclusion criteria is more flexible in this regard, relying only on the channel’s content as it relates to its partisanship. We’ve added the following to the Partisan Presence on TikTok section:

“As of the writing of this manuscript, no public, platform-wide census of partisan TikTok creators currently exists. Consequently, our validated list of 131 partisan channels constitutes a secondary contribution of this study. The only proximate reference point is a 2024 Pew Research Center report [1], which finds that just 25% of high-reach TikTok creators self-identify as Republican and 28% as Democratic. Pew’s lower partisan shares likely reflect its stricter inclusion rule, as they require an explicit self-declaration of party support, whereas our classification relies on the ideological orientation of the channel’s content, a broader criterion that captures politically active accounts even when creators do not state an affiliation.”

[1] - Galen Stocking, Luxuan Wang, Michael Lipka, Katerina Eva Matsa, Regina Widjaya, Emily Tomasik and Jacob Liedke. 8. News influencers on TikTok --- pewresearch.org. https://www.pewresearch.org/journalism/2024/11/18/news-influencers-on-tiktok/

4) What is the exact difference between symmetric and asymmetric polarization? In the explanation, they both read quite similar. It seems to me that in the asymmetric form the algorithm considers other users, too? Could you please explain?

Response:

We apologize that we were not clear in the original manuscript and appreciate this opportunity to clarify an important conceptual distinction. In response, we have revised the relevant section (Section: Political Recommendation Rates) to better differentiate the different polarization mechanisms. Specifically, symmetric polarization implies that both partisan groups are pushed further into their own echo chambers *at similar rates*, wheras asymmetric polarization implies that this intensification is uneven, i.e., one partisan group is exposed to more ideologically reinforcing content than the other. The relevant paragraph now reads as follows:

> *"A static recommendation system would simply mirror each user's existing partisan balance, showing Democrats and Republicans the same mixture they already consume, with no drift over time. A symmetrically polarizing system still personalizes, but does so evenhandedly: it amplifies whichever side of the spectrum a user prefers, and it amplifies both sides by roughly the same rate, so Democratic-leaning and Republican-leaning users become equally siloed.*
> *An asymmetrically polarizing system also amplifies like-minded content, but not evenly: one partisan group (e.g., Republican-oriented users) receives a noticeably stronger boost of co-partisan material than the other, deepening echo chambers for that side only. Finally, a partisan-biased system nudges everyone in the same direction, shifting recommended content toward a single party regardless of where each user started."*

Conclusions: Despite the excellence and high level of quality that is provided from a theoretical and methodological point of view, the conclusions drawn need to be refined:

5) You state: „we contribute to ongoing debates about whether algorithmic bias or user behavior is more responsible for the polarization observed on social media platforms" (p. 3) But you are not studying „polarization" per se. Your study presents the distribution of political content in the network of TikTok. This evidence does not allow any conclusions about polarization and the ultimate link between social media use and polarization is yet to be understood. The state-of-the-art is mixed and I would not take it as a given that social media (and human- or algorithm-based decisions) are responsible for a polarized system.

Response:

We appreciate your careful reading and agree that our original wording risked conflating two distinct concepts:

1. Polarization in users' attitudes and behaviors (e.g., affective or ideological polarization measured through surveys or election data), and

2. Polarization in the media environment—that is, the extent to which users are exposed to ideologically homogeneous or heterogeneous content.

Our study speaks only to the second phenomenon. We audit TikTok's recommendation system and document asymmetric partisan exposure in early-stage content feeds; we do not measure downstream changes in beliefs, emotions, or political actions. To avoid over-interpretation, we have revised the manuscript as follows:

We now replace the phrase *"is more responsible for the polarization observed on social media platforms"* to *"is more responsible for the polarization in recommended content observed on social media platforms".*

We also add the following:

> *"Here, we make the distinction between two distinct concepts. The first is ``affective polarization'', or the polarization in users' attitudes and behavior. The second is ``content polarization'', which is the extent to which users are exposed to ideologically homogeneous or heterogeneous content."*

In addition, where we contextualize our findings in broader debates, we explicitly note that content-level polarization is not a sufficient condition for attitude-level polarization and that prior work linking the two remains mixed. We also changed the title of the Section from "Social Media and Political Polarization" to "Social Media, Political Content, and Affective Polarization". Throughout this section, we are careful to use the phrase "content polarization" or "polarization in recommended content" when referring to either our own experiment, or to other literature which studies the aforementioned second phenomenon. Similarly, we use the phrase "attitude polarization" or "affective and issue polarization" when referring to work which addresses the first phenomenon.

Furthermore, in the Discussion section, we add:

> *"Compared to legacy social media platforms like Facebook and Twitter, TikTok's recommendation is more central to users' experience due to how users engage with the platform, which is almost entirely dominated by recommendations. However, as our design cannot adjudicate whether the asymmetries we observe translate into shifts in users' beliefs or behaviors, future work incorporating complementary research designs (e.g., longitudinal panel surveys, field experiments) that combine exposure data with attitudinal measures as necessary towards understanding the impact of content polarization on belief polarization."*

6) In the presentation of the survey results, you state the Republicans „were more likely to characterize the political content on their feed as positive, optimistic, agreeable, and pro-Trump“(p. 21). The fact that Republicans perceive to see more pro-Trump content the Democrats is not surprising. The key question is whether they perceived to access more pro-Trump content than Democrats perceived to access pro-Harris content? This would just be an interesting addition, since we all know that these perceptions may not fully reflect reality. Still, it would be interesting to know whether the imbalance is also resembled in users‘ perception.

Response:

We are gratified that you think this is an interesting analysis! We did indeed collect and analyze such comparative perceptions. This is what is described in the fifth row of Figure 5, where we find that Republican respondents saw more co-partisan content (i.e content supporting the Republican party) relative to Democratic users (i.e. content supporting the Democratic party). We agree with you that this was not clear enough in the text analyzing this result. We have adjusted the text and believe it is much clearer now. It reads as follows:

> *"Republicans were significantly more likely to report seeing an increase in Republican-aligned content (co-partisan content) than Democrats were to report seeing Democratic-aligned content."*

7) You write „these findings are consistent with growing evidence that conservative users in particular may be more likely to occupy ideologically homogeneous media environments“ (p. 24). This interpretation could be clarified. I am not sure whether it is a deliberate occupation by Republicans or whether it is just that the algorithms play in their hands?

Response:

This is an important point and we thank you for encouraging us to clarify. We now expand our discussion to explicitly consider both interpretations: (1) that conservative users may gravitate toward more ideologically homogenous spaces, which causes the algorithm to in-turn, serve such users more co-partisan content, and (2) that recommendation algorithms may amplify this tendency, causing conservative users to end up in more homogenous spaces. We note that our experimental design cannot disentangle these mechanisms fully, but we conduct extensive analysis ruling out theoretically important components of user behavior, and conclude that a significant component of our findings are driven by the algorithms themselves:

> *"These findings are consistent with prior research suggesting that conservative users may be more likely to consume more partisan-reinforcing content online [1, 2]. However, this pattern can be interpreted in two ways. On the one hand, it may reflect user-driven behavior, in which conservative users gravitate toward more ideologically aligned content, a pattern which recommendation algorithms adapt to. Alternatively, this may be directly due to algorithmic amplification, where the platform's recommendation systems disproportionately reinforce co-partisan*

exposure for right-leaning users. Our experimental design does not allow us to fully disentangle these mechanisms. Nonetheless, because all accounts in our study followed comparable conditioning protocols, the observed asymmetry suggests an algorithmic contribution that cannot be solely attributed to user choice. See our additional analyses in Figures 2G, 2H, 3C, 4C, 4D and 4E ruling out various components of user behavior as explanations for this observed asymmetry."

[1] - González-Bailón, Sandra, et al. "Asymmetric ideological segregation in exposure to political news on Facebook." Science 381.6656 (2023): 392-398.
[2] - Huszár, Ferenc, et al. "Algorithmic amplification of politics on Twitter." Proceedings of the national academy of sciences 119.1 (2022): e2025334119.

8) The interpretation of the results could be expanded as these results have a high significance from different perspectives. Maybe, the following questions could help extending the discussion and making the contribution of this piece clearer
a) Do these results clearly point to an ideological bias in the algorithmic filtering?
b) If yes, why so? Who is or should be responsible for this?
c) How are these results comparable with previous works on the ideological bias of algorithmic curation on social media?
d) Is TikTok unique in this regard? Is it because the algorithm is so powerful compared to other platforms?
e) What could effects of this imbalance in the distribution of political content be? How may it shape public opinion formation? How may it shape voting decisions? Is there any group that may be very susceptible in this context?
f) Does this observation indicate a violation of human rights? How would the EU Digital Services Act interpret these results?Is is transferrable to the U.S. system?

Response:

Thank you for these suggestions! We try and answer each of them below and include the changes in the manuscript which reflect these answers:

a, b: Our results do suggest an ideological bias in partisan recommendations, as evident by the difference in recommendation rates of co-partisan and cross-partisan videos seen by the Democratic and Republican-seeded accounts. While we cannot pinpoint the exact cause of this asymmetry (e.g., an intentional promotion of Republican content by TikTok), we do attempt to eliminate as many explanations as possible through our robustness checks (see the Robustness Checks subsection which we include below).

"Figure 2 establishes that TikTok shows more Republican-aligned content than Democratic-aligned content, and that Democrats receive more Republican-aligned content than Republicans receive Democratic-aligned

content. However, if there are more Republican users or content creators on TikTok, if Republican-aligned content is more engaging, or if Democrats are more interested in Republican-aligned content than Republicans are in Democratic-aligned videos, then this ideological skew is exactly what we might expect TikTok's algorithm to produce. In this section we show that these mechanisms cannot fully explain the observed difference in ideological content. First, we use a weighted random sampling approach to show that observed engagement metrics like channel size, video likes, and commenting, or linear combinations of the above, would not produce the observed ideological skew. Second, we show that the number of Democratic-aligned comments on a Republican-aligned video does not predict whether that video is shown more to Democrats. Neither of these two explanations, however, preclude the possibility that TikTok has developed a latent engagement metric that \textit{would} explain the observed ideological gap; our third robustness test develops a sensitivity analysis to show that for any hypothetical hidden engagement metric, the difference in that metric between Republican and Democratic videos would have to be 98 times bigger than the gap in likes to explain the ideological content gap we observe on TikTok in our experiments.

Sampling by Engagement Metrics

To verify that our results are not due to differences in the engagement metrics of Republican and Democratic videos or channels, we consider counterfactual scenarios where video recommendations are functions of these engagement metrics. We showed above that the TikTok algorithm recommends more Republican-aligned content than Democratic-aligned content, but this may not be surprising if there is more Republican content overall on TikTok, or if that content is more popular. In other words, Figure 2 assumes a counterfactual where Republican-aligned and Democratic-aligned videos appear in equal proportion; we consider alternative counterfactuals below. Our first robustness test answers the question: how big of an ideological skew in content should we expect under different counterfactual scenarios, and how does the observed skew compare?

To confirm that the skew towards Republican-aligned content exists even after accounting for potential differences in video engagement metrics that might produce baseline differences, we take a weighted-random sample of N videos (N being the number of videos watched by pairs of bots in a given week and experimental condition) with weights proportional to that video's \textit{engagement}, and calculate the proportion of Republican- and Democratic-aligned videos in that sample. We then compare these proportions to the observed proportion of recommended Republican and Democratic-aligned videos, and show that our bots received more such Republican-aligned videos than we would expect if recommendations were only a function of video engagement. To this end, we consider 48 scenarios where recommendation rates are a function of TikTok engagement metrics: video-level metrics such as a

video's likes, likes per recommendation, comments, comments per recommendation, plays, and share count, as well as channel-level metrics such as verification status, follower count, and cumulative like and video counts. We then aggregate the above metrics using principal component analysis (PCA) and extract the video loadings to produce a combined video engagement score, a combined channel engagement score, and a combined overall engagement score. For each of the 16 models described (nine video-level models, four channel-level models, and three combined models), we also compute two alternative versions, penalizing old videos linearly and exponentially, to up-weight more recent content.

We show the results from this counterfactual analysis in Figure 2G. The leftmost point represents the observed ideological skew (the proportion of Republican-aligned videos minus that of Democratic-aligned ones recommended to the bots). Each of the remaining points presents the expected ideological skew as computed by the 48 counterfactuals, shown along the x-axis; plot point markers represent the alternative methods for up-weighting more recent content. If the observed engagement metrics fully explain the partisan gap, we would expect the simulated gaps when we sample proportionate to those metrics to be close to the observed gap of approximately 0.2. We do not observe that. Instead, across all 48 counterfactuals, the observed bias toward Republican-aligned content substantially exceeded the counterfactual proportion based on engagement, with the majority of engagement metrics yielding a Democratic bias rather than a Republican one. Supplementary Table 7 lists the expected ideological skews as computed by each model, as well as independent t-tests comparing these values to that observed by the bots; see the Video Recommendation Counterfactual Models section in Methods for further details.

Asymmetric Homophily

In addition to these public engagement metrics, we also consider bias which may stem from co-viewership networks. Republican-aligned videos may garner more engagement from Democrats than vice-versa, which would explain why Republican-aligned videos are recommended to Democrats more frequently; we refer to this possibility as asymmetric homophily. A key implication of asymmetric homophily is that among Republican-aligned videos, those with more Democratic-aligned comments will be shown to Democrats more than those with fewer Democratic-aligned comments. We show that is not the case, suggesting that asymmetric homophily cannot explain the ideological skew we observe.

To test this, we collect a random sample of 500 comments from all political videos examined in our study and classify the content of each comment in the context of that video. From this we compute the proportion of comments ideologically aligned with the video (see the Comment Classification section in Methods).

We find meaningful differences between Republican-aligned and Democratic-aligned videos in the composition of comments, but not in the expected direction. We find that Democratic-aligned videos have a higher proportion of Republican-aligned comments than vice versa (12.9% versus 8.5%, $\chi^2 = 2.32$, $p = 0.13$), as well as a higher proportion of copartisan comments than Republican-aligned videos (63.1% versus 51.6%, $\chi^2 = 1.97$, $p = 0.16$). However, Republican-aligned videos have a significantly higher proportion of ``neutral'' comments that are not explicitly partisan (44.6% versus 26.3%, $\chi^2 = 7.53$, $p = 0.006$). We also include this metric in our weighted sampling robustness check (Figure 2G), finding similar results to our other observed engagement metrics. These results suggest that, at least with regards to commenting behavior, Democrats are not engaging with Republican-aligned videos more than Republicans are engaging with Democratic-aligned videos: we find no asymmetric homophily that would explain the ideological skew.

We formalize these analyses in a linear probability model (LPM) framework (Figure 2H). We conduct a regression at the bot-video recommendation level where the outcome variable indicates whether a video is an ideological mismatch as a function of that bot's conditioned partisanship, controlling for that bot's state, the week in which the experiment was conducted, the number of videos watched by the bot during its experimental run, the proportion of videos seen which included a transcript, as well as the engagement metrics of the videos and channels watched during the recommendation stage, in addition to those watched in the conditioning stage. We also implement two-way clustering by bot and week as recommendations may be correlated within bot and within a given time period. Lastly, we isolate the independent variables with a Variance Inflation Factor (VIF) value less than five (Supplementary Table 13 lists the VIF scores for each of the independent variables). Considering that all conditioning video- and channel-level engagement metrics had a VIF score greater than five, we compute a combined engagement score for those videos using PCA. In Figure 2H, the top-most point shows the LPM estimate on bot conditioning party. While opposition-comment proportion meaningfully predicts a video being mismatched, Democrat-aligned bots are 30.8 percentage points more likely to get a mismatch than Republican-aligned bots, on average, controlling for other variables and fixed effects. We show the full regression table for this model in Supplementary Table 15. Additionally, Supplementary Tables 18-21 include descriptive statistics of the engagement metrics of videos and channels seen by bots during the conditioning and recommendation stages.

Sensitivity Analysis

While we have shown evidence that observable engagement metrics and viewership patterns cannot explain the ideological skew in Republican-aligned

content on TikTok, we cannot rule out the possibility that there are unobserved or latent engagement metrics or viewership patterns, known to the TikTok algorithm but unknown to us, that would explain the gap we observe. For instance, a key engagement metric such as ``dwell time'', or how long a user spends watching a particular video, is not a publicly available metric published by TikTok. As such, we conduct a sensitivity analysis to calculate how strong such an unobserved factor would have to be to fully explain TikTok's apparent ideological skew.

Across our 14 observed metrics, there are four where Republican-aligned videos outperform Democratic-aligned videos: comments, likes, plays, and shares. If we consider only the optimal linear combination of those four in our weighted sampling approach, they explain 5.7% of the observed ideological gap. Therefore, for any latent or unobserved metric to fully explain that gap, Republican-aligned videos would need to outperform Democratic-aligned videos by 9.4 times as strongly as they do on the aggregation of comments, likes, plays, and shares (see Table 11 in Methods for the required scaling needed on the various engagement metrics analyzed). We argue that such a metric is unlikely to exist. We formalize the assumptions behind this in the Sensitivity Analysis component of the Methods section, in which we experiment with sampling from a variety of distributions."

c, d:

We have extensively reviewed the existing literature on algorithmic bias across social media platforms to position our TikTok findings appropriately. Previous research has documented asymmetric ideological exposure and engagement patterns on Facebook, Twitter, and YouTube, often shaped by user behavior and platform affordances. Notably, several studies suggest that user choice, rather than algorithmic curation alone, plays a more substantial role in shaping political exposure.

TikTok is indeed unique in this regard, and our study design specifically leverages this uniqueness. Unlike other social media platforms where users largely retain control over their content feed, TikTok algorithmically curates content with minimal input from users. This architectural difference allows us to isolate algorithmic effects from user choice in ways that previous studies could not. We have included this comparative discussion to the paper:

"These findings contribute to a complex and evolving understanding of ideological bias on social media, building upon previous research that has revealed varied patterns of political exposure and algorithmic behavior across different platforms. Gonzalez-Bailon et al. show that conservative users on Facebook are more likely to engage with misinformation and operate within insular networks [1]. Similarly on Twitter, Barberá et al. found that liberals were

more likely than conservatives to engage in cross-ideological retweeting on political topics [2], while Huszár et.al reported that the platform disproportionately amplified right-leaning content more than left-leaning counterpart. For YouTube, several studies found evidence of ideological congeniality and user migration toward extreme content [3,4], or left-leaning algorithmic bias [5]. At the same time, some experimental work has challenged claims of algorithmic radicalization, suggesting that self-selection remains a more powerful driver than algorithmic nudges on Facebook [6,7,8], Instagram [6], and YouTube [9]. Allcott et al. reinforced this perspective by showing limited causal effects of Facebook and Instagram use on polarization or electoral behavior [10]. We reconcile these mixed findings by studying a platform that sharply limits self-selection; TikTok's recommendation feed removes most avenues for users to curate their own content, thereby isolating the role of algorithmic architecture in shaping exposure."

[1] - González-Bailón, Sandra, et al. "Asymmetric ideological segregation in exposure to political news on Facebook." Science 381.6656 (2023): 392-398.
[2] - Barberá, Pablo, et al. "Tweeting from left to right: Is online political communication more than an echo chamber?." Psychological science 26.10 (2015): 1531-1542.
[3] - Haroon, Muhammad, et al. "Auditing YouTube's recommendation system for ideologically congenial, extreme, and problematic recommendations." Proceedings of the national academy of sciences 120.50 (2023): e2213020120.
[4] - Ribeiro, Manoel Horta, et al. "Auditing radicalization pathways on YouTube." Proceedings of the 2020 conference on fairness, accountability, and transparency. 2020.
[5] - Ibrahim, Hazem, et al. "YouTube's recommendation algorithm is left-leaning in the United States." PNAS nexus 2.8 (2023): pgad264.
[6] - Guess, Andrew M., et al. "How do social media feed algorithms affect attitudes and behavior in an election campaign?." Science 381.6656 (2023): 398-404.
[7] - Bakshy, Eytan, Solomon Messing, and Lada A. Adamic. "Exposure to ideologically diverse news and opinion on Facebook." Science 348.6239 (2015): 1130-1132.
[8] - Nyhan, Brendan, et al. "Like-minded sources on Facebook are prevalent but not polarizing." Nature 620.7972 (2023): 137-144.
[9] - Hosseinmardi, Homa, et al. "Causally estimating the effect of YouTube's recommender system using counterfactual bots." Proceedings of the national academy of sciences 121.8 (2024): e2313377121.
[10] - Allcott, Hunt, et al. "The effects of Facebook and Instagram on the 2020 election: A deactivation experiment." Proceedings of the National Academy of Sciences 121.21 (2024): e2321584121.

e: Our experimental design does not allow us to answer these questions directly, but we now attempt to better contextualize our findings in the Discussion section within the broader literature on social media polarization and political behavior. Indeed, the effect of partisan content consumption on voting outcomes is mixed, with Allcott et al. [1] showing limited effects within participants who deactivated their social media accounts prior to an election cycle. We also point to results from [2] which suggest that altering the recommendation algorithm of a YouTube-like platform had no detectable polarizing effects on users' attitudes in the short term, where they discuss the inherent limitation of field. Specifically, we include the following text:

> *"Liu et al. [2] further emphasize the difficulty of detecting attitudinal polarization effects, even in large-scale randomized controlled trials with users interacting with a YouTube-like recommendation system. Their findings suggest that while seed effects (i.e., the influence of initial content) can shape short-term attitudes under forced exposure, the broader influence of recommendation systems is modest when users exercise naturalistic choice."*
>
> [1] - Allcott, Hunt, et al. "The effects of Facebook and Instagram on the 2020 election: A deactivation experiment." Proceedings of the National Academy of Sciences 121.21 (2024): e2321584121.
>
> [2] - N. Liu, et al. "Short-term exposure to filter-bubble recommendation systems has limited polarization effects: Naturalistic experiments on YouTube", Proceedings of the National Academy of Sciences. 122 (8) e2318127122.

f: We again hesitate to speculate on this as this is not our domain of expertise, but we now point to work by Fukuyama and Grotto in the Discussion section which better aligns with your question.

> *"Our findings of TikTok's asymmetric content distribution raise potential concerns under the EU Digital Services Act, which requires large platforms to assess and mitigate systemic risks to fundamental rights and electoral processes. In contrast to EU's interventionist approach, regulatory responses would likely differ in the United States, where First Amendment protections have traditionally granted platforms broader editorial discretion [1]. The transferability of regulatory solutions across these systems remains complicated by such foundational differences, though historical broadcast regulation precedents suggest potential pathways for addressing algorithmic bias when platforms achieve monopolistic scale, despite considerable implementation obstacles in highly polarized political contexts."*
>
> *[1] - Fukuyama, Francis, and Andrew Grotto. "Comparative media regulation in the United States and Europe." Social media and democracy: The state of the field and prospects for reform (2020): 199-219.*

I really enjoyed reading this research and wish authors the best of luck.

Response:
Thank you once again for your review. We hope that we have adequately addressed all of your questions and concerns.

Referee #2 and Referee #3 (Remarks to the Author):

The submitted manuscript seeks to measure partisan bias in TikTok's recommendation of political content to Democratic- and Republican-leaning accounts using "sock puppet" accounts that are created and seeded by viewing aligned political content. The results are interesting and novel -- as the authors note, TikTok is less well understood than other prominent social media platforms and the data collection approach here is ambitious and impressive. However, their design cannot rule out alternative explanations for the asymmetries in recommendation patterns by partisanship that they observe. We therefore regretfully recommend that the article be declined. Our comments follow:

Response:

Thank you for your careful reading of our paper and your constructive review. We believe that your comments have helped us improve our manuscript considerably, and hope that our responses to your points below successfully address your concerns.

1. Why do the authors think this is happening?
The manuscript offers no convincing explanation for the observed partisan asymmetries in content recommendations. Is TikTok intentionally promoting more pro-Republican and anti-Democrat content or is it somehow responding to the preferences and behavior of users on the platform? The authors consider several possibilities but find the evidence is not consistent with each of them. This creates a gaping theoretical/interpretive hole.

Response:

We appreciate this important comment. Our goal was to study partisan asymmetries in TikTok's recommendation algorithm, and indeed we found significant asymmetries in co-partisan and cross-partisan recommendation rates. There are only two plausible factors behind these asymmetries. Either **user behavior** has "trained" the TikTok recommendation algorithm to produce partisan asymmetries, or else there are **internal governing rules** of the TikTok algorithm that have produce this result independent of user behavior; these governing rules may be intentionally partisan or they may not. Importantly, if we find that the observed asymmetries cannot be explained by the user behavior, then the rules governing the algorithm must be responsible.

In our study, we systematically rule out all plausible routes through which user behavior might result in the partisan asymmetry, including content type, channel- and video-level engagement metrics, and co-viewership networks, based on the available data and within the constraints of our experimental design. Our goal behind ruling out all those user-centered explanations was to strengthen our conclusion that the asymmetries could indeed be influenced, at least partially, by the algorithm's governing rules.

Crucially, we are careful throughout the manuscript to avoid asserting that TikTok is intentionally promoting pro-Republican or anti-Democrat content. Nonetheless, our findings are of potential

public concern, regardless of whether they stem from intentional algorithmic design choices made by TikTok. Indeed, an algorithm which asymmetrically recommends partisan content may have potential significant downstream implications on user behavior.

That said, we do agree that our manuscript could benefit from clarifying the motivation behind ruling out as many user-centric explanations as possible, and have revised the manuscript to better articulate this distinction. Specifically, we add the following to the beginning of the Robustness checks section:

> "However, if there are more Republican users or content creators on TikTok, if Republican-aligned content is more engaging, or if Democrats are more *interested in* Republican-aligned content than Republicans are in Democratic-aligned videos, then this ideological skew is exactly what we might expect TikTok's algorithm to produce. Alternatively, if user behavior cannot explain the asymmetries we observe, it would suggest that there are internal governing rules governing the algorithm's behaviour that cause these results, independent of user behavior."

2. Alternative explanations for asymmetry

Most importantly, we are concerned that the central finding of the paper - that Republican-seeded accounts on TikTok receive more congenial recommendations than do Democrat-seeded accounts - could be an artifact of the research design or other features of the platform rather than a true partisan asymmetry in algorithmic recommendation behavior.

Most notably, eight of the twelve Democratic-leaning accounts used for seeding the Democratic sock puppet accounts are official Democratic Party sources or Democratic members of Congress (e.g., Ro Khanna, Bernie Sanders, and Alexandria Ocasio-Cortez), whereas all twelve of the Republican-leaning accounts used for seeding the Republican sock puppet accounts are right-wing content creators who are not formally affiliated with the Republican Party (Tucker Carlson, Ben Shapiro, and various lesser-known accounts). The differences in the videos watched by the Democratic- and Republican-seeded accounts could easily reflect asymmetries between these two sets of videos that are not a reflection of partisanship. For instance, the observed differences in recommendation patterns could reflect how the algorithm responds to differences in ideological extremism or preferences for institutional versus independent sources rather than being an asymmetry in how it recommends content to Democratic- and Republican-leaning users.

Consider, for instance, how TikTok describes its algorithm as reported by Axios (https://www.axios.com/2020/09/10/inside-tiktoks-killer-algorithm):

"The algorithm identifies similar videos to those that have engaged a user based on video information, which could include details like captions, hashtags or sounds. Recommendations

also take into account user device and account settings, which include data like language preference, country setting, and device type."

"Once TikTok collects enough data about the user, the app is able to map a user's preferences in relation to similar users and group them into 'clusters.' Simultaneously, it also groups videos into 'clusters' based on similar themes, like 'basketball' or 'bunnies.'"

"Using machine learning, the algorithm serves videos to users based on their proximity to other clusters of users and content that they like."

There's no reason to necessarily think that TikTok is grouping users into "Democrat" and "Republican" clusters and making recommendations to them asymmetrically. One could easily imagine instead the accounts being assigned who watch videos from members of Congress being grouped into a cluster that is broadly interested in politics and receives recommendations that match that preference, while those who watch videos from conservative content creators might be clustered with people who have different viewing habits.

In general, the paper lacks theoretical depth on different types of algorithmic biases or recommendation patterns and how they might help explain the observed results. It would also be helpful to consider whether and how bias is appropriately defined in this context.

Response:

We thank you for raising this critical point about potential differences in seeding content outside of partisanship which may have downstream effects in recommendations. We agree that ruling out systematic differences in the nature of seeded videos is crucial to preserving the internal validity of our experimental design. In response, we have now added a new section in the manuscript titled "Conditioning Stage Validation" to the Methods, which presents a comprehensive set of analyses to assess whether Democratic- and Republican-aligned bots were exposed to semantically and ideologically comparable content during the seeding phase.

First, we compare the proportion of videos directly related to the U.S. election or major political figures and find no significant difference across partisan groups. Second, we analyze the prevalence of positive versus negative partisanship framing in the seed content and again find no significant differences. Third, we use comment-level analysis to evaluate the partisan leanings of video audiences, which were balanced across bot conditions. Finally, we embed the video transcripts in a high-dimensional semantic space and show that the centroids of the Democratic and Republican seed content clusters are nearly equidistant from neutral content, both in terms of their projected location and Euclidean distance, suggesting semantic parity in initial exposure.

Taken together, these checks provide convergent evidence that the seed videos viewed by bots across partisan alignments were comparable in their issue relevance, affective tone, audience reception, and semantic content. This additional validation strengthens our confidence that

downstream asymmetries observed in the recommendation stage are unlikely to be driven by skewed initial inputs. We copy the new section below:

"We take several steps to validate that the seeding delivered to bots of opposite party alignments was equivalent. A summary of this analysis is presented in Figure 7. First, we evaluate the proportion of videos directly related to the U.S. election or major political figures (as determined by the second question of the transcript classification pipeline described above). As shown in Figure 7A, both Democratic-aligned and Republican-aligned bots were exposed to a statistically similar proportion of election-related videos (t = -0.478, p = 1.0). Regarding positive and negative partisanship, we also find no statistically significant differences in the proportion of such videos between the two bot groups (Positive-partisanship: t = 2.19, p = 0.129; Negative-partisanship: t = -1.7, p = 0.373; Neutral: t = 1.63, p = 0.426).

Second, we classify a random sample of up to 500 comments posted under each of the partisan videos seen during the conditioning stage, using the comment classification procedure described in the previous section. As shown in Figure 7B, videos shown to Democratic- and Republican-aligned bots received similar rates of both party-aligned and opposite-party-aligned comments (Party-aligned comments: 60.2% vs. 59.9%, χ^2 = 0.008, p = 0.925; Opposite-party-aligned comments: 8.9% vs. 6.8%, χ^2 = 0.18, p = 0.67).

Third, we compute the embedding of the transcripts of all videos within our dataset using the embedding model proposed by Wang et. al [1], chosen as an open-source embedding model with performance on par with state-of-the-art models. We then compute the centroids of the sets of videos seen by Democratic-aligned and Republican-aligned bots during the conditioning-stage of their respective audits. The projection of these cluster centroids into two dimensions using PCA is illustrated in Figure 7C. To better understand the extent to which the sets of videos diverge semantically across partisan conditions, we compute the axis of maximal variation between Democratic and Republican videos by taking the unit vector connecting their respective centroids in the original embedding space. We then project the corresponding centroids onto this axis, allowing us to quantify and visualize partisan separation in semantic space. This approach enables us to assess whether the videos shown to Democratic- and Republican-aligned accounts are meaningfully distinct in content, or whether they largely occupy overlapping regions of the embedding space. As shown in Figure 7D, the cluster centroids of conditioning-stage videos seen by Democratic-aligned and Republican-aligned bots were approximately equidistant from the centroid of neutral videos, both in terms of their projected positions along the axis of maximal variation (0.05 vs. 0.032), as well as the average Euclidean distance between the individual videos belonging to that cluster and

the Neutral centroid in the original high-dimensional embedding space (0.446 vs. 0.443, $t = 1.962$, $p = 0.05$)

Taken together, these analyses suggest that the seeding videos shown to Democratic- and Republican-aligned bots were largely equivalent in both content and semantic distribution. Across multiple validation checks, including election relevance, partisanship framing, audience partisanship in the form of comments, and transcript-level semantic similarity, we find no consistent evidence of systematic differences between the two groups. This is in addition to the pre-seeding matching steps taken to ensure that videos were selected from channels of a similar overall popularity. This supports the integrity of the experimental design by confirming that any partisan divergence observed in later stages is unlikely to be attributable to initial seeding differences."

[1] - Wang, Liang, et al. "Multilingual e5 text embeddings: A technical report." arXiv preprint arXiv:2402.05672 (2024).

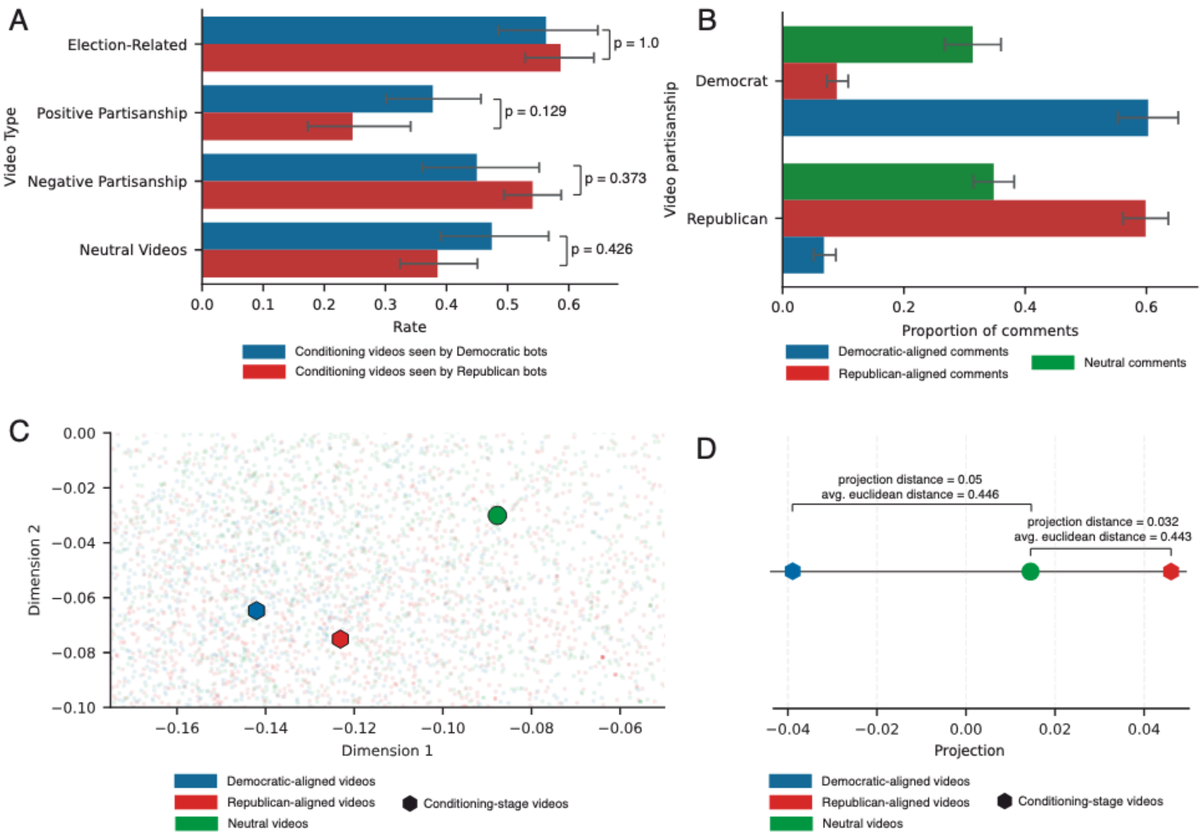

Figure 7: **Conditioning stage validation.** (**A**) The proportion of videos relating to the election, in support of a party, in opposition of the opposite party, or politically neutral in tone seen by Democratic-aligned and Republican-aligned bots. (**B**) The proportion of comments that are neutrally-aligned, democratic-aligned, and republican-aligned in videos seen by Democratic-aligned and Republican-aligned bots. (**C**) Two dimensional PCA mapping of the embeddings of the transcripts of Democratic-aligned, Republican-aligned during the conditioning-stage, and neutral videos during recommendation stage. (**D**) The projection of cluster centroids along the axis of maximal variation between Democratic-aligned and Republican-aligned videos.

As to your point stating that "There's no reason to necessarily think that TikTok is grouping users into "Democrat" and "Republican" clusters and making recommendations to them asymmetrically.", thank you for pointing out this possibility. Indeed, until a recent update in 2022, Meta allowed advertisers to specifically target Facebook and Instagram users by their political affiliation [1], showing that social media platforms do have the capacity to group users into clusters based on their preferred political party. Again, while we are hesitant to state explicitly that the asymmetries we observe are due to an intentional amplification of Republican content by TikTok, it is certainly a possibility based on the information social media platforms demonstrably cluster users by. We now mention this possibility in our manuscript by adding the following sentences:

> “This is especially of concern given that social media platforms have demonstrated the capacity to cluster users by their preferred political preference. Indeed, until a recent update in 2022, Meta, for instance, allowed advertisers to specifically target Facebook and Instagram users by their political affiliation [1].”
>
> [1] - https://www.facebook.com/business/news/removing-certain-ad-targeting-options-and-expanding-our-ad-controls

3. Data limitations
The authors also face two key data limitations that make it extremely difficult to make convincing inferences about the quantities they wish to estimate. These limitations are of course not their fault, but still raise questions about the claims that the authors offer.

The first is that they do not have access to dwell time, which is reportedly the key video-level metric of engagement that TikTok’s algorithm seeks to optimize. They instead use accessible “video-level metrics such as a video’s likes, likes per recommendation, comments, comments per recommendation, plays, and share count.” These are surely correlated with dwell time but they are not the same. The sensitivity analysis seems to help address this concern (though see below), but the absence of dwell time statistics means we can’t rule it out as an explanation for the observed differences in recommendations. In particular, the authors dismiss an argument about asymmetric homophily using (insignificant differences in) comments on videos (see below) but dwell time is surely the most relevant metric here.

In addition, the authors’ video-level analyses are conducted only among the videos that were recommended to the sock puppet accounts and not the true population of posts or a random sample of it. As a result, it is not possible to rule out the possibility that the observed asymmetries reflect an underlying imbalance in the supply of partisan content on the platform. This problem applies to every video-level datapoint (including the comments analysis, the transcripts, etc.).

Response:

We agree that not having access to *dwell time*, a key engagement signal for TikTok, is a limitation. We now explicitly acknowledge this in the Discussion and have added a paragraph to our Limitations section explaining that our engagement-based robustness checks are necessarily proxies. Indeed, we address this specific concern in the “Sensitivity Analysis” section of the study, where we show that any unobserved metric (such as dwell time in this case) would need to produce a gap 9.4 times larger than the gap in combined comments/likes/plays/shares seen between Republican and Democratic videos in order to explain the ideological skew which we observe. Ultimately, while this is potentially possible, we argue that this is unlikely to be the case. Nonetheless, we emphasize dwell time as a potential key engagement signal for TikTok which we cannot observe directly in our experimental setup. We add the following text to the relevant paragraph:

“While we have shown evidence that observable engagement metrics and viewership patterns cannot explain the ideological skew in Republican-aligned content on TikTok, we

cannot rule out the possibility that there are unobserved or latent engagement metrics or viewership patterns, known to the TikTok algorithm but unknown to us, that would explain the gap we observe. For instance, a key engagement metric such as "dwell time", or how long a user spends watching a particular video, is not a publicly available metric published by TikTok. As such, we conduct a sensitivity analysis to calculate how strong such an unobserved factor would have to be to fully explain TikTok's apparent ideological skew."

Respectfully, we think that our results in Table 1 and Figure 2 *do* rule out the possibility that observed asymmetry is a result of an overall imbalance of partisan content on TikTok. For example, if TikTok is providing recommendations as a function of the overall partisan content, we would expect users whose content we seed with homogeneously partisan content would *all* revert toward the overall partisan balance; instead, we observe that both Democratic-seeded and Republican-seeded accounts shift toward their respective party's content, whereas our neutral bots do not move at all.

4. Clustering

The reported results treat each recommended video as independent but of course the recommendations are surely correlated within bot and within a given time period. We recommend implementing two-way clustering by bot and week in all statistical analyses.

Response:

All of the regression results included in the manuscript now include standard errors clustered by bot and week. This change is also reflected in the manuscript text when we discuss our results and uncertainty.

5. Discrepancies in pre-registration and tables/figures

-"We also exclude pairs of bots that watched fewer than 150 videos each during the recommendation stage to allow for a sufficient number of political videos to be recommended, bearing in mind that not all recommendations are political in nature" (page 6 of paper) vs. " We will exclude any bot instances that do not meet the 500 recommended video threshold due to data collection errors." (preregistration).

Response:

Thank you for bringing this to our attention. Since the preregistration was submitted prior to any analysis being conducted, we unfortunately overestimated the number of videos bots would watch during an audit, causing us to reduce the threshold set on number of videos watched. We now explicitly state this deviation from our pregistration in the corresponding section of our results, as well as in the Methods section. The following sentence is added in both sections.

> *"This threshold is a deviation from our preregistration, which set the threshold at 500, due to our initial overestimation of the number of videos bots would watch during a given experimental run."*

-The preregistration states that “The first hypothesis is that there will be significant differences between the recommendation rates of partisan political videos made to the bots who are located (via VPN) in projected republican states and those in projected democratic states” but the paper does not formally test for differences between states.

Response:

Thank you for bringing this to our attention. We’ve now added commentary in the paper regarding this hypothesis, stating that there were no significant differences between bots in Texas and bots in New York with regards to the rates of partisan political recommendation rates (*p = 1.0)*. We’ve added the following to the results section:

> *“Comparing political recommendation rates broadly across states, we find that political videos were most frequently recommended in Georgia, followed by New York and Texas, although the differences in these rates across states were not statistically significant (NY vs. Texas: p = 1.0; NY vs. Georgia: p = 0.18; Georgia vs. Texas: p = 0.12).”*

-No code is provided to generate Figures 5 or 7 or the tables.

Response:

The code to generate all of the figures in our manuscript, as well as the data underlying each of the tables, have been uploaded to the Github repository.

-For Figure 2H (page 12), why are log odds centered at 1? Shouldn’t be at 0?

Response:

Thank you for catching this error. This is now adjusted in each of the figures and in the main text.

6. Other missing data issues

Sensitivity analyses on transcript availability and bot data missingness would be helpful as both raise selection bias concerns despite transcript availability seemingly not being associated with political leaning.

Response:

Thank you for this suggestion and we absolutely agree that these two aspects should have been included. We’ve now included the proportion of videos that included a transcript in a given audit as a covartiate in each of our regressions. In addition, to address bot data missingness, we’ve included the number of videos watched by a bot during its recommendation stage as a covariate. Indeed, bots with more missing data (i.e., those which failed earlier than others but still met the criteria for inclusion) watched fewer videos, hence, the number of videos watched captures bot data missingness. That being said, the point estimates for both of these new covariates are not statistically significant and very close to 0. We’ve also updated the text to mention our inclusion of these covariates based on your suggestion.

7. Other points:
-How much should we worry about multiple comparisons problems here? Should the authors be using a false discovery rate correction?

Response:

We believe that false-positive rate corrections are less helpful in our context. Normally researchers prioritize true positives, and false positives are the key threat. In our paper we are primarily *ruling out* alternative explanations, and so false negatives are the key threat. Nonetheless, we have now implemented a multiple testing correction in our key regressions (Figure 2, 3, and 4) and we find that our primary coefficient on bot conditioning remains significant. We also mention in the text that Bonferroni multiple comparison corrections were made.

-Non-statistically significant differences in the proportion of Republican comments on Democrat posts and vice-versa are reported as “meaningful”. The authors may be able to improve precision by increasing the sample size from 200 comments.

Response:

Thank you for your suggestion. We have now run the same pipeline using up to 500 comments per video and find largely similar results, with the result on the proportion of Neutral comments now being significantly different between Republican and Democratic-aligned videos. We’ve also used these updated comment mismatch rates within our remaining analysis including the key regression figures. We’ve updated the text describing these changes as follows:

> “*To test this, we collect a random sample of 500 comments from all political videos examined in our study and classify the content of each comment in the context of that video.*
>
> ...
>
> *We find meaningful differences between Republican-aligned and Democratic-aligned videos in the composition of comments, but not in the expected direction. We find that Democratic-aligned videos have a higher proportion of Republican-aligned comments than vice versa (12.9% versus 8.5%, χ^2 = 2.32, p = 0.13), as well as a higher proportion of copartisan comments than Republican-aligned videos (63.1% versus 51.6%, χ^2 = 1.97, p = 0.16). However, Republican-aligned videos have a significantly higher proportion of ``neutral'' comments that are not explicitly partisan (44.6% versus 26.3%, χ^2 = 7.53, p = 0.006)*”.

-The sock puppet accounts are set to a random age in the range 22-24. Given that age is likely a factor in TikTok's recommendation algorithm, this narrow and specific range limits the general interpretation of the findings. The authors should clarify this limitation and avoid overgeneralizing.

Response:

Thank you for pointing this out. We now state this as a limitation both in the Limitations section and in the Methods section where we describe this choice. Specifically, in the Methods section, we add the following:

> *"and assigning an age between 22 and 24. This allowed each account to impersonate a potential voter for the U.S. presidential election likely to be active on TikTok. This age range was also an intentional decision made to standardize the age of the user across experimental conditions. Moreover, our decision to select this age range was guided by data suggesting that the 18-24 age bracket had the largest share of users on TikTok in the United States as of 2022 (35%), although the older 25-34 age bracket now occupies the largest share as of late 2024 [1]."*
>
> *[1] - TikTok User Age, Gender, & Demographics (2025) --- explodingtopics.com. https://explodingtopics.com/blog/tiktok-demographics*

And in the Limitations section, we add the following:

> *"We also note that all bot accounts were set to a standardized age range of 22 to 24 years old. This decision was intentional, aimed at minimizing variability across bot profiles and aligning with a typical young adult TikTok user. However, this design choice limits the generalizability of our findings to users outside this age range, particularly younger adolescents or older adults, whose algorithmic experiences may differ."*

-The authors put a lot of effort into testing for state-based differences but end up finding few differences by state. They should make this point more clearly in the main text and explain to the reader when and why they pool their data versus separately analyzing data by state. It would be helpful to specifically justify why the neutral Georgia bots are the relevant point of comparison for the partisan bots.

Response:

We now better clarify throughout the Results sections where our data is pooled by week, state, and/or device. We also clarify our choice of including a neutral condition in Georgia (as opposed to adding neutral conditions in New York and Texas as well).

Thank you once again for your review and constructive feedback, and we hope that our responses have successfully addressed your concerns.

Referee #5 (Remarks to the Author):

In “TikTok’s recommendations skewed towards Republican content during the 2024 U.S. presidential race,” the authors report the results of an ambitious study of partisan skew in TikTok’s recommendation algorithm. The study is primarily comprised of a large-scale experiment in which hundreds of sock puppet accounts (i.e., bots) are seeded by watching a set of videos with partisan skew (except for a small number of ‘Neutral’ accounts in the control condition). The authors then collect and analyze information about the videos that were subsequently recommended by TikTok’s algorithm. They find a partisan skew in these recommendations, whereby accounts seeded with Republican content more consistently received more partisan content than those seeded with Democratic content. Moreover, in a result that is currently very much undersold in the paper, they find that this effect primarily originates from content that facilitates negative partisanship – that is, Republicans see more anti-Democrat content than Democrats see anti-Republican content, with positive partisanship varying little across the two partisan groups.

The study also includes several other analyses, including models that attempt (and ultimately fail) to explain the ideological skew from engagement metrics, topic models of the recommended content, an ‘exploratory’ misinformation analysis, and an original survey of TikTok users that aims to measure their perceptions of how political/partisan is the content in their feeds. Unfortunately, none of these other analyses are motivated at all in the front end of the paper (i.e., in the introduction or literature review), which makes for a difficult read. That said, there is a lot on the whole to like about the paper, from the experimental setup to the thorough discussion of the results, but the organization of the paper currently requires a lot of restructuring before it is published in a journal such as Nature.

One of the most striking missteps of the paper is the framing of the study as testing the effect of user behavior vs. algorithmic curation. While this is no doubt an interesting puzzle, and one that is exceedingly difficult to provide a comprehensive answer to, the authors simply do not (and, given the experimental setup, cannot) provide such an answer. This primary reason for this is that the experimental setup seeds each of the sock puppet accounts with partisan content, which is very much user behavior, so that any downstream effects on algorithmic recommendations simply cannot be assumed as being independent of user behavior. Moreover, it is unclear what content was used to seed these accounts as Republican or Democrat (vs. Neutral), so that the downstream ideological skew does not clearly have something to do with the algorithm but could instead results from differences in how ideologically extreme or centrist the initially seeded content is (more on this below).

As such, while the data collection and analysis efforts of the team are very impressive, the motivation for the study falls significantly short. I do not believe that this is a paper about user behavior vs. algorithmic swaying but rather a paper about negative partisanship in algorithmic recommendations – and to a lesser extent, a paper on ideological skew, if the authors can make a convincing case that this ideological skew does not result from a skew in how ideologically

extreme the seeded content is. If, for example, the content seeded for Republican sock puppet accounts was more ideological extreme, or more likely to feature negative partisanship, than content seeded for Democratic sock puppet accounts, then it would seem plausible that the downstream effects for algorithmic recommendations originate from this initial seeding asymmetry and not something about the algorithm (aside from the its tendency to recommend more of what the user has already viewed).

Response:

We thank you for pointing out this important concern, and we agree that further clarification on the videos which our bots were seeded with would greatly strengthen confidence in our findings. Our design cannot fully disentangle the influence of user behavior from algorithmic curation. Indeed, such disentanglement remains one of the central empirical challenges in this domain. However, our aim was not to decisively separate these forces, but to evaluate whether partisan asymmetries in recommendations emerge even when user behavior is carefully standardized across experimental conditions. By matching seeding channels based on engagement metrics and seeding bots with equivalent quantities of partisan content, we are able to observe how the algorithm responds to *comparable* user inputs. In an ideal world, we would be able to randomly toggle individual accounts' internal TikTok flags from Liberal to Conservative and back, and observe how the content TikTok feeds those accounts changes based on that toggle. Our experiment attempts to do the next best thing, which is randomly assigning those toggles at the start by feeding partisan content into the user's watch history. We also reinforce our findings with a broad array of robustness checks and sensitivity analyses, systematically ruling out any alternative pathways from user behavior.

That being said, we do agree that we cannot claim algorithmic effects are *completely independent* of user behavior. In our revised manuscript, we argue that our design offers valuable evidence that partisan asymmetries persist even when the behavioral "input" is symmetric. To further clarify this, we have revised our framing throughout the manuscript and removed language suggesting a clean causal separation between behavior and algorithm. We also now more clearly document the partisan balance of our seeding content in the "Conditioning Stage Validation" section of the Methods (see our response to your fifth comment).

Addressing these points would require substantial revisions to current the manuscript, and especially the frontend sections. However, given the unique and interesting data that the authors have collected, I would be happy to review a revised manuscript that addresses these points. Below, I include several other points that I hope will help the authors as they continue making revisions to the manuscript.

Response:

We would like to thank you for your careful reading of the paper and your constructive feedback which we believe has allowed us to improve our study and increase confidence in our

results significantly. We hope that our responses below address each of your concerns and we look forward to hearing any additional feedback that you may have.

1) The work cited by the authors that argues online news consumption is more diverse than offline news consumption is over 10 years old. Given the changes we have seen to digital platforms and the drifting of systems more generally it would be nice to have some more recent evidence or to cite additional evidence that supports this general point.

Response:

Thank you for pointing this out. We have now added more recent references to support this claim.

2) I am confused about why the authors suggest that the ‘weak tie phenomenon' does not happen on TikTok. If weak ties introduce users to more diverse content, wouldn’t strangers have a high chance of doing the same? Or is there a different weak tie phenomenon that the authors are referring to?

Response:

We agree that our argument here was not entirely appropriate and have removed this claim from the manuscript.

3) It is not clear why the authors spend so much time discussing the recent TikTok ban. While I understand that this is the platform they are using for data collection, the whole section has little to do with the research they are conducting and does not appear to provide any necessary context for understanding the method or results.

Response:

While we continue to believe that the TikTok ban and conversations in government and media surrounding it speak to the importance of our topic, we agree that it is not central to our argument and detracts from the narrative arc. We have now moved the parts of the section titled “Background: TikTok” discussing the geopolitical context and the TikTok ban to the Supplementary Materials giving the reader the opportunity to read that information if it is of interest to them.

4) The decision to have 7 experimental conditions, instead of 9 experimental conditions, is odd. While Dem-seeded and Rep-seeded accounts were allocated to the different states (NY, TX, and GA), Neutral-seeded accounts were only allocated to GA but not NY or TX. So, if one wants to asses the effect of partisanship for a NY-Dem account, the baseline would be GA-Neutral and not NY-Neutral, which obfuscates whether the difference is the state or partisan alignment. It seems useful to have NY-Neutral and TX-Neutral accounts to assess whether differences are due to the state or partisanship of the account. Some of this could be circumvented by leveraging GA-Dem to pick up the (Dem specific) state effect, but to get at the (NY specific) party effect we would need to assume that the difference between GA-Neutral and GA-Dem is the same as NY-Neutral and NY-Dem (since we cannot explicitly measure the latter). Why did

the authors not include a control group for each state? In general, it is better to compare two treatments to a control rather than to one another.

Response:

We agree it would have been better if our original design included 9 conditions. However, we quickly discovered that 9 conditions was prohibitively more difficult than 7 conditions from the perspective of the additional hardware required to run more simultaneous audits. As such, we prioritized the neutral condition in the swing state rather than the partisan strongholds. Part of this also arises from the technical concern that adding additional phones running simultaneously would increase the chances of our bots being flagged by TikTok and disabled, invalidating more of our experimental audits. We now mention this as a limitation in the Discussion. Specifically, we have included the following in the Methods section:

> *"While our original design contemplated a fully crossed 3 (State: New York, Texas, Georgia) × 3 (Partisan Seed: Democrat, Republican, Neutral) structure, we ultimately implemented a reduced version with seven conditions. In particular, neutral-seeded accounts were deployed only in Georgia. This decision was driven by practical constraints: expanding to nine concurrent experimental conditions required more devices to run simultaneously, which increased the risk of detection and deactivation by TikTok's platform safeguards. We prioritized the inclusion of a neutral baseline in Georgia, a prototypical swing state, given its strategic value for interpreting partisan effects in a politically heterogeneous context. While this design does not allow for full within-state comparisons between neutral and partisan accounts in New York and Texas, we note this as a limitation and interpret state-partisanship interactions with appropriate caution."*

In addition, we've included the following in the Limitations section:

> *"Our experimental design also required some concessions due to practical constraints. Ideally, the inclusion of a Neutral seeding condition in New York and Texas would have provided more accurate comparison baselines for the partisan-seeded audits in those states. However, expanding our experimental design to include an additional six audits per week came with the tradeoff of additional risk of detection and deactivation by TikTok's platform safeguards."*

5) One concern I mentioned above is the selection of initial content that was seeded. In particular, one would expect an ideological skew in algorithmic recommendations if left-leaning seeded content was centrist, on average, while right-leaning seeded content was extreme towards the right. Can the authors speak to the ideological differences of the seeded videos, as well as the extent that they feature positive vs. negative partisanship cues? One additional difficulty here is that right-leaning content may just be more extreme than left-leaning content, on average, and so the results are an artifact of this initial skew (rather than the alternative hypothesis, which is that the TikTok algorithm sways right-leaning users more to the right more than left-leaning accounts to the left). Of course, ultimately this conflates exactly what the

authors are claiming to disentangle – the decisions of users (and how extreme is the initial content they consume) vs. algorithmic swaying.

Response:

We thank you for raising this critical point about the potential confounding role of ideological extremity in the initial seed content. We agree that ruling out systematic differences in the nature of seeded videos is crucial to preserving the internal validity of our experimental design. In response, we have now added a new section in the manuscript titled "Conditioning Stage Validation" to the Methods (see page 37, Figure 7), which presents a comprehensive set of analyses to assess whether Democratic- and Republican-aligned bots were exposed to semantically and ideologically comparable content during the seeding phase.

First, we compare the proportion of videos directly related to the U.S. election or major political figures and find no significant difference across partisan groups. Second, we analyze the prevalence of positive versus negative partisanship framing in the seed content and again find no significant differences. Third, we use comment-level analysis to evaluate the partisan leanings of video audiences, which were balanced across bot conditions. Finally, we embed the video transcripts in a high-dimensional semantic space and show that the centroids of the Democratic and Republican seed content clusters are nearly equidistant from neutral content—both in terms of their projected location and Euclidean distance—suggesting semantic parity in initial exposure.

Taken together, these checks provide convergent evidence that the seed videos viewed by bots across partisan alignments were comparable in their issue relevance, affective tone, audience reception, and semantic content. We have updated the manuscript accordingly and explicitly highlight these findings in the new section. This additional validation strengthens our confidence that downstream asymmetries observed in the recommendation stage are unlikely to be driven by skewed initial inputs.

We copy below the new section added to the manuscript:

> *"We take several steps to validate that the seeding delivered to bots of opposite party alignments was equivalent. A summary of this analysis is presented in Figure 7. First, we evaluate the proportion of videos directly related to the U.S. election or major political figures (as determined by the second question of the transcript classification pipeline described above). As shown in Figure 7A, both Democratic-aligned and Republican-aligned bots were exposed to a statistically similar proportion of election-related videos (t = -0.478, p = 1.0). Regarding positive and negative partisanship, we also find no statistically significant differences in the proportion of such videos between the two bot groups (Positive-partisanship: t = 2.19, p = 0.129; Negative-partisanship: t = -1.7, p = 0.373; Neutral: t = 1.63, p = 0.426).*

Second, we classify a random sample of up to 500 comments posted under each of the partisan videos seen during the conditioning stage, using the comment classification procedure described in the previous section. As shown in Figure 7B, videos shown to Democratic- and Republican-aligned bots received similar rates of both party-aligned and opposite-party-aligned comments (Party-aligned comments: 60.2% vs. 59.9%, χ^2 = 0.008, p = 0.925; Opposite-party-aligned comments: 8.9% vs. 6.8%, χ^2 = 0.18, p = 0.67).

Third, we compute the embedding of the transcripts of all videos within our dataset using the embedding model proposed by Wang et. al [1], chosen as an open-source embedding model with performance on par with state-of-the-art models. We then compute the centroids of the sets of videos seen by Democratic-aligned and Republican-aligned bots during the conditioning-stage of their respective audits. The projection of these cluster centroids into two dimensions using PCA is illustrated in Figure 7C. To better understand the extent to which the sets of videos diverge semantically across partisan conditions, we compute the axis of maximal variation between Democratic and Republican videos by taking the unit vector connecting their respective centroids in the original embedding space. We then project the corresponding centroids onto this axis, allowing us to quantify and visualize partisan separation in semantic space. This approach enables us to assess whether the videos shown to Democratic- and Republican-aligned accounts are meaningfully distinct in content, or whether they largely occupy overlapping regions of the embedding space. As shown in Figure 7D, the cluster centroids of conditioning-stage videos seen by Democratic-aligned and Republican-aligned bots were approximately equidistant from the centroid of neutral videos, both in terms of their projected positions along the axis of maximal variation (0.05 vs. 0.032), as well as the average Euclidean distance between the individual videos belonging to that cluster and the Neutral centroid in the original high-dimensional embedding space (0.446 vs. 0.443, t = 1.962, p = 0.05)

Taken together, these analyses suggest that the seeding videos shown to Democratic- and Republican-aligned bots were largely equivalent in both content and semantic distribution. Across multiple validation checks, including election relevance, partisanship framing, audience partisanship in the form of comments, and transcript-level semantic similarity, we find no consistent evidence of systematic differences between the two groups. This is in addition to the pre-seeding matching steps taken to ensure that videos were selected from channels of a similar overall popularity. This supports the integrity of the experimental design by confirming that any partisan divergence observed in later stages is unlikely to be attributable to initial seeding differences."

[1] - Wang, Liang, et al. "Multilingual e5 text embeddings: A technical report." arXiv preprint arXiv:2402.05672 (2024).

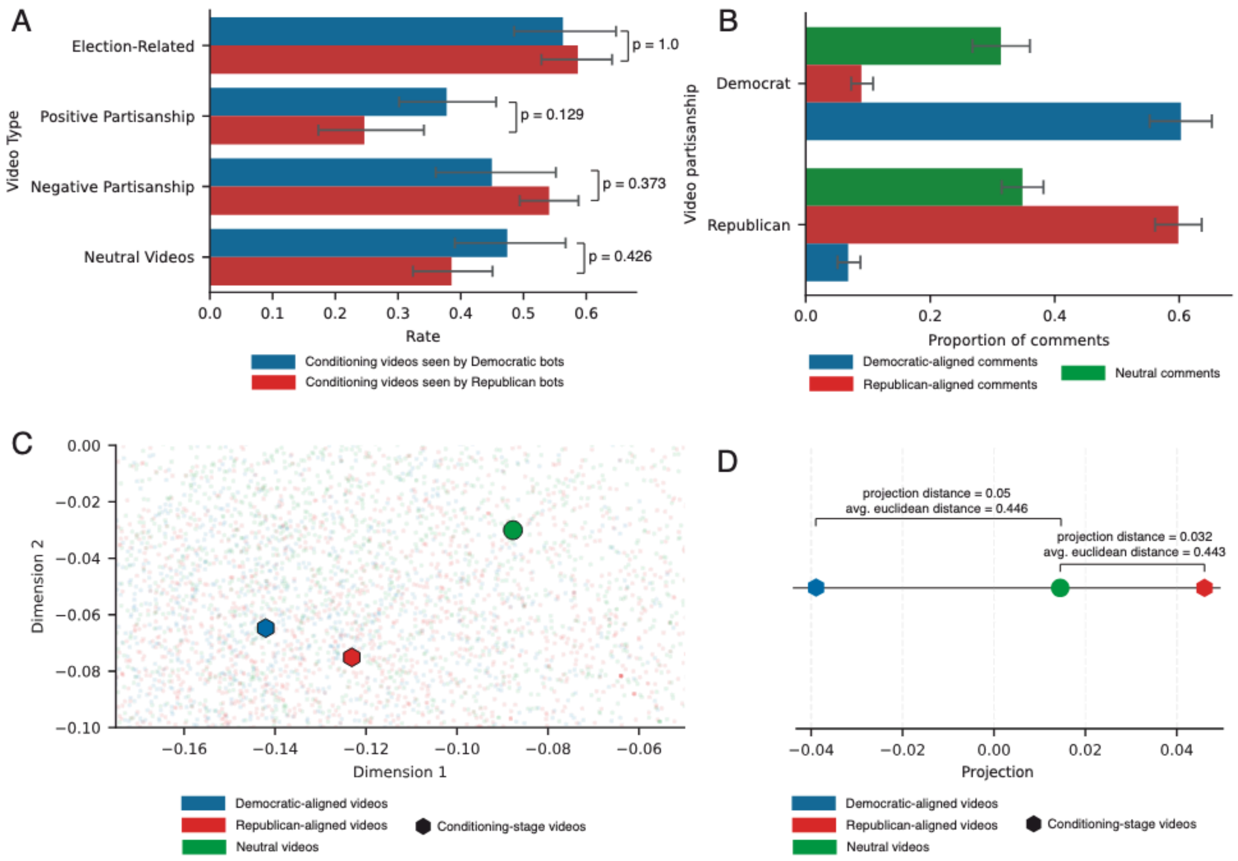

Figure 7: **Conditioning stage validation.** (**A**) The proportion of videos relating to the election, in support of a party, in opposition of the opposite party, or politically neutral in tone seen by Democratic-aligned and Republican-aligned bots. (**B**) The proportion of comments that are neutrally-aligned, democratic-aligned, and republican-aligned in videos seen by Democratic-aligned and Republican-aligned bots. (**C**) Two dimensional PCA mapping of the embeddings of the transcripts of Democratic-aligned, Republican-aligned during the conditioning-stage, and neutral videos during recommendation stage. (**D**) The projection of cluster centroids along the axis of maximal variation between Democratic-aligned and Republican-aligned videos.

6) My major takeaway from the section on negative partisanship (and especially Figure 4) is that the source of the ideological skew identified in the previous section is entirely negative, not positive, partisanship. In particular, Pro-Democrat content for Democrats was just as likely as Pro-Republican content for Republicans. However, Anti-Democrat content for Republicans was MUCH more likely than Anti-Republican content was for Democrats. This seems like a major result, and yet it is currently difficult to decipher from the section on negative partisanship (I only realized it from studying Figure 4). I believe this finding likely belongs in the title of the paper and should be discussed much earlier and more thoroughly in the frontend (intro and lit review) sections. There is existing work on e.g., outgroup animosity

(https://www.pnas.org/doi/10.1073/pnas.2024292118) and negativity (https://www.nature.com/articles/s41562-023-01538-4) that the authors can leverage here.

Response:

We completely agree with your insights here and have addressed these point in the following ways:

1. We have modified the title of the paper to *"TikTok's recommendations skewed towards Republican content and negative-partisanship during the 2024 U.S. elections"*

2. We've included our findings on negative partisanship to the abstract by adding the following sentence:

   "We find significant asymmetries in content distribution: Republican-seeded accounts received ~11.8% more party-aligned recommendations compared to their Democratic-seeded counterparts. Moreover, Democratic-seeded accounts were exposed to ~7.5% more opposite-party recommendations on average, primarily driven by negative-partisanship content, i.e., content in criticism of the Democratic party. These asymmetries exist across all three states and persist when accounting for video- and channel-level engagement metrics such as likes, views, shares, comments, and followers, and are driven primarily by negative partisanship content."

3. We have revised the introduction to foreground the literature on outgroup animosity, adding the two references you have suggested.

   "Beyond examining the overall political orientation of TikTok's recommendations, we also investigate the type of partisanship these videos reflect. Partisan content can be positive, promoting a given political party, or negative, attacking the opposing party. Prior work suggests that negative partisanship, or out-group animosity, is a key driver of online engagement. For instance, Rathje et al. [1] show that out-group animosity is the strongest predictor of sharing behavior on Facebook and Twitter, while Robertson et al. [2] find that negativity in news headlines significantly boosts consumption. However, it remains less clear whether such negativity is amplified by algorithmic recommendations in environments where user agency is limited, such as on TikTok."

   [1] - S. Rathje, J.J. Van Bavel, & S. van der Linden, Out-group animosity drives engagement on social media, Proc. Natl. Acad. Sci. U.S.A. 118 (26) e2024292118, https://doi.org/10.1073/pnas.2024292118 (2021).

   [2] - Robertson, C.E., Pröllochs, N., Schwarzenegger, K. et al. Negativity drives online news consumption. Nat Hum Behav 7, 812–822 (2023). https://doi.org/10.1038/s41562-023-01538-4

4. We have clarified the commentary around the results presented in Figure 4 to better communicate our findings by adding the following two paragraphs:

"Indeed, negative-partisanship videos demonstrated higher absolute engagement (views, likes, comments) on average compared to positive partisanship videos. However, when controlling for viewership through per-view analysis, engagement rates were largely comparable between two categories, with only likes-per-view remaining significantly higher for negative partisanship content ($p = 0.0026$; see Supplementary Table 22). These results align with broader patterns observed on Facebook and Twitter which suggest that videos with "out-group animosity" receive more engagement [1].

Looking at ideologically mismatched content specifically, we find that Democratic-conditioned and Republican-conditioned bots were almost equally as likely to see positive-partisanship videos of the opposite party (Pro Republican, 2.9%, and Pro Democratic, 2.3%, videos, respectively, $\chi^2 = 4.94$, $p = 0.03$). However, Republican bots were far less likely to see Anti-Republican content than Democratic bots were to see Anti-Democratic content (5.8% and 14.2%, respectively, $\chi^2 = 234.25$, $p < 0.001$), suggesting that the ideologically mismatched recommendations were driven specifically by negative-partisanship videos."

[1] - S. Rathje, J.J. Van Bavel, & S. van der Linden, Out-group animosity drives engagement on social media, Proc. Natl. Acad. Sci. U.S.A. 118 (26) e2024292118, https://doi.org/10.1073/pnas.2024292118 (2021).

7) If the authors are planning to include the analyses on topic modeling, misinformation, and/or user perceptions (via the survey) then they should make motivate these analyses earlier on by using relevant literature that frames why we should care about these results. As of now, these sections come out of the blue and it is unclear what contribution these analyses make to our understanding of political behavior or digital media. The section on misinformation is especially problematic, as it relies on cosine similarity matching of sentence embeddings which strays in a very significant way from how misinformation is detected in other studies. It is, of course, quite possible that this technique is used, but the authors need to convince the reader that this is a reasonable analysis. I know this is mentioned in the limitations section, but that is not enough to justify keeping that particular analysis in the paper as it is not even motivated in the frontend sections of the paper. I believe the authors should either keep these analyses but properly motivate them or leave them out entirely and focus instead on the experiment that is central to the paper.

Response:

Thank you for your suggestion. We agree with you that these analysis were not well motivated in the introduction. To address your concern, we have added more context regarding our motivation to include our topic modeling and user perception analyses. We have also removed the misinformation analysis from our article, as we completely agree that it lacked the necessary rigor to be included in the overall narrative of our study. Instead, we now point to the potential of studying misinformation on TikTok as an important avenue of future research.

We have now added the following paragraph to the Introduction which we believe better contextualizes our topic modeling analysis and user study within the broader literature.

> *"While our primary focus is on auditing TikTok's political content recommendations using controlled bot accounts, we complement this analysis with two additional components. First, we apply topic modeling to uncover the dominant themes and issues represented in recommended videos, providing insights into both the topics each partisan group encounters and how these topics are ideologically framed. This analysis adds to the broader literature regarding communication in political and non-political contexts on social media [1,2,3,4]. For instance, Barberá et al. [1] find that, despite the existence of ideological clustering on Twitter, users are often exposed to a diversity of political topics, including cross-cutting content from opposing viewpoints. Their findings suggest that analyzing what topics are encountered is just as important as how often partisan content appears, as the structure and framing of topic exposure may contribute differently to political polarization. In our context, topic modeling offers a means of assessing whether TikTok's recommendation system fosters similar issue diversity or instead narrows users' political information diets along partisan lines. Second, we incorporate a user survey to assess whether the asymmetries identified through our bot-based audit are perceptible to real users. While automated audits can reveal structural patterns in algorithmic recommendations, their real-world relevance depends on whether these patterns are experienced similarly by platform users. If users interpret their feeds as politically balanced or skewed in a different direction, it would suggest a disconnect between algorithmic behavior and user experience. By comparing our experimental findings with user-reported perceptions of political content on TikTok, we evaluate the extent to which observed asymmetries are not only algorithmically present but also socially meaningful."*

[1] Barberá, Pablo, et al. "Tweeting from left to right: Is online political communication more than an echo chamber?." Psychological science 26.10 (2015): 1531-1542.
[2] Stefanov, Peter, et al. "Predicting the topical stance and political leaning of media using tweets." Proceedings of the 58th Annual Meeting of the Association for Computational Linguistics. 2020.

[3] Sterling, Joanna, John T. Jost, and Curtis D. Hardin. "Liberal and conservative representations of the good society: A (social) structural topic modeling approach." Sage Open 9.2 (2019): 2158244019846211.
[4] Katalinić, Josip, Ivan Dunđer, and Sanja Seljan. "Polarizing Topics on Twitter in the 2022 United States Elections." Information 14.11 (2023): 609

8) An inherent limitation in the study design, which is mentioned in the limitations section, is that it captures a very limited time window for these sock puppet accounts. In particular, we see recommendations for an account that was only very shallowly trained with content, which of course does not capture what the algorithm is doing for the vast majority of actual accounts that are seeded with a lot more content than these sock puppet accounts. As such, what the study is picking up is ideological skew and negative partisanship in algorithmic recommendations that are made VERY EARLY ON in a user's time on the platform. It is not at all clear how much these results would generalize to how the algorithm works for users who have engaged with the platform for much longer. This seems like a pretty important limitation, yet the authors simply gloss it over in the limitations section. More discussion on the implications of this is warranted, and the authors should warn readers that we simply cannot use these findings to generalize to the typical TikTok user.

Response:

This is an important point, and we thank you for encouraging us to expand our discussion of it. We now make clear that our bots simulate early-stage interactions, where algorithmic influence may be most impactful in shaping users' initial media diets. We also add a new paragraph in the Limitations addressing how long-term exposure dynamics remain an open empirical question, and outline this as a direction for future research. Specifically, we have added the following to the Limitations section:

"Our design also only captures algorithmic behavior during the earliest stages of user engagement on the platform. Each bot account in our study interacted with a relatively small set of partisan videos before entering the recommendation phase. As such, our findings only reflect ideological skew and partisan dynamics in initial content curation, rather than long-term platform behavior. This choice is ultimately a tradeoff, allowing us to study changes in this initial content curation stage of a user's life cycle over the course of six months, at the expense of not tracking asymmetries which may occur with more long-tenured users. Understanding how ideological patterns evolve over a longer duration, and how initial skew compounds or attenuates with prolonged engagement, remains an important direction for future work."

Minor comments:

1) Christian Bail is a trained sociologist, not a trained political scientist (his book is listed as a reference in political science instead of sociology). Also, Vosughi et al. are not trained in

communication studies (with the possible exception of Deb Roy) – they are in computer science / information systems scholars.

Response:

Thank you for highlighting this point. We have updated the references to their correct classifications accordingly.

2) For channels with fewer than 10 videos, the authors supplemented with up to 30 additional videos from TikAPI. How was the partisan alignment of these additional videos measured? Did this also go through LLMs as with the other videos collected?

Response:

These videos were passed through the same LLM classification pipeline as our main dataset. We now clarify this in the manuscript by including the following sentence.

> *"... supplementing our dataset with up to 30 additional pre-election videos from the TikAPI for channels with fewer than 10 labeled videos in our sample. These additional videos were then passed through the same pipeline described in the previous section to discern their partisan alignment."*

3) It would be useful to see the top accounts that did not meet the 75% threshold to be assigned a partisanship label. I assume many of these are not political, but some may be political but less biased, and a breakdown based on whether or not these were deemed political by the LLMs would also be useful to see.

Response:

We examined 13 channels that fell between the 60-75% threshold. As anticipated, these channels largely comprised traditional news outlets (Daily Mail, USA TODAY, Channel 4) or talk shows (The Daily Show, Don Lemon) that cover political topics while maintaining some editorial balance. This analysis validates our decision to use 75% as a threshold, as it distinguished consistently partisan channels from those with occasional political lean. We have added this validation to the main text ("Channel Classification" in Methods) and provided the complete list of these channels in Supplementary Table 24.

4) It is weird to see IQR, which I assume is short for Interquartile Range, depicted as a single number rather than a range. How was this calculated? Also, the authors should mention this in the main text if they are reporting it in the table. They might also add a note about how total (sum) likes across the top 20 accounts skewed towards Democrats, even though median likes skewed towards Republicans.

Response:

We now report the Interquartile Range as a range rather than a single number. Initially, it was reported as the difference between the 75th percentile and the 25th percentile of the respective metric, but we agree with you that this is less clear than reporting these values

directly as a range. We also add commentary on the IQR in the text discussing the Table. As for the discrepancy between the median number of likes and the total number of likes, we now better explain that this is due to Democratic-aligned channels having far more videos published than Republican-aligned ones. As such, even though Republican aligned channels tend to have more likes on average, they have fewer likes in total across all videos published. We make this clearer with the inclusion of the text below:

> *"Republican channels saw higher median engagement across followers and likes per channel compared to their Democratic-aligned counterparts, but had a smaller number of likes overall, due to having far fewer videos published on the platform. The interquartile range (IQR), reported as the range between the 25th and 75th percentile, showed greater variability among Republican channels for followers but greater variability among Democratic channels for likes, suggesting that top Republican channels generally received more consistent engagement rates over their Democratic counterparts."*

5) It is a bit unclear from the text what time represents. Each bot ran for a week, correct? So that the time effect implies that, as the election nears, there is a larger ideological skew in recommended videos for a new bot (rather than the alternative interpretation, which is that the skew for a given bot increased the longer they were on the platform). The authors might specify this alternative and highlight that this is *not* what's being measured, as this would seem to be important to study in future research (and, as far as I can tell, is the only way to address point 8 above - that the longevity of the bot accounts is tiny compared to the average user).

Response:

In the Results section, when discussing the recency scaling in our counterfactual models, we are now more explicit by what we mean by time, stating *"Specifically, we inversely scale each metric in question with the time between when an audit was conducted and a given video's publishing date, once linearly, and once exponentially."*

Lastly, with regards to your point both here and major point 8 above, we discuss in the Limitations section our design choice of having each bot run for a period of one week, rather than over an extended period of time. This, again, ultimately is a design tradeoff which allowed us to measure changes to the algorithm's behavior with new users week over week, as opposed to measuring changes to the same user's algorithm over a longer period of time. We point to how this remains an open question worthy of exploration.

6) Figure 5 should be moved to later on in the manuscript, after it has been discussed.

Response:

Figure 5 is now moved after the section in which it is discussed.

Thank you once again for your review and hope that we have addressed all of your concerns. We look forward to hearing any additional feedback you may have.

Response to the Editor

Dear Sebastian,

We are grateful for the opportunity to revise our manuscript, “TikTok's recommendations skewed toward oppositional and Republican-aligned content during the 2024 U.S. elections” We carefully considered your guidance and the reviewers’ comments, and we have made extensive revisions throughout the manuscript to strengthen clarity, precision, and focus. Below, we outline how we responded to each of the points raised.

1. Robustness and sensitivity checks

Several reviewers (R2, R3, R4) requested additional robustness analyses to better understand the sources of asymmetry. In response, we’ve made a number of modifications, all of which confirm our broad findings and further strengthen our evidence:

a. We more clearly explain our analyses stratifying recommendations by both party and negativity (Figure 4E; Supplementary Table 22).

b. We added a new regression predicting the likelihood of being recommended a negative-partisan video. The results show that video- and author-level features (follower count, share count, normalized comments), rather than bot conditioning, significantly predict negative tone, indicating supply-side rather than conditioning-stage effects (Supplementary Figure 7).

c. We explicitly discuss how asymmetries intensify as the election neared (Figure 2 A–C), showing that these patterns cannot be explained by conditioning differences alone and may reflect algorithmic changes or shifts in user content supply.

d. To address concerns about multiple testing, we now report unadjusted, Bonferroni-corrected, and Benjamini–Hochberg adjusted p-values in the relevant supplementary tables (Supplementary Tables 15–17), demonstrating that our substantive findings hold across correction methods.

2. Terminology clarity

a. We now define all terms explicitly and early in the “Measuring Political Content” section (p. 8). Specifically, we classify videos as Pro-Democratic, Anti-Democratic, Pro-Republican, or Anti-Republican, and derive four analytic constructs: positive partisanship, negative partisanship, co-partisan recommendations, and cross-partisan recommendations.

b. We clarify that we use negative partisanship to describe message tone (anti-party content), regardless of the viewer's identity, consistent with Iyengar & Ansolabehere (2010). For example, a Democrat viewing an Anti-Democrat video is coded as exposure to negative-partisan content because the content itself is oppositional.

c. We replaced "opposite-party recommendations" with the clearer term cross-partisan recommendations.

3. Limitations integrated across the manuscript

Reviewers requested that we foreground study limitations earlier in the manuscript (R2, R3, R4). To address this, we have:

a. Added explicit acknowledgment in the Experimental Setup section that unobservable asymmetries may remain in the conditioning stage because Democratic seed accounts were more often official office holders, whereas Republican seeds skewed toward independent creators. This could affect latent clustering, even though our validation showed no semantic or engagement differences.

b. Included this point again in the Limitations section to emphasize that while seed videos were balanced on observable features, structural account-level asymmetries remain possible.

c. Clarified throughout the manuscript—including in the Abstract, Introduction, and Political Recommendation Rates sections—that our design cannot isolate user behavior from algorithmic rules with certainty. We now consistently describe our findings as evidence of a Republican-leaning skew in recommendations unexplained by observed engagement metrics, rather than attributing them directly to algorithmic intent.

d. Revised the title and abstract to avoid jargon and causal overreach, and to foreground the main contributions more directly.

4. Remaining reviewer concerns

We made additional revisions to address specific points:

a. Narrative focus (R5): We streamlined the Abstract, Introduction, and Discussion, reducing their length by ~1,000 words and sharpening the narrative arc to emphasize our central contributions. Broader discussions of "social media harms" have been pruned or reframed to ensure they remain directly linked to our findings.

b. Title revision (R5): The title now reads: "TikTok's recommendations skewed toward oppositional and Republican-aligned content during the 2024 U.S. elections" This highlights the main findings clearly while avoiding undefined terms.

c. Conditioning-stage asymmetry (R5): We now explicitly acknowledge that while differences in positive vs. negative partisanship across seed sets were not statistically significant, the Republican seeds contained somewhat more anti-Democratic content. We clarify that this reflects the supply of content available for seeding, not deliberate selection, and that this may approximate real user conditions. We also test whether this imbalance predicts downstream recommendations and find that it does not (Supp. Figure 7).

d. Key effects clarified (R5): We now highlight two striking asymmetries in the abstract and discussion:

   i. Republican-seeded accounts encountered more anti-Democratic than Democratic-seeded accounts encountered anti-Republican content.
   ii. Democratic-seeded accounts saw more anti-Democratic content than Republicans saw anti-Republican content.

Through these revisions, we believe the manuscript is now substantially clearer, more precise, and more focused on its central contributions: (i) TikTok's recommendations exhibit a persistent Republican-leaning skew during the 2024 U.S. elections, and (ii) this skew is driven disproportionately by negative content targeting Democrats. We thank you and the reviewers for your constructive feedback, which has helped us significantly strengthen the paper.

Referee #1 (Remarks to the Author):

I am fully satisfied with the authors' responses and corresponding changes in the manuscript. I recommend the publication of this piece.

Response:

Thank you for your evaluation of our work! We are grateful for your kind words and that you are satisfied with our changes.

Referee #2 and #3 (Remarks to the Author):

We thank the authors for their response, which addressed many of our concerns. However, there are still a few outstanding issues that should be addressed before publishing. We therefore recommend another R&R.

Response:

Thank you for your supportive remarks and your careful reading of our revised manuscript. Below, we respond point-by-point to each of your remaining comments. We hope our responses adequately satisfy your concerns.

1. Why do the authors think this is happening?

The authors added the following passage to the Robustness checks section:

“However, if there are more Republican users or content creators on TikTok, if Republican-aligned content is more engaging, or if Democrats are more interested in Republican-aligned content than Republicans are in Democratic-aligned videos, then this ideological skew is exactly what we might expect TikTok's algorithm to produce. Alternatively, if user behavior cannot explain the asymmetries we observe, it would suggest that there are internal governing rules governing the algorithm’s behaviour that cause these results, independent of user behavior.”

We agree that this statement is helpful. However, it comes deep in the paper (page 13). There are several passages earlier which seem to attribute the findings to recommendation algorithms without acknowledging the inferential challenge the authors face. Here are a few examples from the paper that nudge the reader toward the algorithm explanation without acknowledging the points made in their response:

From the abstract: “These asymmetries … persist … and are driven primarily by negative-partisanship content. Our findings suggest that recommendation algorithms can play an important role in shaping users’ media diets.”

From “Our Contributions”: “Our findings reveal a significant Republican bias in TikTok’s recommendation algorithm” sounds like a settled verdict on the algorithm itself.

The authors also suggest they “isolate the causal role of algorithms” in the introduction.

The authors are much more clear about the design of the paper and the assumptions they have to make in their memo:

“Our goal was to study partisan asymmetries in TikTok’s recommendation algorithm, and indeed we found significant asymmetries in co-partisan and cross-partisan recommendation rates. There are only two plausible factors behind these asymmetries. Either user behavior has trained” the TikTok recommendation algorithm to produce partisan asymmetries, or else there are internal governing rules of the TikTok algorithm that have produce[d] this result independent of user behavior; these governing rules may be intentionally partisan or they may not. Importantly, if we find that the observed asymmetries cannot be explained by the user behavior, then the rules governing the algorithm must be Responsible.”

“In our study, we systematically rule out all plausible routes through which user behavior might result in the partisan asymmetry, including content type, channel- and video-level engagement metrics, and co-viewership networks, based on the available data and within the constraints of our experimental design. Our goal behind ruling out all those user-centered explanations was to strengthen our conclusion that the asymmetries could indeed be influenced, at least partially, by the algorithm’s governing rules.”

“Crucially, we are careful throughout the manuscript to avoid asserting that TikTok is intentionally promoting pro-Republican or anti-Democrat content. Nonetheless, our findings are of potential public concern, regardless of whether they stem from intentional algorithmic design choices made by TikTok. Indeed, an algorithm which asymmetrically recommends partisan content may have potential significant downstream implications on user behavior.”

We recommend that these points be clearly stated in the manuscript itself. They should be integrated throughout the paper (framing, intro, abstract, conclusion, etc) and not just on page 13. We also recommend against using the term “bias” as in the quote from the “Our Contributions” section above - the authors use the term “skew” elsewhere, which is preferable. A more precise alternative might be “Republican-leaning skew in recommendations that cannot be explained by the user engagement metrics we observed” (perhaps with a reference to the Robustness section).

Response:

Thank you for these points and we agree that our language in the front-end of the paper needed further adjustments for clarity on what exactly we are looking to measure. To address your concern, we’ve adjusted much of the causal language in the abstract, introduction and experimental setup sections to better reflect the limitations of our design. We’ve also replaced

the term ***bias*** with ***skew*** when discussing our own findings throughout the paper. The changes made to the front end of the paper can be seen below.

In the abstract we now write:

These asymmetries persist when accounting for video- and channel-level engagement metrics such as likes, views, shares, comments, and followers, indicating a Republican-leaning skew in recommendations that cannot be explained by the engagement measures we observe, suggesting that TikTok's algorithmic rules may play an important role in shaping users' media diets.

In the "Our Contributions" section, we adjust our language to the following:

"Our findings reveal a significant Republican-leaning skew in TikTok's recommendations—one that persists across states even after accounting for channel- and video-level engagement metrics. By systematically ruling out plausible user-centered explanations, we provide evidence that the asymmetries we observe may be influenced, at least partially, by the algorithm's governing rules, though not necessarily by intentional design choices.

…

While our design allows us to reduce the role of user self-selection, we cannot directly observe the platform's internal governing rules. Thus, we interpret our findings as evidence of partisan asymmetries in recommendations that cannot be explained by the user-driven mechanisms we measure, rather than as definitive proof of intentional algorithmic design."

In the "Political recommendation rates" section, we add the following:

While our design allows us to compare co- and cross-partisan recommendation rates, our findings cannot definitively determine whether asymmetries arise from user behavior training the system or from internal algorithmic rules. What we can show, however, is that the skews we document cannot be explained by observable user engagement metrics (see the Robustness Checks section below).

2. Alternative explanations for asymmetry

The authors use data to address concerns about possible asymmetries between the Democratic and Republican videos used to train Tik Tok algorithm. Their response cites insignificant differences in embedding space, comments on videos by party alignment, and rates of election-related videos and positive versus negative partisanship. These are all helpful in ruling out any obvious differences between the conditioning content within the limits of the available data. However, we still believe the authors should more explicitly warn readers that there may still be unobservable asymmetries in the conditioning stage. For example, unobserved account-level factors may be sorting users into groups that are not addressed in the new feed

condition validation section. Because eight of the twelve Democratic seed channels are official office holders and the Republican set skews toward independent creators, the follower communities attached to those accounts may differ systematically in ways that the authors' comment-level analysis cannot detect. If TikTok clusters users partly on latent engagement profiles, the bots could inherit those cluster-level characteristics despite comparable video semantics. While an account-level analysis is understandably out of the reach of the study, we believe it's important to acknowledge this point and make these limits of the analysis transparent.

Response:

Thank you for raising this point and we agree that this limitation should be acknowledged more explicity and earlier in our manuscript. We've now added the following to the Experimental Setup section (Page 5-6 of the manuscript):

"While our validation checks confirm that conditioning videos are comparable across partisan groups in terms of semantics, partisanship, and engagement features, unobservable asymmetries may remain. For example, eight of the twelve Democratic seed channels are official office holders, whereas the Republican set skews toward independent creators. If TikTok clusters users partly on latent engagement profiles, our bots could inherit such characteristics despite conditioning on otherwise comparable videos. An account-level analysis is beyond the scope of this study, but this limitation should be kept in mind when interpreting our results."

We also add the following to the Limtiations section:

"A further limitation concerns possible asymmetries in the conditioning stage of our audits. Although seed videos were comparable across partisan groups in semantics, partisanship, and engagement features, the source accounts differed: eight of the twelve Democratic seeds came from official office holders, while the Republican set skewed toward independent creators. If TikTok clusters users partly on latent account profiles or community structures, our bots could inherit such differences despite conditioning on otherwise comparable videos. This possibility should be kept in mind when interpreting our results."

The authors might also consider whether the asymmetry of concern is not positive vs. negative partisanship or Democrat vs. Republican but the interaction between negative partisanship and Republican alignment. As Figure 7A shows, the rate of positive partisanship is lower among R videos and the rate of negative partisanship is higher. Neither is significant but the difference in differences is potentially of concern. For instance, Republicans are more likely to share content from low quality news sources both among the mass public https://www.nature.com/articles/s41586-024-07942-8 and elites https://academic.oup.com/pnasnexus/article/1/4/pgac186/6695314. Do the authors observe evidence that anti-Democratic videos perform better? It would be helpful to stratify their analyses by negativity and party rather than just party to see if the results are robust and/or to estimate models with party X negativity interactions.

Response:

This is a great point and we absolutely agree that these interaction effects are worth studying. We originally included these interaction effects in our regression shown in Figure 4E, but we agree that we were not clear enough in our discussion of these results in the manuscript. We've now added the following text when discussing the regression shown in the Figure. We've also adjusted Supplementary Table 22 to include the average engagement metrics for each of the five video types we study (Pro/Anti Democrat and Republican, as well as Neutral).

"We further tested whether partisan asymmetries are driven specifically by the interaction between party and negativity. Figure 4E presents regression estimates where we stratify videos by both party and partisanship type. We find that Anti-Democratic videos stand out: they are significantly more likely to be recommended as mismatches than any other video type, even after controlling for engagement metrics and fixed effects. This confirms that the partisan skew we document is not simply Republican versus Democratic, or positive versus negative, but specifically concentrated in negative out-party content targeting Democrats."

7. Other points: Should the authors be using a false discovery rate correction?

The manuscript states that it applies Bonferroni adjustments (noted briefly in text and figure captions) but does not explain or justify that choice. Please briefly clarify the set of analyses to which you are applying Bonferroni adjustments and explain why they are preferred over other FWER or FDR methods (e.g., Benajmini–Hochberg). You may also wish to report both adjusted and unadjusted p-values so readers can gauge robustness.

Response:

Thank you for this suggestion. We are now more explicit as to which analyses we are applying multiple comparison adjustments to. For completeness, we now report our p-values after each of Bonferroni and Benjamini-Hochberg adjustments, as well as the unadjusted p-values in the relevant supplementary tables (Supplementary Tables 15, 16, and 17). We do note, however, that as Bonferroni corrections are usually considered the most conservative, none of our substantive conclusions are altered.

Thank you once again for your review of our manuscript and we hope that all of your concerns have been addressed!

Referee #5 (Remarks to the Author):

In their revised manuscript, “TikTok’s recommendations skewed towards Republican content and negative-partisanship during the 2024 U.S. elections,” the authors take several measures to address my comments on their previous draft. These efforts are commendable, and I appreciate that the authors have taken the time to review my comments and respond accordingly. The addition of the section on “Conditioning Stage Validation” is a very welcome step towards additional transparency in the study’s experimental manipulations. The removal of the section on misinformation is justified, given both that it relied on unusual methods for identifying misinformation and that the section was not motivated and served as somewhat of a distraction from the study’s main findings.

Response:

Thank you for your careful reading of our updated manuscript and for your constructive comments. We agree with your points and offer our point-by-point responses to each of your concerns below.

Unfortunately, I still believe that the paper suffers from too many issues to recommend publication. The main issue is that the discussion and, to a lesser extent, the analysis are too unfocused for a Nature article. There is too much discussion outside of the study’s main findings and this makes it difficult to decipher the main contributions of the article (of which there are several, but they currently get lost). Despite my previous comments, the authors still choose to frame the abstract and introduction as a test of user behavior vs. algorithmic recommendations. As I mentioned previously, and as the authors agreed in their rebuttal, the very manipulation that the authors use, which relies on seeding accounts with partisan videos, is simulating user behavior. Furthermore, the authors simply do not need this positioning, and it serves to distract from the main findings rather than illuminate them. In my opinion, the authors need to do substantial filtering down of their text to focus on presenting their main findings in as clear a way as possible, and try not to pontificate on general topics in the broader intellectual space (e.g., social media “harms”) that they cannot speak to. This is a major weak point of the manuscript as it is currently written. Rather than simply adding text to address the reviewer comments, the authors should focus the text down substantially to improve readability.

Response:

Thank you for highlighting the need for a more focused narrative arc for our manuscript, and we appreciate the opportunity to further revise our work to ensure that it meets the standards needed for publication. We agree that the earlier version of the manuscript included broader discussion that may have distracted from our central findings. To address this, we have streamlined the Abstract, Introduction, and Discussion to ensure that the main contribution is clearly emphasized throughout.

We believe that our primary contribution is that we conudct a large-scale, systematic audit of TikTok’s recommendation system during the 2024 U.S. presidential election cycle and show that TikTok’s recommendations exhibit a persistent Republican-leaning skew. Furthermore, we find

that this asymmetry is driven largely by negative-partisanship content (i.e., content that opposes or advocates against a party), and cannot be explained by observed engagement metrics such as likes, shares, or follower counts. We have revised the narrative arc of the paper so that our central contribution is foregrounded in the Introduction and again at the opening of the Discussion.

In addition, we have edited the reflections on our findings to keep them clearer and ensure that they are supported by our findings while limiting discussion on topics outside of the scope of our study. Our substantial editing pass reduced the word count of the Introduction and Discussion sections by ~1000 words, and we have taken the utmost care to incorporate your feedback. We believe this sharpened focus helped improve readability and makes it easier to discern the significance of our findings.

In addition, the authors do a poor job of discussing the effects to which they can actually speak. One of the reasons is the insistence to continue using 'negative partisanship' as their jargon of choice without properly defining it or separating it from related (but distinct) concepts. For example, as far as I can tell, the authors (e.g., in the abstract) refer to Democrats seeing anti-Democrat content as 'negative partisanship' when this term would typically refer to Democrats seeing anti-Republican content. They also refer to 'opposite-party recommendations' which further confuses multiple distinct phenomena (e.g., Democrats seeing pro-Republican content and Democrats seeing anti-Democratic content). There is no need for all this confusion, and it arises because the authors discuss things using abstract terms instead of speaking to the different kinds of effects that they uncover. They can simply refer to Democrats seeing pro-Democrat content, anti-Democrat content, pro-Republican content, or anti-Republican content (and do that same thing for Republicans) instead of lumping these into terms like negative partisanship and opposite-party recommendations, but if they do choose to use these terms then they need to define them precisely and stick to those definitions.

Response:

Thank you for these suggestions. We now cite the work of Iyengar & Ansolabehere [1] to motivate our usage of the term "negative-partisanship" when referring to **party opposing** content (i.e., Anti-Democratic or Anti-Republican). To avoid any potential confusion, we now explicitly state precisely what we mean by each of the constructs used in our analyses: (1) positive partisanship; (2) negative partisanship; (3) co-partisan recommendations; and (4) cross-partisan recommendations.

More specifically, following [1], we use the term "negative-partisanship" when describing the content of the message, irrespective of the viewer's partisanship. To put it differently, it is content that is opposing a party, rather than content that opposes one's own party. Under this scheme, a Democrat viewing an Anti-Democrat video is still coded as exposure to negative-partisan content. We agree that this interpretation needed to be explicitly stated early in the manuscript, as it indeed could lead to confusion otherwise.

In a similar spirit, we also adjust our usage of *opposite-party* recommendations to *cross-partisan* recommendations instead, to reduce the number of distinct terms used in our analysis. In total, this leaves us with the aforementioned four classification groupings.

To help clarify the terms we use in our analysis, we have added the following to the "Measuring Political Content" section on page 8 of the manuscript, before any usage of the term's negative or positive partisanship, and before discussing any substantive results:

We use a video's classification into one of Pro-Democratic, Anti-Democratic, Pro-Republican, Anti-Republican, or Neutral to define four analysis constructs:

- ***Positive partisanship** refers to content that **supports** a party or candidate (i.e., Pro-Democratic or Pro-Republican videos), irrespective of the viewer's own partisanship.*

- ***Negative partisanship** refers to content that **opposes** a party or candidate (i.e., Anti-Democratic or Anti-Republican videos), irrespective of the viewer's own partisanship.*

- ***Co-partisan** recommendations are videos whose partisan alignment **matches** a bot or viewer's own partisanship (i.e., Pro-Democratic and Anti-Republican videos for Democratic(-conditioned) viewers or bots; Pro-Republican and Anti-Democratic videos for Republican(-conditioned) viewers or bots).*

- ***Cross-partisan** recommendations are videos whose partisan alignment **opposes** the viewer's own partisanship.*

[1] - Iyengar, Shanto, and Stephen Ansolabehere. Going negative. Simon and Schuster, 2010.

Another concern arises from the new section that the authors added on validating their conditioning stage. While the authors argue that there is no skew in the videos they used for seeding, their own analysis shows the opposite and in the predicted direction – in particular, Figure 7A shows that Republican bots were more likely to be seeded with negative partisanship than positive partisanship, while the difference for Democratic bots was not statistically significant (or, at least, the confidence bands are overlapping). In general, this may be OK, but the authors need to acknowledge it and discuss it rather than pretend like these effects don't exist. In particular, it might be a problem if the authors went out of their way to seed videos that differed across parties. However, if in the task of selecting videos the authors came across more negative partisanship videos targeting Republicans than Democrats, that could be an artifact of the supply of content that is available – in which case, it may actually match the true conditions that real users are in when using TikTok.

Response:

Thank you for this point and we agree that the manuscript would benefit from a more detailed discussion and analysis of these results. To address your concern, we ran a regression

predicting the likelihood that a recommended video was negative-partisan. The results show that neither the bot's conditioning nor the proportion of negative-partisan videos in the conditioning stage significantly predicted whether recommendations had a negative tone. Instead, significant predictors were features of the video's author (follower count and total share count) and engagement with the video itself (normalized comment volume). This suggests that the gap in negative-partisan recommendations is better explained by supply-side dynamics than by factors controlled in our conditioning stage (see Supplementary Figure 7 below, where significant p-values after Bonferroni adjustment are highlighted in navy).

To describe this analysis and as a larger discussion of this asymmetry, we have added the following paragraph to the "Conditioning Stage Validation" section of the methods:

"Regarding positive and negative partisanship, while we find no statistically significant differences between the two bot groups (Positive partisanship: $t = 2.19$, $p = 0.129$; Negative partisanship: $t = -1.7$, $p = 0.373$; Neutral: $t = 1.63$, $p = 0.426$), we do nonetheless see a modest asymmetry, where the Republican seed set contained somewhat more anti-Democratic than pro-Republican content, while the Democratic seed set was comparatively balanced. Although this difference was not statistically significant, it is nonetheless important to acknowledge. This asymmetry does not reflect an intentional experimental design choice. Rather, it reflects the supply of content from the accounts selected for seeding, where Anti-Democratic videos were marginally more prevalent among the chosen Republican-aligned creators than Anti-Republican videos were among the chosen Democratic-aligned creators.

To assess whether this difference had downstream effects on the proportion of negative partisanship videos seen during the recommendation stage, we model the likelihood of a recommended video being of negative partisanship as a function of the proportion of such videos seen during a bot's conditioning stage. We find that it is neither the bot's conditioning nor the proportion of negative partisanship videos seen in the conditioning stage which significantly predict a recommended video being of negative partisanship. Rather, it is the characteristics of the video's author (their follower count and total share count), as well as the normalized number of comments the video receives which are significant, suggesting that the gap in negative partisanship recommendations is due to supply-side factors rather than factors in our control in the conditioning stage (see Supplementary Figure 7). Nonetheless, we recognize that this imbalance could contribute, at least partially, to the asymmetric outcomes we observe, and we highlight this as a limitation of our design."

We have also added the following paragraph to the limitations section:

"A further limitation concerns possible asymmetries in the conditioning stage of our audits. Although seed videos were comparable across partisan groups in semantics, partisanship, and engagement features, the source accounts differed: eight of the twelve Democratic seeds came from official office holders, while the Republican set skewed toward independent creators. If TikTok clusters users partly on latent account profiles or community

structures, our bots could inherit such differences despite conditioning on otherwise comparable videos. This possibility should be kept in mind when interpreting our results."

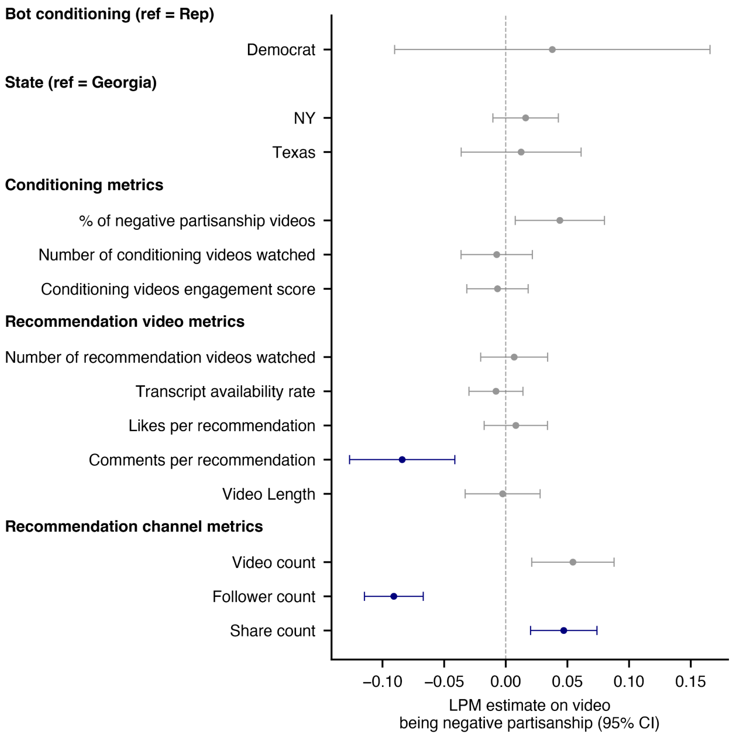

Supplementary Figure 7: Linear probability model estimates on the percentage point change in the probability of a recommended video being of negative partisanship. Statistically significant coefficients after Bonferroni multiple comparison adjustments are highlighted in navy.

Of course, this does put wrinkle on the effects that the authors find, and especially the finding that the Republican bots were recommended more videos containing negative partisanship than the Democratic bots. Are these effects due to the seeding asymmetry or to an algorithmic asymmetry? It is possible that we cannot know using the specific study that the authors conducted. But if the authors were upfront about this and left room for discussing this issue, it might be something that does not altogether invalidate the paper. Such a discussion may also be the appropriate place for a mention of the debate around user behavior vs. algorithmic filtering, since it is directly relevant here in a way that it isn't to the broader paper (which, again, I believe is about partisan asymmetries in algorithmic recommendations).

Another interesting finding that the authors could highlight more is that the asymmetries appear to increase as the election nears (i.e., see Figure 2 A, B, and C). If the seeding videos were the same across the different bots that were deployed over time, then the partisan asymmetry in seeding could not explain this trend - which may be one way of addressing the seeding asymmetry.

Response:

Thank you for this point. To your question as to whether the effects we see are due to seeding asymmetry or algorithmic assymetry, with regards to negative partisanship specifically, we note that we include the proportion of negative partisanship videos in the conditioning stage as a covariate in each of our regressions, where we find that it does have a small but significant effect on the number of cross-partisan videos recommended to the bot in the recommendation stage. We now make this clearer in our discussion of the results of our regressions. In addition, please see the above comment regarding the newly-added regression predicting the likelihood of a recommended video being of negative partisanship.

We also agree with your last point regarding how asymmetries increase as the election neared which was not discussed with enough detail in the manuscript, and further agree that this points to our conditioning protocol not having a significant effect on asymmetries in downstream recommendations. We add additional detail regarding this point when discussing the results of Figure 2 A,B, and C, which we copy below:

"Considering that the creator accounts used in the conditioning stage did not change over the duration of our experiments, there are several possible explanations for these findings. For instance, it is possible that the political debates in Congress surrounding TikTok led them to make algorithmic changes which reflect a partisan skew. Another may be that, as the election neared, users increasingly began to polarize in their content preferences as more and more politically uninterested or uninformed voters shaped their voting preferences."

Currently, there is a story to tell about partisan recommendations on TikTok that the authors tap into but the paper still falls way short of motivating and presenting in a clear and focused manner. There is simply too much discussion that is unrelated to the specific study that the authors present. Below, I include a few more specific comments to complement what I have already stated, and I would be happy to review a revised manuscript that takes these comments seriously and makes a significant effort to revise the paper in a way that makes for a clear and concise read.

Response:

Thank you again for your time. We assure you that we have taken your comments both in this review and in the previous one seriously and have made significant efforts to attempt and address your concerns, which we hope is reflected in the significant changes made to incorporate your feedback.

1) The addition of 'negative partisanship' to the title makes it less accessible to a general audience as most readers will be unfamiliar with the term. I suggest reworking the title to highlight the main findings in a clear manner and without jargon. That is, the title could be about anti-Democrat messaging to Republicans or something similar. Moreover, the hyphen (-) in negative-partisanship is odd and should be removed. I also suggest saying 'skew' instead of 'skewed' to imply that, unless something recently changed, the algorithm likely still functions in the way that the authors found.

Response:
We have revised the title as suggested. We opt for “skewed” rather than “skew” as we would like to focus our title on the 2024 US elections. The new title is: “TikTok's recommendations skewed toward oppositional and Republican-aligned content during the 2024 U.S. elections”

2) I find the sentence added to the abstract to be very confusing. To me, negative partisanship is NOT about Democrats seeing anti-Democrat messaging. Rather, negative partisanship means having Democrats see anti-Republican messages instead of pro-Democrat ones, or Republicans see anti-Democrat messages instead of pro-Republican ones. Judging from Figure 4 (A and B), the interesting effects (to me) include:

a. Republicans see more anti-Democrat messaging that Democrats see anti-Republican messaging (more negative partisanship for Republicans than Democrats)
b. Democrats see more anti-Democrat messaging than Republicans see anti-Republican messaging. I believe this is, perhaps, the effect mentioned in the abstract. I do not know what you call this – self-critical messaging, maybe? – but if the authors are going to focus on this effect then they should separate it conceptually from negative partisanship.

Response:
We agree with the reviewer about what the key interesting effects are from Figure 4. We have clarified the abstract to highlight these. We’ve also removed our usage of “negative-partisanship” from the abstract and only use the term after clarifying our definitions explicitly. The end of the abstract now contains the following, which we believe has improved the clarity of our findings:

We find significant asymmetries in content distribution. Republican-seeded accounts received approximately 11.5% more co-partisan recommendations than Democratic-seeded accounts. At the same time, Democratic-seeded accounts were exposed to roughly 7.5% more cross-partisan recommendations, driven disproportionately by anti-Democratic content. Two patterns stand out: (1) Republican-seeded accounts encountered more anti-Democratic videos than Democratic-seeded accounts encountered anti-Republican videos, and (2) Democratic-seeded accounts saw more anti-Democratic videos than Republicans saw anti-Republican videos. These asymmetries persist when accounting for video- and channel-level engagement metrics such as likes, views, shares, comments, and followers, indicating a Republican-leaning skew in recommendations that cannot be explained by the engagement measures we observe.

3) It is a bit weird to refer to ‘the type of partisanship’ videos reflect (e.g., page 3). In particular, videos don’t reflect negative or positive partisanship, since the partisanship of the audience will determine in part whether they are being exposed to negative partisanship. For example, a Democrat seeing a video that is anti-Democrat is not typically considered to be negative partisanship. A Republican viewing the same video would constitute negative partisanship. The authors could avoid all of this by not relying so much on the jargon of ‘negative partisanship’ and instead discussing things in simpler terms – people viewing information that is critical of their

opposite side vs. their own side, or information that is supportive of the opposite side vs. one's own side, instead of discussing ‘types of partisanship’ which makes little sense.

Response:

Thank you for these points. Specifically with regards to the phrase “type of partisanship”, we’ve now changed the wording both on page 3, and in several other parts of the manuscript to clearer language. For example, in the Discussion, we previously said:

“We also find that the asymmetries in exposure are not symmetrical in partisan content types.”

Instead, we now say:

“Partisan asymmetries also extend to the tone of recommended content.”

We refer back to our previous comment regarding our inclusion of explicit defintions for negative and positive partisanship early in the manuscript to help guide the reader towards understanding our analyses.

Response to the Editor

We thank you and the reviewers for the time and care invested in evaluating our manuscript. We have revised the manuscript to address the framing and presentation-related concerns raised by Reviewers 2, 3, and 5.

Below, we summarize the changes we have made in response to the points you highlighted.

1. Concerns raised by Reviewers 2 and 3

In line with the reviewers' minor feedback and suggestions, we strengthened and clarified our methodological analyses in several ways:

a. Clarifying the scope of causal inference and user-centered explanations

The paragraph now includes the precise caveat suggested by the reviewers:

"By systematically ruling out plausible user-centered explanations, ***to the extent possible with the available data,***…"

b. Figure-labeling corrections

We corrected inconsistencies in axis labeling, ordering of conditioning metrics, and formatting across Figures 2H, 3C, and 4E, ensuring alignment and readability.

c. Multiple-comparison corrections

We revised the handling of multiple-comparison adjustments in response to Reviewers 2 and 3's suggestions:

1. We now use Benjamini–Hochberg (BH) corrections exclusively in the main figures (2H, 3C, 4E).
2. Significance indicators in the figures now correspond to BH-adjusted p-values, not unadjusted p-values.
3. The main text explains why BH is appropriate for our testing context.
4. We maintain transparency by reporting both unadjusted and BH-adjusted p-values in Supplementary Tables 15–20.

2. Concerns raised by Reviewer 5

We undertook substantial revisions to address Reviewer 5's remaining concerns about conceptual framing, structure, and clarity.

a. Rewriting and shortening the abstract

The abstract has been significantly revised to:

1. Remove redundancies and improve clarity.
2. Present the main audit findings once, concisely.
3. Integrate and motivate the top-account, topic-modeling, and survey analyses within the paper's overarching research question.
4. Eliminate the prior sense that these analyses appeared "out of nowhere."

b. Removing mentions of affective polarization

To clarify, our previous inclusion of the literature on affective polarization in the Introduction was to give a broad overview of the literature on polarization as a whole and to clarify differences between affective and content polarization, rather than to discuss the concept as a preview of our own audit and analyses. That said, we agree with the reviewer that this perhaps was unnecessary, and in response, we now focus our introduction solely on algorithmic amplification, content polarization, and exposure asymmetries produced by recommendation systems.

The subsection title has been changed to:

"Social Media, Algorithmic Recommendations, and Content Polarization."

c. Relocating limitations material

We revised the Introduction so that early mentions of limitations now appear only as brief scope-setting statements. Full discussion of limitations is included at the end of the paper, where it can be interpreted in light of the study design and results.

d. Clarifying Figure 2 interpretation

We clarified in both the caption and main text that the middle panels of Figure 2 plot the difference between Republican and Democratic ideological content, which can remain flat even as the gap between raw levels widens. This explanation should eliminate the confusion noted by Reviewer 5.

e. Linear probability models vs. logistic regression

In response to Reviewer 5's question regarding model choice:

1. We retained LPMs in the main text for interpretability.
2. We re-estimated all models using logistic regressions and report them in Supplementary Tables 16, 18, and 20.
3. The Methods section now includes a new paragraph explaining this choice and confirming the robustness of our findings across estimators.

f. Streamlining robustness checks in the main text

We have condensed the robustness and sensitivity analyses in the main text, relocating details to the Methods or Supplementary Materials.

g. Correction of figure captions and terminology

1. Removed “over time” from Figure 4’s caption, as the reviewer noted no time axis was present.
2. Ensured consistent use of defined terminology (e.g., replacing “negative out-party content” with “anti-Democratic content”).
3. Clarified the x-axis of Figure 5 and relabeled “mismatch proportion” to a more interpretable “Proportion of cross-partisan recommendations.”

Thank you once again for your time, and we hope that the changes will satisfy both you and the reviewers.

Reviewer #2 and #3:

We thank the authors for their response, which addressed many of our concerns. We recommend making a few minor changes in consultation with the editor before proceeding to publication.

Response:

We would like to thank both Reviewer 2 and 3 for their comments and suggestions throughout this review process. We are happy that the changes made in the previous revision largely satisfied most of your concerns, and we hope that our responses below effectively satisfy any remaining concerns you still have.

1. Why do the authors think this is happening?

With the authors’ edits to the abstract, introduction, and the contributions and political recommendation rates sections, our concerns that the paper too strongly implied that TikTok’s algorithm itself was the cause of the partisan asymmetries—beyond the scope of the design—have largely been addressed. We also appreciate the clearer early definition of skew and the replacement of “bias” with “skew” throughout.

One of the new paragraphs added to the “Our Contributions” section states that the “plausible user-centered explanations” are “ruled out.” This omits the necessary caveat (noted in our earlier review of the Sensitivity Analysis section) that the observed skew could still reflect unobserved internal engagement metrics (e.g., dwell time). A small edit to the new paragraph would resolve this concern (edits indicated with *):

Our findings reveal a significant Republican-leaning skew in TikTok’s recommendations—one that persists across states even after accounting for channel- and video-level engagement metrics. By systematically ruling out plausible user-centered explanations ***to the extent possible with the available data***, we provide evidence that the asymmetries we observe may be influenced, at least partially, by the algorithm’s governing rules, though not necessarily by intentional design choices.

Response:

Thank you for this comment. The paragraph has now been adjusted as per your suggestion. The sentence now reads as follows:

“By systematically ruling out plausible user-centered explanations, to the extent possible with the available data, we provide evidence that the asymmetries we observe may be influenced by the algorithm’s governing rules, though not necessarily by intentional design choices.”

Outside of this small concern, we are satisfied with the authors’ edits.

2. Alternative explanations for asymmetry

We asked the authors to acknowledge that unobservable asymmetries may remain in the conditioning stage, specifically that Democratic seeds were disproportionately elected officials, while Republican seeds skewed toward independent creators. We suggested the authors warn that latent account-level clustering could influence results. The authors added cautionary notes to the Experimental Setup and Limitations sections explaining this possibility, which we believe clarify the limits of the inferences they can make and improve the transparency of the study’s design. We are satisfied with these edits.

Response:

Thank you for your comments, and we are glad that our revisions addressed your concerns.

2a. Party x negativity interactions

We suggested that the authors estimate models with party x negativity interactions. The authors clarified that they had already run such interaction models, and they updated the manuscript to make this explicit. They also added engagement metrics broken out by the five categories (Pro-Dem, Anti-Dem, Pro-Rep, Anti-Rep, Neutral) in the supplementary materials. We are satisfied with these substantive changes.

We did notice a few very minor labeling inconsistencies in relevant figures that could be corrected for clarity:

Inconsistency in label wording:
-Figure 2H: “% of negative partisanship videos” vs. Figures 4E & 3C: “Proportion of negative partisanship videos.”
-Inconsistency in label ordering under “Conditioning Metrics”:
-“Number of conditioning videos watched” appears third in Figures 4E & 3C but second in Figure 2H.
-“Conditioning videos engagement score” appears second in Figure 4E but third in Figure 2H.
-Minor formatting: “Length” is capitalized in “Video Length” across Figures 4E, 3C, and 2H.

These are only aesthetic issues and do not affect the results, but fixing them would improve consistency and interpretation across figures.

Response:

We appreciate your careful reading and for pointing out these labeling inconsistencies. Each of these issues has now been addressed.

3. Other points: Should the authors be using a false discovery rate correction?

We asked the authors to clarify where Bonferroni corrections were applied, why they were chosen over other adjustments, and to provide both adjusted and unadjusted p-values.

The authors have made progress: Supplementary Tables 15–17 now report unadjusted, Bonferroni, and Benjamini–Hochberg (BH) adjusted p-values, and figure captions for Figures 2H, 3C, and 4E note that multiple-comparison corrections were applied.

However, several points remain unclear:

-Justification: The manuscript does not explain why both Bonferroni (family-wise error rate) and BH (false discovery rate) corrections are used or why they are treated interchangeably. Family-wise error rate controls the probability of any false positive result across all tests, whereas false discovery rate controls the expected proportion of false positives among the rejected hypotheses. These are conceptually distinct approaches; using both without explanation is confusing.

-Which p-values define significance in figures: It appears Figures 2H, 3C, and 4E highlight coefficients as significant based on unadjusted p-values, even when the corresponding Bonferroni and BH values are not below 0.05. For example, in Supplementary Table 15, "Proportion of negative partisanship videos" and "Number of conditioning videos watched" are statistically significant when unadjusted but not after correction, yet Figure 2H presents them as significant.

-Inconsistencies between Bonferroni and BH: In one case (Video count under Recommendation channel metrics, Table 15), Bonferroni and BH differ in whether a result is considered significant, but the manuscript gives no guidance on how to interpret such disagreements.
While we agree with the authors that the multiple comparison results do not affect the paper's substantive conclusions, the current presentation is confusing. If they proceed with using both adjustments, we recommend that the authors briefly explain in the main text or appendix (a) why they use both Bonferroni and BH corrections, (b) which set of p-values (unadjusted, Bonferroni, or BH) underlies the significance markers in Figures 2H, 3C, and 4E, and (c) how they handle disagreements between the two comparisons. It isn't obvious to us why using both would be necessary, so we would advise using BH as it is more appropriate when the goal is to limit the expected proportion of false discoveries rather than the probability of any false discovery.

Response:

We have now adjusted each of the regression figures (2H, 3C, 4E) so that significance markers are based on p-values after Benjamini–Hochberg (BH) correction, and we clarify this in the captions of each figure. As per your suggestion, we now rely exclusively on BH corrections, rather than reporting both Bonferroni and BH adjustments. Unadjusted and BH-adjusted p-values are reported in Supplementary Tables 15–17 for transparency, and in the main text we explain why we chose to use BH corrections when first introducing these models.

Thank you once again for your time and support throughout this revision process.

Referee #5:

In their second revision of "TikTok's recommendations skewed toward oppositional and Republican-aligned content during the 2024 U.S. elections," the authors make some welcome edits to their previous manuscript. The improved focus of the manuscript is noticeable, and I appreciate the lengths to which the team went to improve the readability of the paper. I believe this round of edits was much more successful than the previous round, and I encourage the authors to continue focusing on the specific theories to which the authors can speak to and the actual contributions their study makes.

I still believe that there are several issues with the way that the study is motivated, the data are presented, and the amount of unnecessary language that remains in the manuscript for a journal such as Nature. Below, I highlight where I believe some further interventions are needed.

Response:

We thank you for carefully rereading our manuscript and for your encouraging comments about the improved focus and readability of this revision. We appreciate your recognition of our efforts to sharpen the theoretical framing and clarify the study's contributions. We also take seriously your remaining concerns about the motivation, data presentation, and unnecessary language, and we have made further targeted revisions along these lines. We address each of your points in detail below.

1) the abstract is quite wordy and yet still does not address the findings of the analysis of top accounts or the survey or topic modeling section. These additional analyses thus seem to come out of nowhere, are not motivated, and their findings are not properly placed within aims specified by the authors early on in the paper. I suggest either disposing of these findings or including them in the abstract as well as motivating them in the introductory portions of the paper. As of now, it still feels like these descriptive analyses are motivated by data availability and not theoretical or methodological contributions. The abstract also feels repetitive, with the main findings stated twice (once with percentages and once without) but with different wording. Such slopinness is unfortunate for a manuscript that is this late in the review process.

Response:

We thank the reviewer for this helpful set of comments. We agree that the prior abstract was too long, repeated some ideas, and did not sufficiently integrate or motivate the top-account, survey, and topic-modeling analyses. We appreciate the opportunity to improve its clarity and structure.

In response, we have substantially revised and shortened the abstract, bringing it to about 150 words, or the length recommended by Nature's guidelines. The new version removes redundant wording, presents the main audit findings in a single, concise statement, and integrates the top-account, survey, and topic-modeling results so that these analyses are clearly situated within the paper's aims rather than appearing ad hoc. The revised abstract now explicitly frames all ancillary analyses as extensions of our central question: whether partisan asymmetries in TikTok's recommendations arise symmetrically, asymmetrically, or in a partisan-skewed manner. The revised abstract now reads as follows:

"Social media is widely seen as fueling partisan animosity, yet it remains contested whether polarization reflects users' self-selection or algorithmic curation. We examine this question on TikTok, where an algorithmic feed structures most political information exposure. Using 323 audit experiments with controlled ``sock-puppet" accounts seeded with Democratic or Republican content across three

states, we collect over 280,000 recommendations. TikTok displays clear asymmetries: Republican-seeded accounts receive about 11.5% more co-partisan content, while Democratic-seeded accounts see roughly 7.5% more cross-partisan content—largely anti-Democratic—even after adjusting for engagement. High-reach Republican channels are recommended to Democrats more often than comparable Democratic channels are recommended to Republicans, and topic patterns show that asymmetries cluster in specific domains: Democrats receive cross-partisan content mainly on immigration, crime, and foreign policy, while Republicans' cross-partisan exposure centers on abortion. A survey of 1,008 U.S. users confirms these disparities are visible to users. Together, the results indicate a systematic, oppositional, and Republican-leaning skew in TikTok's political recommendations."

To ensure alignment, we also amended the text in the Introduction relating to these additional analyses, explaining why they complement the audit and help interpret the mechanisms and lived experience of the partisan asymmetries we identify.

We hope these changes address your concerns and strengthen the manuscript's coherence and contribution.

2) the early focus on affective polarization is odd, and especially in the subsection heading, since the article does not use any measures typical in this literature (thermometer ratings obtained through surveys). Incidentally, these could have been nice measures to add as the team did launch a survey, but no matter. The proper framing would seem to be prior work on algorithmic amplification rather than psychometric measures that the paper cannot speak to. A better section heading would be Social Media, Something, and Content Polarization where "Something" might be about Negativity, Political Orientation, Partisanship, Algorithmic Recommendations, or some other construct that is actually addressed in the analyses.

Response:

Thank you for this helpful suggestion. We agree that our previous framing placed too much emphasis on *affective* polarization, despite the fact that our study does not measure it directly. Our empirical analyses speak to content polarization and algorithmic recommendations, not to attitudinal or thermometric indicators typically used in the affective polarization literature.

In response, we have revised the subsection heading to better match the scope of our contribution. We now title the section "**Social Media, Algorithmic Recommendations, and Content Polarization."**

We have also adjusted the opening paragraphs to clarify that our focus is on how recommendation systems shape exposure to political content. We believe this improves the conceptual alignment between the literature review and the analyses that follow and hope you find the revised framing clearer and more appropriate.

3) I appreciate that the authors note limitations early on in the manuscript, but I believe they should not preemptively do this but rather report the actual findings first without unnecessary pontificating. Then, they should leave discussion of limitations until after the design and results have been discussed so that these limitations can be properly assessed by readers of the paper.

Response:

We appreciate this suggestion regarding the placement of our limitations. In an earlier revision, we included more detailed limitations discussions closer to the front of the paper in response to concerns from another reviewer that these caveats were not sufficiently foregrounded. We agree, however, that extended discussion of limitations may be best evaluated in light of the study design and results.

In the revised manuscript, we therefore streamline the early mention of limitations to a brief set of statements that outline the main scope conditions of our findings and direct readers to the limitations section.

4) I am confused about Figure 2. The gap between Dems and Reps clearly increases over time in the top panels, but the middle panels show a straight line. Shouldn't they be increasing?

Response:

We appreciate the reviewer's close reading of Figure 2 and understand the source of confusion. In the top panels, the y-axis shows our *ideological content* score separately for Democratic and Republican bots. This measure is positive when the recommended content is more Republican-leaning and negative when it is more Democratic-leaning. Over time, the Republican bots see increasingly Republican content (their line moves up), and the Democratic bots see increasingly Democratic content (their line moves down), so the gap between the two lines widens.

By contrast, the middle panels do not show these two series separately. Instead, they display *ideological skew*, which we define as the difference in ideological content between Republican and Democratic bots (Republican bots' score minus Democratic bots' score). Because both groups' ideological content scores are moving away from zero at roughly the same rate but in opposite directions, this difference remains approximately constant over time, which is why the middle panels appear relatively flat even as the top-panel gap widens.

To make this clearer, we have revised the caption and surrounding text for Figure 2 to (a) explicitly state that the middle panels plot the difference between Republican and Democratic bots' ideological content scores, and (b) briefly explain why a widening gap in the top panels can coexist with a roughly flat line in the middle panels. Specifically, we've adjusted the wording in the relevant paragraph as follows:

"Figures 2D, E, and F collapse these party-specific trends into a single measure of "ideological skew" for each state and week. Here, the y-axis shows the difference in ideological content between Republican- and Democratic-conditioned bots (the mean ideological content score for Republican bots minus the mean score for Democratic bots), where negative values indicate a Democratic skew and positive values indicate a Republican skew. Because both Democratic and Republican bots are moving away from zero in opposite directions at roughly similar rates in panels A–C, their difference in panels D–F remains relatively stable over time even as the gap between the two lines in the top panels widens."

5) Why use LPMs instead of logit or probit? If there were interactions in the model then perhaps an LPM would be reasonable, as interaction terms in nonlinear models are more difficult to interpret and depend on other covariates. However, in most of the models there are no interaction terms, as far as i can tell. More justification using the appropriate literature is needed here.

Response:

Thank you for this suggestion. For completeness, we now re-estimate all models using logistic regression and report these results in Supplementary Tables 16, 18, and 20. As noted in the Methods, the sign and

substantive significance of the key coefficients are largely similar in the logit and LPM specifications, indicating that our conclusions are not sensitive to the choice of functional form. We add the following paragraph to the Methods section:

"For ease of interpretation, the main-text regression figures report results from linear probability models (LPMs), whose coefficients can be read directly as percentage-point changes in the outcome (Supplementary Tables 15, 17, and 19). To assess robustness to functional-form assumptions, we also estimate the same specifications using logistic regression and report these results in Supplementary Tables 16, 18, and 20. Across all models, the sign and substantive significance of the key coefficients are largely similar in the logit and LPM specifications."

6) There appear to be too many robustness checks and sensitivity analyses in the main text for a Nature article. I might mention these briefly in the main body and move most of the text to the appendix.

Response:

We thank you for this suggestion. In revising the manuscript, we have significantly streamlined the presentation of robustness checks and sensitivity analyses in the main text, briefly summarizing the key results and moving additional detail to the Methods section. We are also happy to relocate further material to the appendix if the editor feels that a more compact main text is preferable for *Nature*, and we defer to the editor's judgment on the most appropriate balance between brevity and transparency.

7) Figure 4 says that we should expect data to be shown "over time" but none of the plots appear to have a time axis

Response:

You are absolutely correct here. We've adjusted the figure caption to remove the phrase "over time."

8) what is "negative out-party content" (p. 21)? The authors have been using anti-Democratic/Republican, co/cross-partisan, etc. and should make sure to stick to the terms they have actually defined throughout

Response:

Thank you for pointing out this inconsistency in our terminology. We have replaced "negative out-party content" with "Anti-Democratic content" to align with the categories we define earlier in the manuscript (e.g., anti-democratic/anti-republican). We have also reviewed the text to ensure that we consistently use only terms that are explicitly defined. The sentence now reads as follows:

"This confirms that the partisan skew we document is not simply Republican versus Democratic, or positive versus negative, but specifically concentrated in Anti-Democratic content."

9) It is unclear how the comparison is made in the x-axis label of Figure 5. It would help if the authors included the actual metrics they use as axis labels. This also holds for "mismatch proportion" - the plots should be readable without reference to the manuscript text.

Response:

Thank you for pointing out that the x-axis label in Figure 5 was unclear. In the revised manuscript, we have updated the x-axis label to "Estimated difference in outcome (Republican respondents −

Democratic respondents)" and added a note in the caption clarifying that each point represents the regression-estimated difference in the outcome between Republican and Democratic respondents, controlling for demographic covariates. This should make the comparison interpretable without referring back to the main text. With regard to "mismatch proportion," we've now adjusted the corresponding labels to "Proportion of cross-partisan recommendations" and have also changed the x-axis labels for the models to be "Change in probability of viewing a cross-partisan recommendation (95% CI)."

Thank you once again for your time and support throughout this revision process.

Response to the Editor:

Dear Sebastian,

Below are our responses to the remaining comments by Referee #6. We believe all his comments have been addressed and we look forward to getting this paper published!

Referee #6 (Remarks to the Author):

Request 1: A previous review commented on the abstract and requested substantial changes. I believe the edits to the abstract now ensure it is clear and concise and related edits in the introduction makes it clear why each of the different analyses are included. However, the opening sentence of the abstract suggests the study will examine self-selection versus algorithmic curation, yet this study actually only looks at algorithmic curation. As such, a very small re-frame of the first sentence is requested.

We have adjusted the first two sentences of the abstract to read as follows:

"Social media platforms increasingly mediate political information exposure, yet the role of algorithmic curation in shaping political exposure remains contested [1, 2]. This question is difficult to resolve on platforms where users retain substantial control over their feeds [3, 4]."

Request 2: That said, in my review of the methods, I did note a new item. It is highly irregular in my field to report survey results before describing the sample of respondents. On page 20 the 1008 respondents are described as "U.S.-based TikTok users" but nothing is said about whether the sample was random, representative, or what the demographic breakdown of the sample actually was. I see this as essential information for interpreting the results. I am not suggesting table 28 in the supplemental materials need to be move to the main body, but I do think a single sentence describing the sample and a reference to the table in the supplemental materials is appropriate here. That said, I will defer to the standard approach for the journal and rely on the editors to make the final call.

We have added the following sentence before introducing the survey results describing the demographic breakdown of the survey participants.

"Participants (N = 1,008) were recruited via Prolific from U.S.-based active TikTok users; the sample was not nationally representative and skewed Republican (54.8% Republican, 31.4% Democratic, 13.8% Independent), with 54.3% female, 44.5% male, and 1.2% non-binary respondents; 64.1% identified as White and 27.4% as Black; 82.2% held at least a bachelor's degree; and the largest age group was 25–34 years old (37.0%). Full demographic details are reported in Supplementary Table 28."

Request 3: While reviewing these I did also note an accessibility concern. Many of the figures and graphs are unlikely to be accessible for people who are colourblind and for people who rely on grey-scale settings. I recommend all figures and graphs be examined to ensure they are accessible. Using different patterns or shapes in addition to the existing colour variation is likely the easiest way to accomplish this.

We have now adjusted Figure 2A-C to include different shapes for each of the bot conditions. We've also adjusted Figure 3A and B (which are now moved to the SI) to include different hatches for box plots corresponding to Democratic and Republican channels. As far as we can tell, the remaining graphs and figures should be color-blind accessible, as each set of datapoints corresponding to a particular group are either of a sufficiently different color (such that it is clear in grey-scale), a different shape, or are clearly labelled along either the x- or y-axes.

Next, I offer three optional suggestions related to the discussion of limitations:
Request 4: While the authors do appropriately point to the limits of using LLMs to analyze content and of relying on only transcripts and not visual cues, there is no mention of the limits of focusing on exclusively English content. A brief mention of this limitation reflecting would be useful given that the political information sharing on TikTok in non-English languages in the US might reasonably be different given that it likely comes from minority groups who may use and be impacted by the platform differently.

We have added the following sentence to the first paragraph in the Limitations section:

"Fourth, our analysis focuses exclusively on English-language content, as our transcript-based pipeline relies on English subtitles. We therefore do not capture political videos in Spanish or other languages that may circulate within linguistic minority communities in the United States. Recommendation dynamics and partisan exposure patterns in these information environments may differ, limiting the generalizability of our findings beyond English-language political content."

Request 5: 75% seems like a high threshold for amount of content that is partisan aligned – many creators add in content that is not political or is more neutral in order to be able to monetize, avoid de-prioritization, or to perform authenticity. Ideally, I'd like to see a sentence on page 9 in the top paragraph commenting on the high threshold for what counts as a partisan account or a line added to the limitations section.

We now add the following sentence where we discuss the threshold, stating:

"We adopt this relatively high threshold to ensure that classified channels are consistently partisan in their output, recognizing that many creators intersperse political content with non-political or more neutral material for many reasons such as monetization or audience growth. A stricter cutoff reduces the risk of misclassifying mixed-content creators as partisan based on a small subset of videos."
We note that this section has also been moved to the SI.

Request 6: The bots were trained in a similar way in order to establish an ideological stance for each sock puppet, but this assumes that actual Democrats and Republicans would consume content in the same way. However, we know there are partisan differences in political information seeking practices and exposure via social media and it is therefore possible that training the sock puppets in the same way reduces external validity. Consider noting this as a limitation.

We added the following sentences to the second paragraph of the limitations section:

"Furthermore, our standardized conditioning protocol also abstracts from real-world differences in how Democrats and Republicans engage with political content online. Prior research suggests that partisan groups differ in information-seeking behavior and interaction patterns; by holding engagement constant across bots, we isolate algorithmic responses to partisan signals but do not capture how naturally occurring behavioral differences may shape recommendation dynamics."

Request 7: Finally, the bibliography, while including an appropriate range of sources, contains many capitalization errors such as journal names and proper nouns. Beyond standard editing, please also note danah boyd is spelt all lower case.

This has now been fixed!
